# Photoactivatable Plant Hormone-Based Chemical Inducers of Proximity for *In Vivo* Applications

**DOI:** 10.1021/acschembio.4c00592

**Published:** 2025-01-27

**Authors:** Philipp Pöschko, Caroline M. Berrou, Kaisa Pakari, Michael J. Ziegler, Christoph Kern, Birgit Koch, Joachim Wittbrodt, Richard Wombacher

**Affiliations:** † Department of Chemical Biology, Max Planck Institute for Medical Research, Jahnstraße 29, 69120 Heidelberg, Germany; ‡ Faculty of Biosciences, Heidelberg University, Im Neuenheimer Feld 234, 69120 Heidelberg, Germany; § Centre for Organismal Studies Heidelberg (COS), Heidelberg University, Im Neuenheimer Feld 230, 69120 Heidelberg, Germany; ∥ Heidelberg Biosciences International Graduate School (HBIGS), Heidelberg University, Im Neuenheimer Feld 501, 69120 Heidelberg, Germany

## Abstract

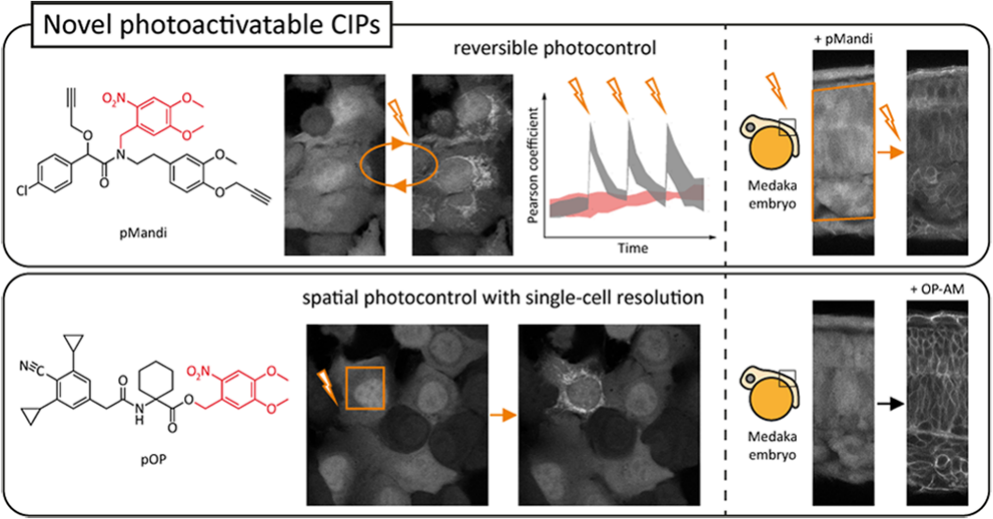

Protein interactions play a crucial role in regulating cellular mechanisms, highlighting the need for effective methods to control these processes. In this regard, chemical inducers of proximity (CIPs) offer a promising approach to precisely manipulate protein–protein interactions in live cells and *in vivo*. In this study, we introduce pMandi, a photocaged version of the plant hormone-based CIP mandipropamid (Mandi), which allows the use of light as an external trigger to induce protein proximity in live mammalian cells. Furthermore, we present opabactin (OP) as a new plant hormone-based CIP that is effective in live mammalian cells at low nanomolar concentration and in live medaka embryos at submicromolar concentration. Its photocaged derivative, pOP, enables the induction of protein proximity upon light exposure in individual cells, enhancing spatiotemporal control to the level of single-cell resolution. Additionally, we explored the use of both photocaged CIPs to promote protein proximity in live medaka embryos.

## Introduction

Protein proximity is essential for the regulation of many cellular mechanisms. Therefore, methods to specifically control individual protein–protein interactions and proximity are of high interest. A widely used tool to manipulate cellular processes that depend on protein proximity are so-called chemical inducers of proximity (CIPs).^1^ They have been used in the context of protein degradation, protein transport, gene regulation signal transduction, and cellular therapies.^1^ CIPs are small molecules that induce the dimerization of two specific protein domains. Proteins that are expressed as fusion proteins with these dimerizing protein domains can thus be brought into close proximity upon the addition of the CIP. This approach is versatile, as there is no need for ligands for each specific protein of interest.

A basic distinction is made between homodimerizers, CIPs that bring the same proteins into close proximity, and heterodimerizers, which bring two different proteins into close proximity. Such heterodimerizing CIPs can be further distinguished into two groups: bivalent and monovalent molecules. Bivalent molecules, such as the immunosuppressant-based CIP rapamycin^2^ and its analogs (rapalogs), function by dimerizing the proteins FRB and FKBP12, possess binding sites on both interacting proteins. This class of molecules is often referred to as molecular glues^3^ and also includes proteolysis-targeting chimeras (PROTACs). In contrast, monovalent CIP molecules, like most plant hormone-based CIPs, interact with only one receptor protein and induce a conformational change that creates a binding site for a second receiver protein in a molecular ratchet-type mechanism.^3^ Therefore, compared to systems based on bivalent molecules, these systems show no Hook effect and only a negligible background association in the absence of the CIP. Moreover, plant hormone-based CIP systems are orthogonal in mammalian cells and therefore attractive for use *in vivo*.

In the past years, it has been shown that the plant hormones abscisic acid (ABA)^4^ dimerizing the proteins PYL and ABI, gibberellic acid^5^ dimerizing the proteins GID1 and GAI (both molecular ratchet-type), as well as auxin^6^ dimerizing the proteins TIR1 and AID (molecular glue-type) can be used as CIPs in mammalian cells. However, these plant hormone-based CIP systems require a high working concentration of the CIP, often in the high micromolar range, which significantly limits their application *in vivo*.^7,8^ Hence, there is a need for biorthogonal CIP systems that require low working concentrations.

To improve the control of protein manipulation mediated by CIPs in space and time, they have been brought under the control of light by photocaging. Photoactivation of CIPs is an attractive way to gain spatiotemporal control over the process and to induce proximity upon irradiation with light.^9−11^ Photoactivatable derivatives of rapamycin have been developed^12−15^ but suffer from the nonorthogonality of rapamycin in mammalian cells and *in vivo*. Likewise, photocaged ABA,^16^ photocaged gibberellic acid^17,18^ and photocaged auxin^19,20^ were designed concerning the use as photoactivatable CIPs but require rather high working concentrations. In addition, bivalent CIPs based on protein tags that can be either activated or deactivated upon irradiation of light were developed, with which protein manipulations can be carried out with high spatial control.^21−23^ However, the working concentration of such CIPs must be chosen carefully to avoid a loss of activity due to the Hook effect,^24^ an inherent challenge for bivalent dimerizer.^25^

Here, we present two novel plant hormone-based photocaged CIPs that allow the induction of protein proximity with light as an external trigger. We show their application in live mammalian cells at nanomolar concentrations and in live medaka embryos at low micromolar concentrations.

## Results and Discussion

**Figure 1:**
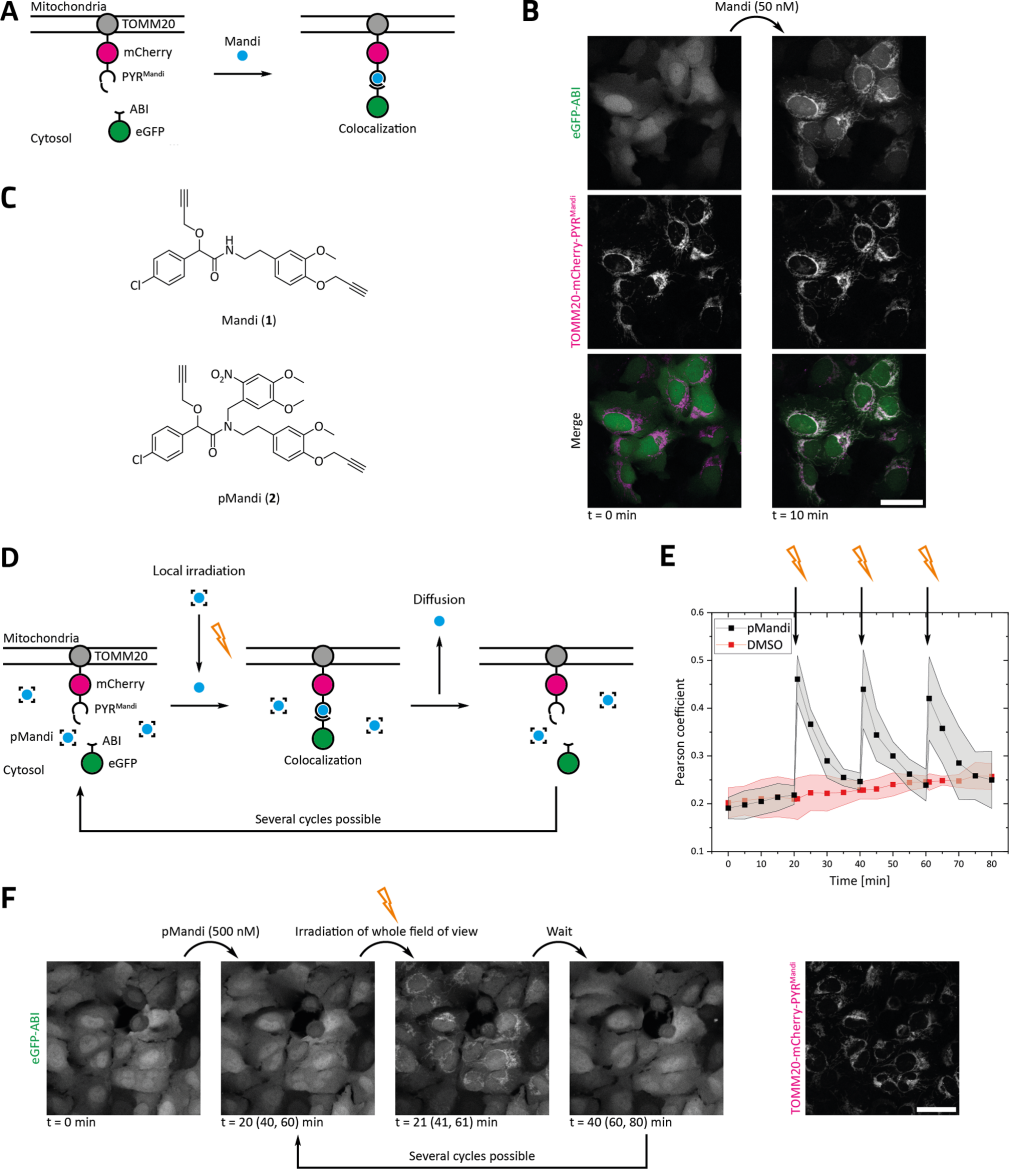
Inducing protein proximity in live cells using mandipropamid (Mandi) and photocaged mandipropamid (pMandi). (A) Schematic representation of the used protein constructs and the colocalization assay. (B) Mandi induces protein colocalization at low nanomolar concentration in cellulo. Confocal microscopy images of U2OS FlpIN cells stably expressing TOMM20-mCherry-PYR^Mandi^ and eGFP-ABI before and 10 min after addition of 50 nM Mandi. Scale bar: 40 μm. Representative data were obtained from three experiments. (C) Structures of Mandi and pMandi. (D) Schematic representation of the colocalization assay with pMandi. pMandi enables the induction of protein proximity upon irradiation. Local irradiation leads to local release of the active molecule Mandi and induction of protein proximity. Due to its high permeability, the active molecule Mandi diffuses out of the irradiated area and the colocalization is reversed. Several cycles are possible. (E) Pearson coefficient of the eGFP and mCherry channels derived from confocal microscopy images of U2OS FlpIN cells stably expressing TOMM20-mCherry-PYR^Mandi^ and eGFP-ABI. The whole field of view was irradiated (405 nm) at 20, 40 and 60 min immediately after image acquisition. Cells were treated with either 500 nM pMandi or DMSO at 0 min. Average and standard deviation of the mean were obtained from each four experiments per condition. (F) Confocal microscopy images of U2OS FlpIN cells stably expressing TOMM20-mCherry-PYR^Mandi^ and eGFP-ABI at different time points before and after addition of 500 nM pMandi and after irradiation (405 nm). Irradiation was performed immediately after image acquisition at 20 min after addition of pMandi. Images are representative for repeated irradiation at 40 and 60 min immediately after image acquisition. Scale bar: 40 μm. Representative data were obtained from four experiments.

We have recently reported mandipropamid (Mandi), a novel plant hormone-based CIP. Mandi dimerizes PYR^Mandi^, a mutant of the plant-originated abscisic acid receptor protein PYR, and the receiver protein ABI at low (nanomolar) working concentration. Its excellent cell permeability and low toxicity make the system very suitable for *in vivo* applications.^26^ To further improve the spatiotemporal control of the dimerization process, we synthesized the inactive, photocaged mandipropamid derivative pMandi (Figure 1C) that can be efficiently converted to active Mandi by irradiation with light. pMandi has a dimethoxynitrobenzyl photocaging group at the nitrogen atom of the amide bond of Mandi and was synthesized via a convergent synthesis from commercially available compounds (for synthesis description and synthesis scheme, see Section S2.7.4).^27^ The photocage can be cleaved upon irradiation with UV and blue light (365 and 405 nm) and releases the active molecule Mandi without undesired side products and any scar originating from the attachment of the photocage (Figures S1 and S2).

We chose the dimethoxynitrobenzyl group as the photocage in this work since it is stable at the excitation wavelength of GFP (488 nm) and also shows good stability under normal laboratory lighting, which facilitates the handling of the photocaged molecules. Further, its small molecular size prevents issues with solubility and permeability, important criteria for in vivo applications. The quantum yield of the decay of pMandi upon light irradiation (405 nm) was determined to Φ = 2.2% (Figure S3).

To characterize the background activity of pMandi without irradiation, we performed a cellular luciferase transcription assay and demonstrated that pMandi is three orders of magnitude less potent than Mandi in inducing protein proximity after overnight incubation in the dark (Figure S4). Upon irradiation of cells treated with pMandi and successive overnight incubation, luciferase expression can be induced in a light dose-dependent manner (Figure S5).

Next, we used a colocalization assay with fluorescent proteins to investigate the spatiotemporal control of the dimerization process in live mammalian cells induced by pMandi upon light irradiation (Figure 1A). We expressed PYR^Mandi^ as a fusion protein with mCherry and TOMM20, as well as cytosolic eGFP-ABI in U2OS cells. Dimerization of PYR^Mandi^ and ABI results in the colocalization of mCherry and eGFP fluorescence at the outer mitochondrial membrane. While addition of Mandi induces fluorescence colocalization at nanomolar concentration within a few minutes (Figures 1B and S6), we did not observe colocalization for pMandi without irradiation (Figure 1F) even after several hours of incubation (Figure S7). To demonstrate photoactivation in live cells, we first performed light irradiation of cells treated with pMandi using a wide-field fluorescence microscope (30 s, 405 nm) resulting in immediate colocalization that persisted over time (Figure S8). This clearly shows that pMandi can be used to control protein proximity noninvasively and with high temporal control. To further evaluate the ability of the spatial control of protein proximity by using photoactivation of pMandi, we irradiated a defined field of view with a confocal fluorescence microscope (405 nm). Again, light irradiation resulted in immediate release of Mandi and fluorescence colocalization, indicating protein proximity. Interestingly, the induced fluorescence colocalization was reversed over time, which, as we hypothesize, is due to the diffusion of the active molecule outside of the irradiated area, diluting the concentration of active Mandi (Figure 1D–F). As we expected that short and local irradiation only activates a fraction of the pMandi pool within the field of activation, we speculated that repetitive irradiation with short light pulses can be used to control protein proximity in a reversible manner. Indeed, when we applied successive irradiation, we were able to reinduce protein proximity for at least three cycles in total (Figures 1E and S9). Irradiation of cells in the absence of the photocaged CIP had no effect on protein localization (Figures 1E and S10).

The high membrane permeability of Mandi enables reversible and repeated induction of protein proximity by successive photoactivation of pMandi, but at the same time limits the spatial control of the induction of protein proximity. Single-cell photoactivation with noncovalent CIPs requires an active CIP that is trapped inside the cell after photoactivation and cannot diffuse out of the cell, while being well cell-permeable in its photocaged form. To achieve this, we hypothesized that having an active CIP with a free carboxylic acid moiety that can be photocaged as dimethoxynitrobenzyl ester should fulfill these criteria. A similar strategy has been previously applied for photocaged CIPs based on gibberellic acid^17,18^ and auxin (mammalian cells,^19^ plant cells^20^) as well as for ABA.^16^

**Figure 2:**
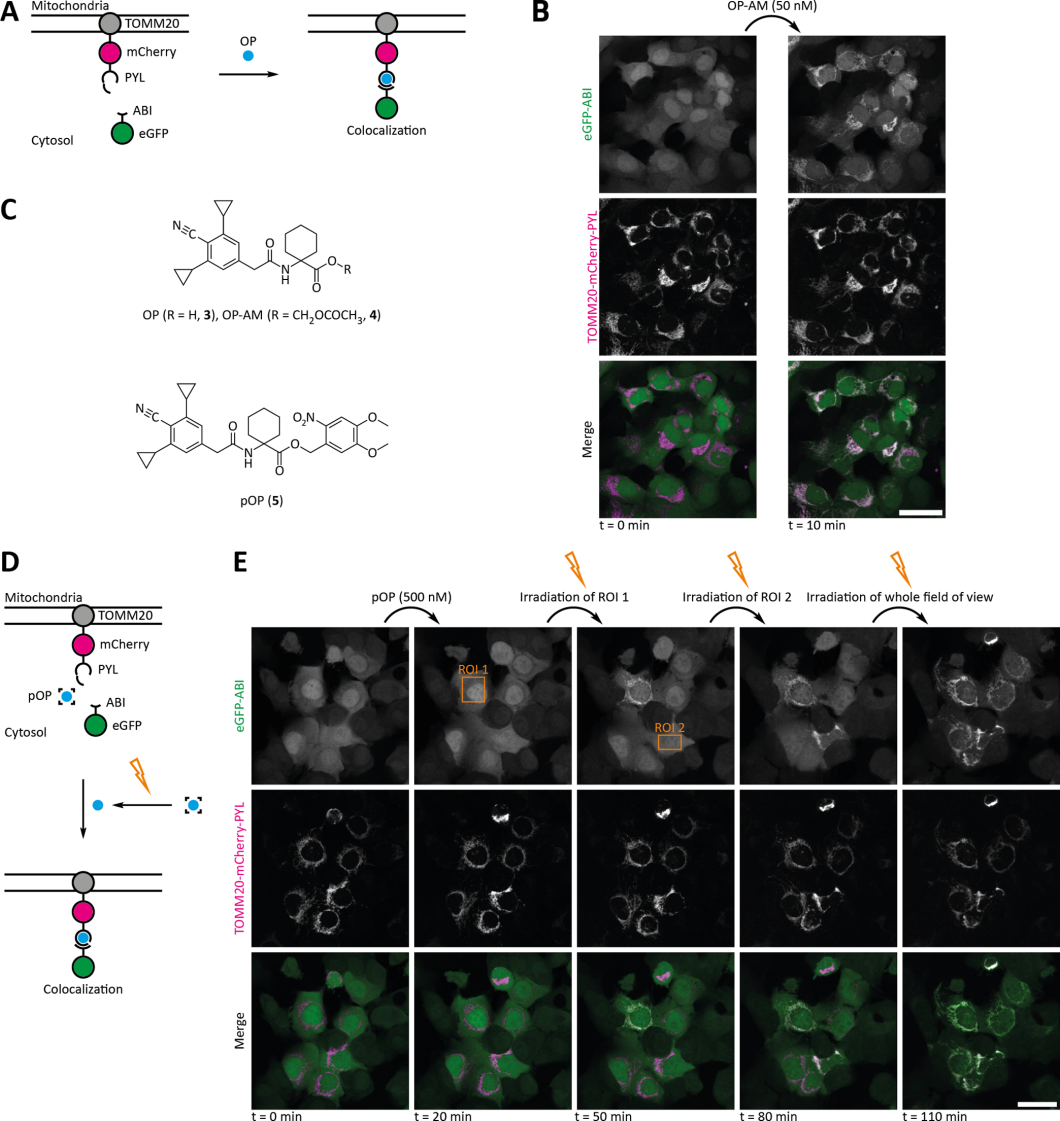
Inducing protein proximity in live cells using opabactin (OP/OP-AM) and photocaged opabactin (pOP). (A) Schematic representation of the used protein constructs and the colocalization assay. (B) OP-AM induces protein colocalization at low nanomolar concentrations in cellulo. Confocal microscopy images of U2OS FlpIN cells stably expressing TOMM20-mCherry-PYL and eGFP-ABI before and 10 min after addition of 50 nM OP-AM. Scale bar: 40 μm. Representative data were obtained from three experiments. (C) Structures of OP/OP-AM and pOP. (D) pOP enables precise local and temporal control over the induction of protein proximity. Colocalization is induced only in the irradiated cells. Schematic representation of the used protein constructs and the colocalization assay. (E) Confocal microscopy images of U2OS FlpIN cells stably expressing TOMM20-mCherry-PYL and eGFP-ABI at different time points before and after addition of 500 nM pOP and after irradiation (405 nm). Irradiation of the indicated area was performed immediately after image acquisition at 20, 50, and 80 min. Scale bar: 40 μm. Representative data were obtained from three experiments.

For the design of a CIP with nanomolar affinity that can be used for photoactivation with spatiotemporal control, we chose the ABA agonist opabactin (OP) that has a 7-fold higher affinity to the ABA receptor complex than ABA itself.^28^ The high affinity enables the use of low concentration, rendering the molecule attractive for use *in vivo*. Moreover, due to the absence of a double bond in OP compared to ABA, no photoinduced isomerization into an inactive isomer can occur.^16,29^ OP has been originally developed for plant research and has not been reported to be used as a CIP so far. We synthesized OP according to the literature^28^ and further derivatized it to the acetoxymethyl ester (OP-AM) and the photocaged dimethoxynitrobenzyl ester (pOP) (Figure 2C; for synthesis description and synthesis scheme, see Section S2.7.5). The protection of the carboxylic acid as acetoxymethyl (AM) ester increases the permeability through the cell membrane and allows the usage of lower working concentration (Figures S11 and S12) as also seen for the AM-protected CIP variants of ABA and gibberellic acid.^5,26^ The AM ester is cleaved intercellularly through endogenous esterases, and the active OP with a free carboxylic acid moiety is released.

First, we tested whether OP can be used as CIP in live mammalian cells. Therefore, we used a colocalization assay analogous to the testing of Mandi/pMandi but with the native ABA receptor PYL instead of PYR^Mandi^ (Figure 2A). OP and OP-AM both induced protein proximity at 10-fold lower concentrations than ABA and ABA-AM, respectively (Figures S13 and S14). The AM-ester-protected compounds allow to use 100-fold lower concentrations than non-AM-protected compounds to efficiently induce protein colocalization. Upon addition of OP-AM protein proximity is induced at a low nanomolar concentration within a few minutes (Figures 2B and S11). Treatment of cells expressing the PYR^Mandi^ receptor protein with OP-AM resulted in the induction of protein proximity only at a micromolar concentration (5 μM, Figure S15). The PYL receptor protein did not show any response to treatment with Mandi at reasonable concentrations (tested up to 50 μM, Figure S16). Hence, the two receptors are orthogonal to each other and can therefore be used in the same sample for targeted protein shuttling.^26^

We also confirmed the orthogonality of OP to PYR^Mandi^/ABI and Mandi to PYL/ABI in a cellular luciferase transcription assay (Figure S17). The luciferase transcription assay also revealed that the difference in permeability between OP or OP-AM becomes negligible at long incubation times (overnight); both compounds showed similar dose–response curves with the PYL/ABI protein pair (Figure S18).

Next, we investigated whether photocaged opabactin (pOP) can be used to spatiotemporally control the induction of protein proximity with light as an external stimulus (Figure 2D). *In vitro* irradiation experiments showed that the photocage can be cleaved with UV and blue light (365 and 405 nm) and the active molecule OP is released in a unimolecular reaction (Figures S19 and S20). We determined the quantum yield of the decay of pOP upon light irradiation at 405 nm to Φ = 0.4% (Figure S21), which is in the typical range for the photolytic cleavage of the dimethoxynitrobenzyl group linked via an ester bond.^30,31^

Similar to pMandi, we characterized the background activity of pOP in a cellular luciferase transcription assay and found that pOP is two orders of magnitude less potent than OP in inducing protein proximity after overnight incubation in the dark (Figure S22). Upon irradiation of cells treated with pOP and successive overnight incubation, we showed that just like pMandi pOP can also be applied to control biological processes in a light dose-dependent manner, in this case luciferase expression (Figure S23).

In the cellular colocalization assay, pOP did not induce protein proximity without irradiation even after 2 h of incubation time (Figure S24). When cells treated with pOP were irradiated with light of 405 nm in a defined field of view using a confocal fluorescence microscope, we observed colocalization that persisted over time (Figure S25). Irradiation in the absence of pOP did not lead to the induction of protein proximity (Figure S25).

Other than for pMandi, the colocalization was stable over time, and no reversion was observed. This indicates that the released OP is much less cell-permeable than Mandi and cannot easily diffuse out of the cell. Irradiation of a region of interest inside a single cell led to the induction of protein proximity only in this irradiated cell, allowing successive photoactivation in several single cells in the same sample (Figures 2E and S26). Thus, pOP enables precise local and temporal control over the induction of protein proximity in live mammalian cells with a single-cell resolution.

We note that the release of the active molecules (Mandi and OP) from their photocaged variants (pMandi and pOP) occurs directly upon light irradiation. Nevertheless, the induction of protein proximity upon photoactivation of pOP takes several minutes (Figures S25 and S26), whereas upon photoactivation of pMandi protein proximity is induced immediately within seconds (Figures 1E,F, S8, and S9). We cannot provide a rational explanation for this observation, but the slower kinetic with pOP does not impair the demonstrated function of the system.

Having successfully shown that both photocaged molecules pMandi and pOP can be used to control protein proximity in live cells, we aimed to show their applicability as photoactivatable CIPs in higher organisms. Both CIPs are attractive for *in vivo* use because they only require low working concentration in cell experiments and are, as plant hormone-based systems, bioorthogonal in vertebrates.

**Figure 3:**
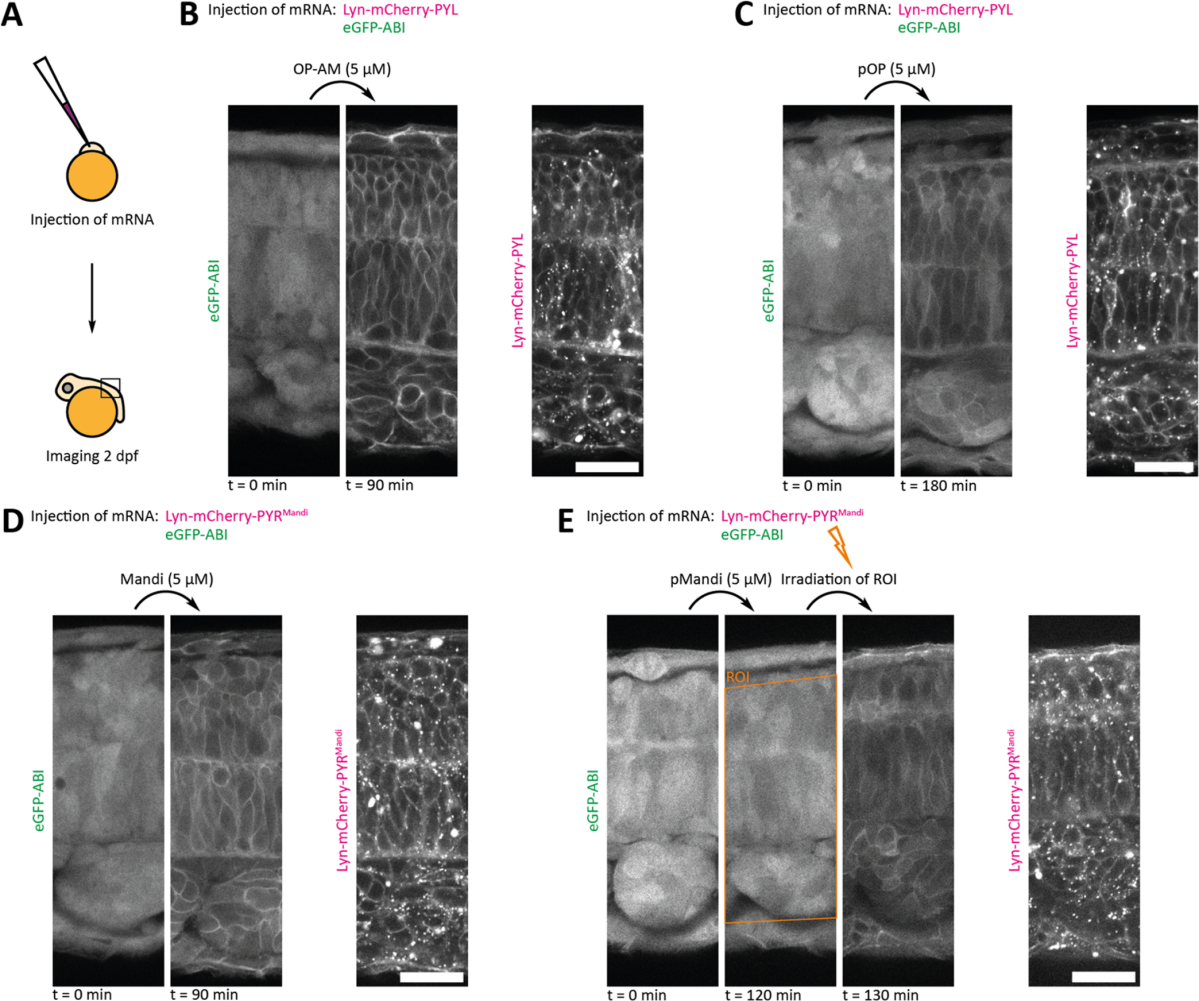
Inducing translocation to the plasma membrane in live medaka embryos using the CIPs OP-AM and Mandi and their photocaged derivatives pOP and pMandi. Confocal microscopy images, scale bars: 20 μm. Selected ROI of the whole field of view depicted, whole field of view, and time courses in Figures S28, S32, S34, and S39. Representative data were obtained from each two to five experiments. (A) Workflow of experiments in medaka embryos. (B) Addition of OP-AM induces protein proximity in eGFP-ABI and Lyn-mCherry-PYL mRNA-injected embryos. Images before and 90 min after addition of 5 μM OP-AM. (C) Addition of pOP induces protein proximity in eGFP-ABI and Lyn-mCherry-PYL mRNA-injected embryos without irradiation. Images before and 180 min after addition of 5 μM pOP. (D) Addition of Mandi induces protein proximity in eGFP-ABI and Lyn-mCherry-PYR^Mandi^ mRNA-injected embryos. Images before and 90 min after addition of 5 μM Mandi. (E) Addition of pMandi induces protein proximity in eGFP-ABI and Lyn-mCherry-PYR^Mandi^ mRNA-injected embryos upon irradiation (405 nm). Images before and 120 min after addition of 5 μM pMandi and 10 min after irradiation. Irradiation of the indicated area was performed immediately after image acquisition at 120 min after addition.

We chose live medaka (*Oryzias latipes*) embryos as model organism and a colocalization assay with fluorescent proteins as readout. As subcellular localization for colocalization, we targeted the plasma membrane. Medaka embryos at the one-cell stage were microinjected with mRNA encoding the fusion proteins Lyn-mCherry-PYR^Mandi^ and eGFP-ABI for experiments with Mandi/pMandi, or Lyn-mCherry-PYL and eGFP-ABI for experiments with OP/OP-AM/pOP (Figure 3A). The experiments in which the respective CIP was added were carried out 2 days post fertilization.

First, the colocalization assay was validated with the nonphotocaged CIPs Mandi and OP-AM. Both compounds achieved efficient protein proximity in all depth of the tissue after addition of the CIP in solution on top of the agarose-embedded embryos at concentrations as low as 500 nM as well as 5 μM within 1–2 h (Figures 3B,D, S27–S29, and S33–S35). At a concentration of 50 nM, no induction of protein proximity was observed upon addition of both OP-AM (Figures S27 and S30) or Mandi (Figures S33 and S36).

Next, to investigate photoactivation *in vivo*, the photocaged CIPs pMandi and pOP were added to the agarose-embedded embryos. Addition of pOP led to an induction of protein proximity without irradiation and is therefore not suitable for photoactivation in live medaka embryos (Figures 3C, S27, and S32). We hypothesize that this is due to cleavage of the ester bond connecting the photocage to the active molecule through another mechanism than light irradiation. The observation that upon addition of OP with the unprotected carboxylic acid moiety no protein proximity is induced (Figures S27 and S31) indicates that the negatively charged carboxylic acid hinders the diffusion inside the medaka embryo. This leads us to the assumption that pOP enters the living organism and cleavage of the ester bond of pOP happens inside the medaka embryo. The development of photocaged variants of OP that are more stable and can be used for photoactivation *in vivo* is the aim of future studies.

Then, we tested the photocaged CIP pMandi for its photoactivation in live medaka embryos. Upon addition of pMandi, we did not observe induction of protein proximity in medaka embryos without irradiation (Figures 3E, S33, and S37). Irradiation of the medaka embryo in the absence of pMandi had no effect on protein localization (Figures S33 and S38). Only after irradiation (405 nm) of medaka embryos treated with pMandi, protein proximity at the plasma membrane was induced within minutes (Figures 3E, S33, and S39). This result demonstrates that pMandi can penetrate well into medaka embryo tissue. Photolytic cleavage of the photocage and thus photoactivation can be obtained inside the live embryo, rapidly inducing protein proximity. Thus, pMandi allows precise temporal control and a noninvasive way to induce protein proximity with light as an external trigger *in vivo*. The linkage of the photocage to an amide nitrogen, as present in pMandi, is apparently stable against hydrolysis *in vivo*. Similar to the experiments in cell culture, we observed that the colocalization previously induced by local irradiation with light decreases over time (Figures S33 and S39). However, this process of reversion is considerably slower *in vivo*.

## Conclusions

In conclusion, our study demonstrates that the photocaged CIP pMandi can effectively initiate protein proximity upon irradiation in both live mammalian cells and live medaka embryos at low working concentrations (500 nM and 5 μM, respectively). Additionally, we introduce a novel and efficient CIP, OP/OP-AM, which can be used at low nanomolar concentration in live mammalian cells, proving to be about an order of magnitude more potent than the commonly used CIP, ABA/ABA-AM. Notably, in live medaka embryos, OP-AM induces protein proximity at a concentration as low as 500 nM. The photocaged derivative, pOP, allows for the use of light as an external stimulus to induce protein proximity in live mammalian cells with single-cell resolution (500 nM). This capability is particularly advantageous for applications requiring the manipulation of individual cells within the same sample. Thus, both photocaged CIPs, pMandi and pOP, offer significant potential for spatiotemporal control of protein proximity in living systems, enabling precise manipulation and investigation of proximity-dependent processes.

## Supporting Information Available

Additional experimental data for *in vitro* irradiation experiments, further microscopy images (whole field of view and time courses), detailed results of the luciferase experiments, as well as additional experimental details, methods, chemical synthesis, and characterization. (PDF)
