# Supplementary material for: Photoactivatable Plant Hormone-Based Chemical Inducers of Proximity for In Vivo Applications
Source: ACS Chem Biol. 2025 Jan 27;20(2):332–9. doi: 10.1021/acschembio.4c00592 (PMC11851429; doi:10.1021/acschembio.4c00592)
Supplement: Supplementary file 1 — cb4c00592_si_001.pdf [file cb4c00592_si_001.pdf]

Supporting Information

Photoactivatable plant hormone-based chemical inducers of proximity for *in vivo* applications

Philipp Pöschko[1,2], Caroline M. Berrou[1], Kaisa Pakari[3,4], Michael J. Ziegler[1], Christoph Kern[1], Birgit Koch[1], Joachim Wittbrodt[3], Richard Wombacher[1,*]

[1] Department of Chemical Biology, Max Planck Institute for Medical Research, Jahnstraße 29, 69120 Heidelberg, Germany

[2] Faculty of Biosciences, Heidelberg University, Im Neuenheimer Feld 234, 69120 Heidelberg, Germany

[3] Centre for Organismal Studies Heidelberg (COS), Heidelberg University, Im Neuenheimer Feld 230, 69120 Heidelberg, Germany

[4] Heidelberg Biosciences International Graduate School (HBIGS), Heidelberg University, Im Neuenheimer Feld 501, 69120 Heidelberg, Germany

Corresponding author: wombacher@mr.mpg.de

Table of Contents

1 Supplementary Figures 3
2 Methods 38
2.1 In vitro irradiation experiments 38
2.2 Cloning and plasmid preparation 40
2.3 Cell culture 45
2.3.1 General conditions 45
2.3.2 Generation of stable cell lines 45
2.3.3 Sample preparation for microscopy 45
2.4 Luciferase transcription assay 46
2.5 Fluorescence microscopy 48
2.5.1 Confocal fluorescence microscopy 48
2.5.2 Widefield fluorescence microscopy 48
2.6 Medaka embryo experiments 49
2.6.1 Fish maintenance 49
2.6.2 In vitro transcription of mRNA for microinjections 49
2.6.3 Microinjections 49
2.6.4 Sample preparation for microscopy 49
2.6.5 Toxicity test of OP-AM, pOP, Mandi and pMandi in medaka embryos 49
2.7 Chemical synthesis and characterization 51
2.7.1 General experimental conditions and analytical methods 51
2.7.2 Purchased reagents ABA and Mandi 53
2.7.3 Synthesis of ABA-AM 54
2.7.4 Synthesis of pMandi 55
2.7.5 Synthesis of OP, pOP and OP-AM 61
2.7.6 NMR spectra 64
3 Author contributions 70
4 Supplementary references 71

1 Supplementary Figures

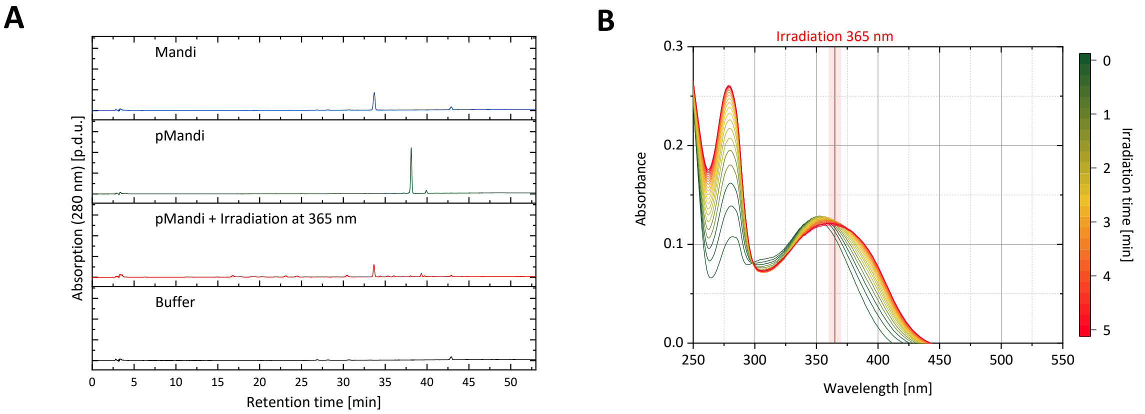

Figure S1: In vitro irradiation of pMandi at 365 nm: pMandi decays to Mandi in a unimolecular reaction. (A) HPLC traces at 280 nm of Mandi, pMandi and buffer without irradiation and of pMandi after irradiation (2 mW, 20 min). (B) Absorption spectra of a solution of pMandi after different irradiation times (2 mW).

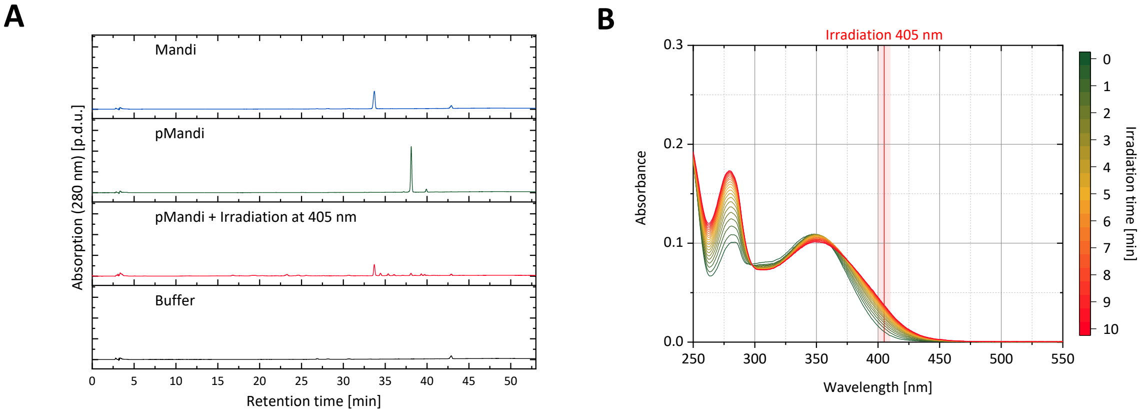

Figure S2: In vitro irradiation of pMandi at 405 nm: pMandi decays to Mandi in a unimolecular reaction. (A) HPLC traces at 280 nm of Mandi, pMandi and buffer without irradiation and of pMandi after irradiation (6 mW, 40 min). (B) Absorption spectra of a solution of pMandi after different irradiation times (6 mW).

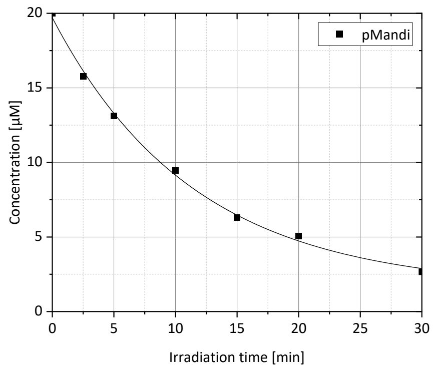

Figure S3: Quantification of the decay of pMandi upon irradiation (405 nm). Solutions of pMandi (initial concentration 20 µM) were irradiated for different times and analyzed via HPLC. The peak area of pMandi (HPLC trace at 280 nm) was measured and normalized by the peak area of pMandi upon HPLC analysis of non-irradiated reference solutions. An exponential decay function was fitted to the data. In relation to the light power hitting the sample (6 mW) the quantum yield of photouncaging was determined to Φ = 2.2 %.

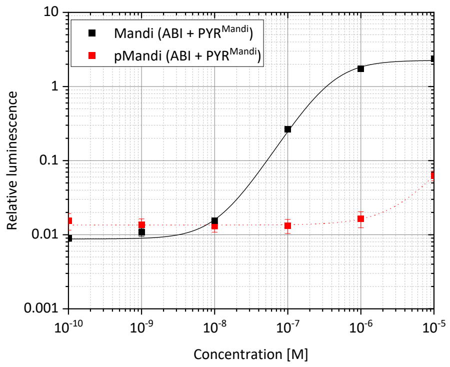

Figure S4: Examination of the background activity of pMandi without irradiation via the dose-response profile for induced luciferase expression with PYR^{Mandi} and ABI for pMandi (in comparison to Mandi). A Hill function was fitted to the data. The background activity of pMandi is three orders of magnitude lower than the activity of Mandi. 293 FlpIN TREx cells expressing an ABI/PYR^{Mandi}-based transcription assay were incubated with different concentrations of either Mandi or pMandi overnight.

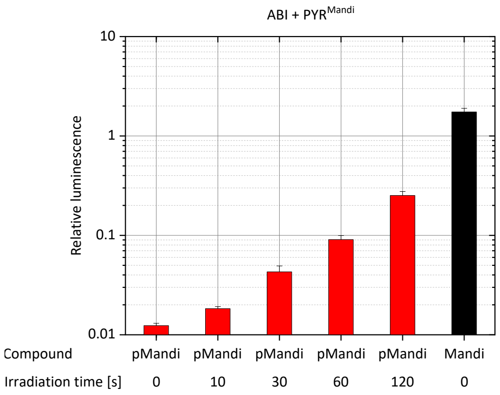

Figure S5: Light-induced luciferase expression with ABI and PYR^{Mandi} in the presence of pMandi upon irradiation (405 nm, 20 mW). The luciferase expression depends on the irradiation time. 293 FlpIN TREx cells expressing an ABI/PYR^{Mandi}-based transcription assay were treated with 1 µM pMandi, subsequently irradiated for different times and incubated overnight. As control, 293 FlpIN TREx cells expressing an ABI/PYR^{Mandi}-based transcription assay were incubated with 1 µM Mandi overnight.

Concentration		500 pM	5 nM	50 nM	500 nM
Compound		Mandi	Mandi	Mandi	Mandi
Receptor protein		PYR^{Mandi}	PYR^{Mandi}	PYR^{Mandi}	PYR^{Mandi}
eGFP channel	Prior addition	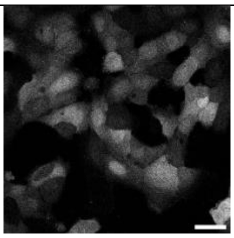	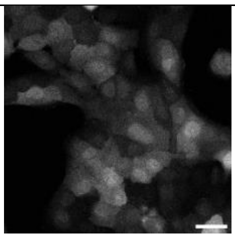	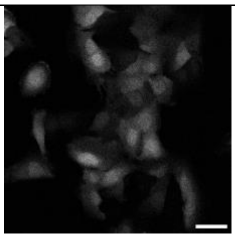	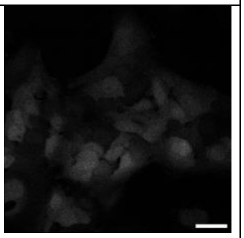
	5 min post addition	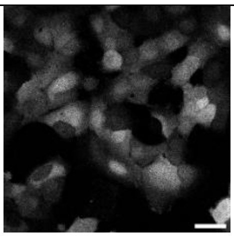	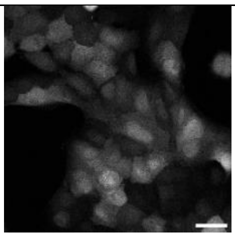	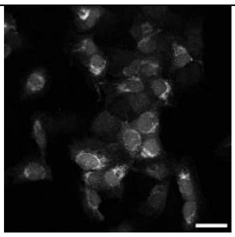	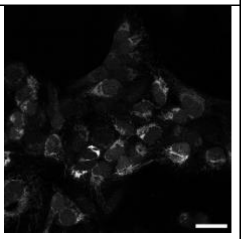
	10 min post addition	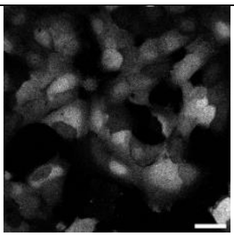	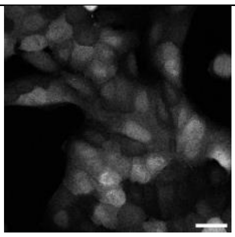	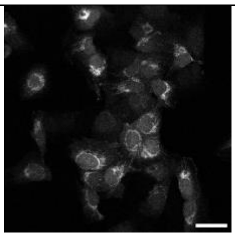	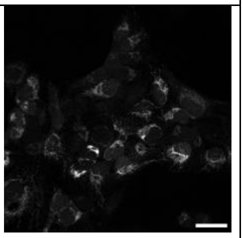
	20 min post addition	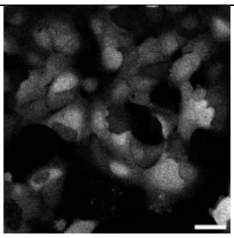	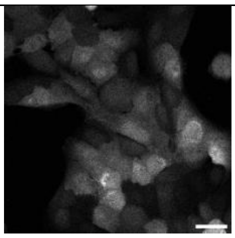	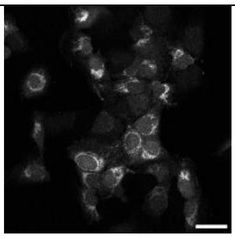	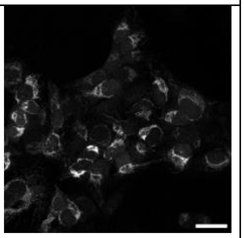
	30 min post addition	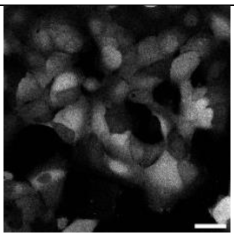	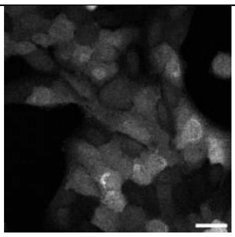	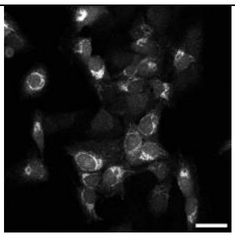	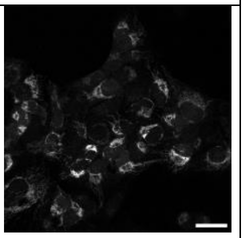
mCherry channel	30 min post addition	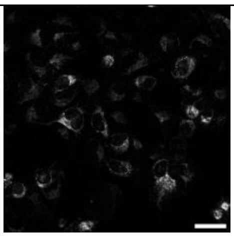	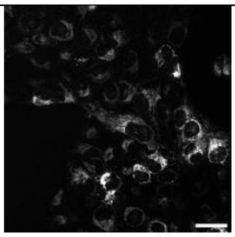	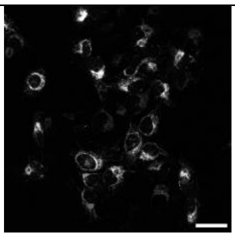	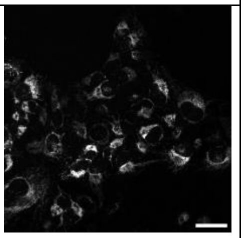

Figure S6: Titration of Mandi in cellulo. Confocal microscopy images of U2OS FlpIN cells stably expressing TOMM20-mCherry-PYR^{Mandi} and eGFP-ABI at different timepoints before and after addition of varying concentrations of Mandi. Scale bars at 40 µm.

Concentration		500 nM
Compound		pMandi
Receptor protein		PYR^{Mandi}
eGFP channel	Prior addition	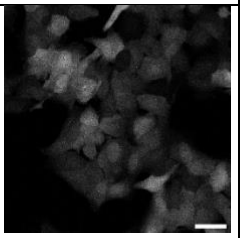
	120 min post addition	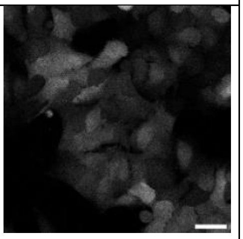
mCherry channel	120 min post addition	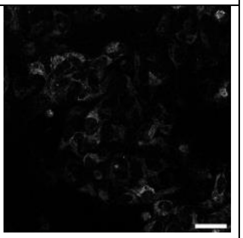

Figure S7: Addition of the photocaged molecule pMandi does not induce protein proximity without irradiation. Confocal microscopy images of U2OS FlpIN cells stably expressing TOMM20-mCherry-PYR^{Mandi} and eGFP-ABI before and 120 min post addition of 500 nM pMandi. Scale bars at 40 µm.

Concentration		500 nM	
Compound		pMandi	DMSO
Receptor protein		PYR^{Mandi}	PYR^{Mandi}
eGFP channel	Prior addition	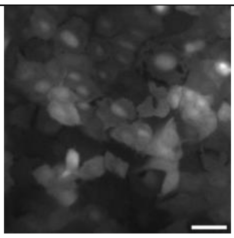	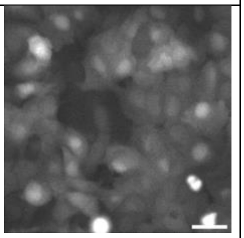
	20 min post addition	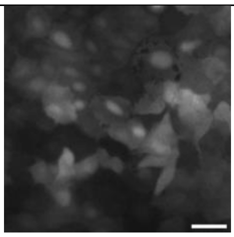	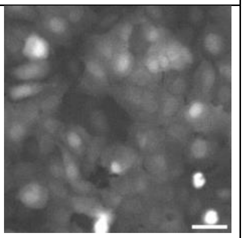
	1 min post irradiation	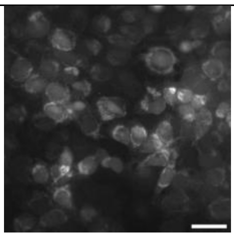	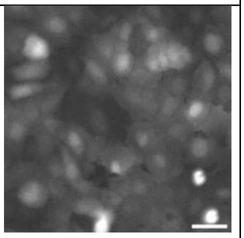
	5 min post irradiation	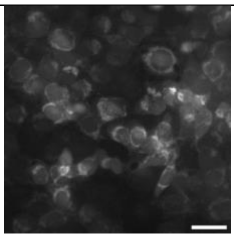	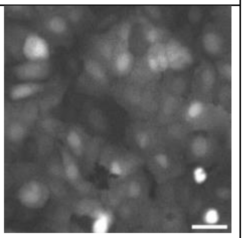
	20 min post irradiation	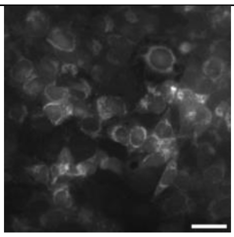	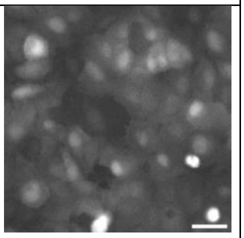
mCherry channel	20 min post irradiation	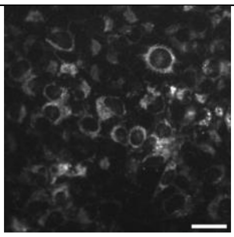	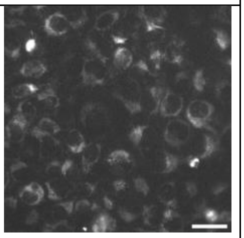

Figure S8: Photouncaging of pMandi with high light dose (405 nm, 30 s) on a widefield microscope induces protein proximity in cellulo without reversion over time. In cells not treated with pMandi no protein proximity is induced upon irradiation (DMSO control). Irradiation was performed immediately after image acquisition at 20 min post addition. Widefield microscopy images of U2OS FlpIN cells stably expressing TOMM20-mCherry-PYR^{Mandi} and eGFP-ABI at before and after addition of 500 nM pMandi or 0.5 % DMSO and at different timepoints after irradiation. Scale bars at 40 µm.

Concentration	500 nM			
Compound	pMandi			
Receptor protein	PYR^{Mandi}			
Prior addition	20 min post addition	1 min post irradiation 1	5 min post irradiation 1	10 min post irradiation 1
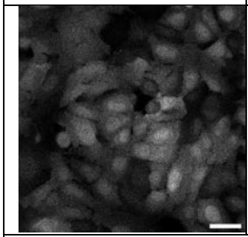	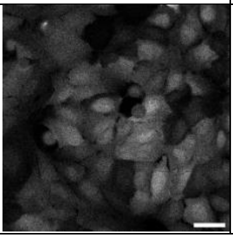	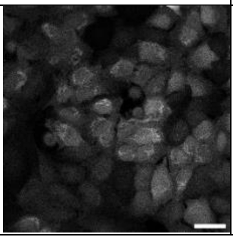	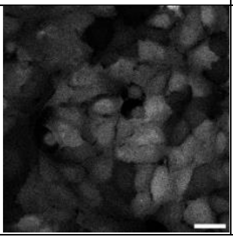	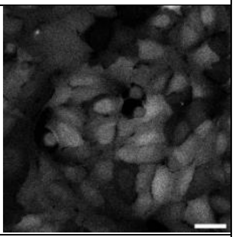
eGFP channel				
15 min post irradiation 1	20 min post irradiation 1	1 min post irradiation 2	5 min post irradiation 2	10 min post irradiation 2
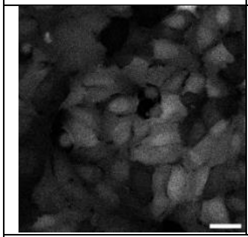	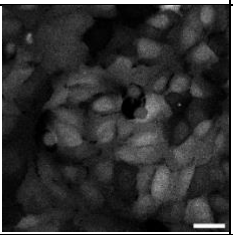	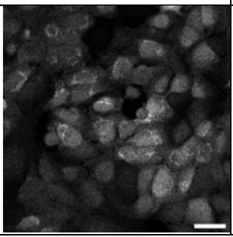	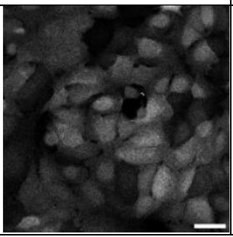	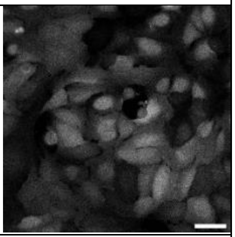
eGFP channel				
15 min post irradiation 2	20 min post irradiation 2	1 min post irradiation 3	5 min post irradiation 3	10 min post irradiation 3
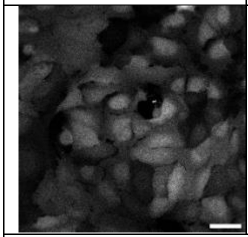	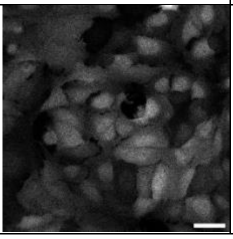	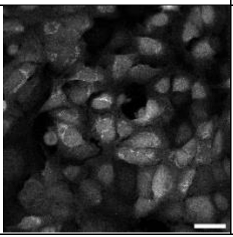	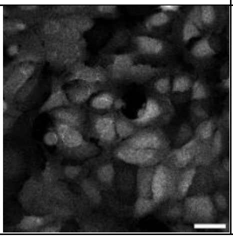	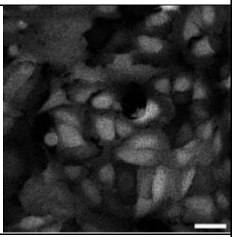
eGFP channel				
15 min post irradiation 3	20 min post irradiation 3			20 min post irradiation 3
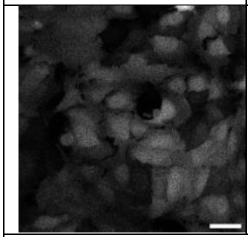	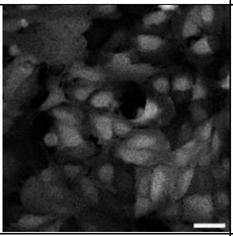			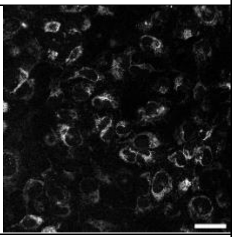
eGFP channel				mCherry channel

Figure S9: Time course of irradiation experiment of cells treated with pMandi at the confocal microscope with three successive irradiations (405 nm) of the whole field of view each 20 min. First irradiation was performed immediately after image acquisition at 20 min post addition. Protein proximity is induced upon each irradiation repeatedly and reverses over time. Confocal microscopy images of U2OS FlpIN cells stably expressing TOMM20-mCherry-PYR^{Mandi} and eGFP-ABI before and after addition of 500 nM pMandi, and at different timepoints after the irradiations. Scale bars at 40 µm, whole field of view depicted. Representative data of four experiments.

Concentration				
Compound	DMSO			
Receptor protein	PYR^{Mandi}			
Prior addition	20 min post addition	1 min post irradiation 1	5 min post irradiation 1	10 min post irradiation 1
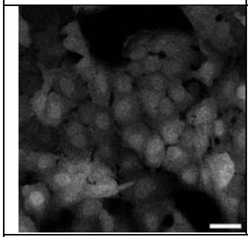	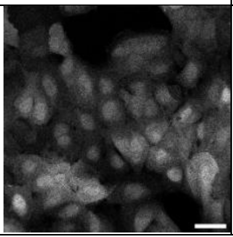	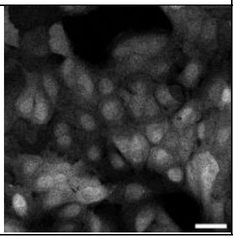	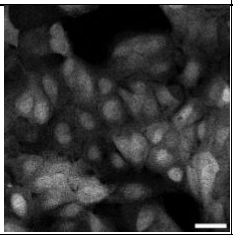	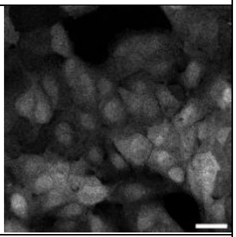
eGFP channel				
15 min post irradiation 1	20 min post irradiation 1	1 min post irradiation 2	5 min post irradiation 2	10 min post irradiation 2
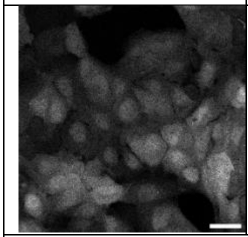	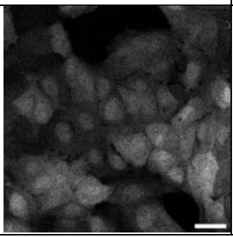	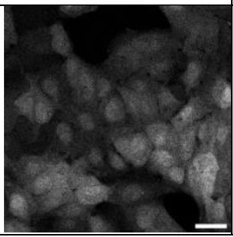	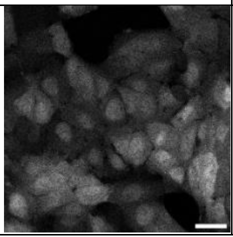	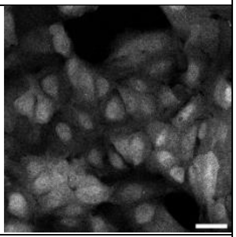
eGFP channel				
15 min post irradiation 2	20 min post irradiation 2	1 min post irradiation 3	5 min post irradiation 3	10 min post irradiation 3
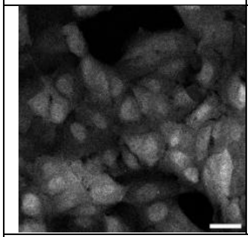	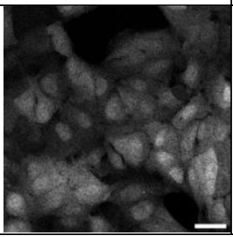	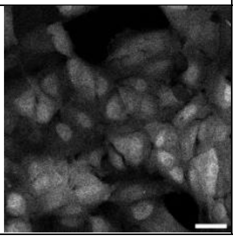	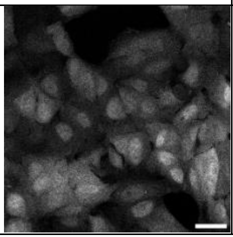	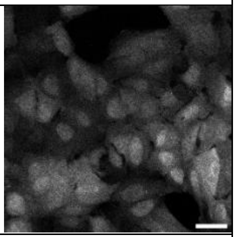
eGFP channel				
15 min post irradiation 3	20 min post irradiation 3			20 min post irradiation 3
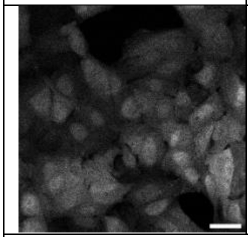	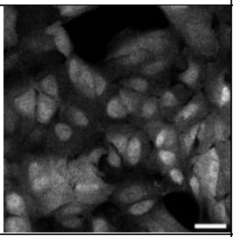			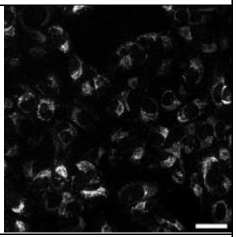
eGFP channel				mCherry channel

Figure S10: Time course of irradiation experiment of cells treated with DMSO at the confocal microscope with three successive irradiations (405 nm) of the whole field of view each 20 min (DMSO control). First irradiation was performed immediately after image acquisition at 20 min post addition. Repeated irradiation of cells at the confocal microscope does not induce protein proximity in the absence of pMandi. Three successive irradiations (405 nm) of the whole field of view each 20 min. Confocal microscopy images of U2OS FlpIN cells stably expressing TOMM20-mCherry-PYR^{Mandi} and eGFP-ABI before and after addition of 0.5 % DMSO, and at different timepoints after the irradiations. Scale bars at 40 µm, whole field of view depicted. Representative data of four experiments.

Concentration		500 pM	5 nM	50 nM	500 nM
Compound		OP-AM	OP-AM	OP-AM	OP-AM
Receptor protein		PYL	PYL	PYL	PYL
eGFP channel	Prior addition	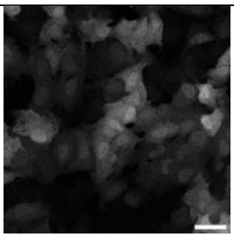	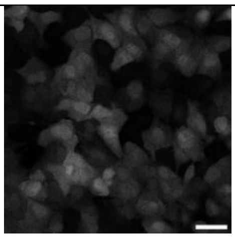	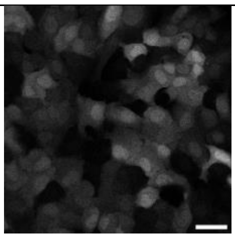	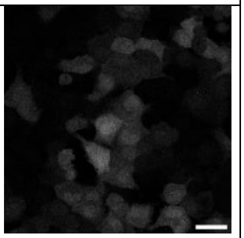
	5 min post addition	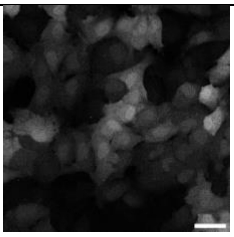	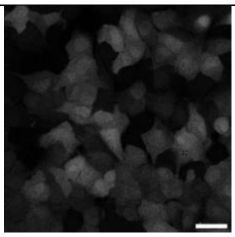	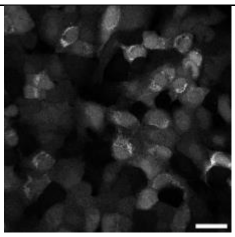	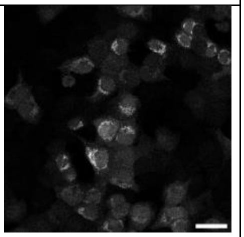
	10 min post addition	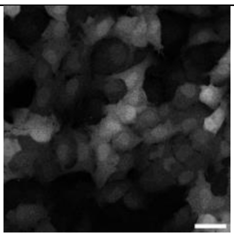	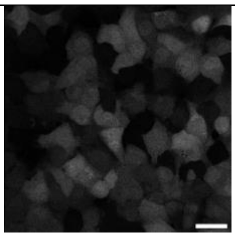	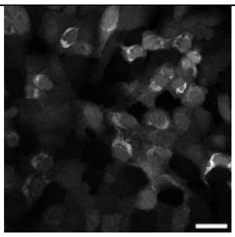	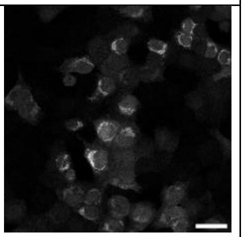
	20 min post addition	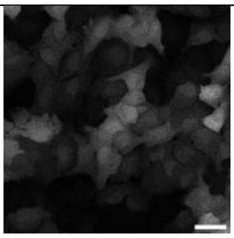	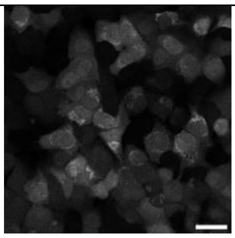	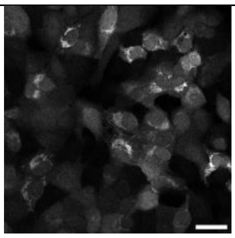	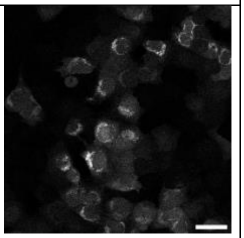
	30 min post addition	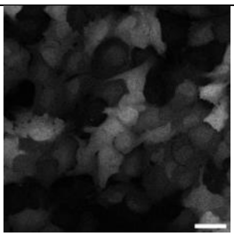	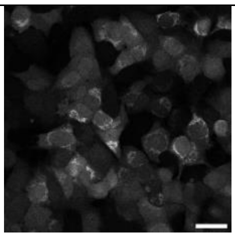	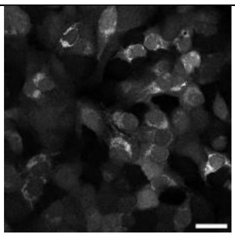	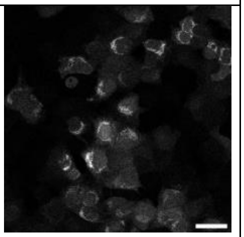
mCherry channel	30 min post addition	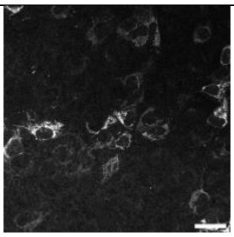	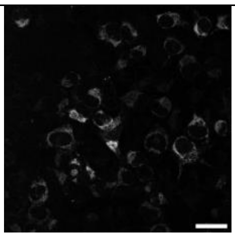	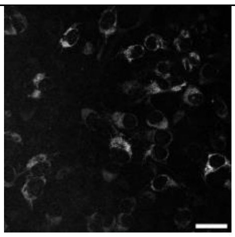	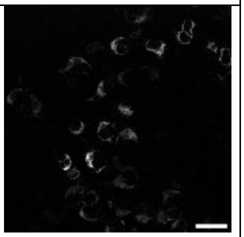

Figure S11: Titration of OP-AM in cellulo. Confocal microscopy images of U2OS FlpIN cells stably expressing TOMM20-mCherry-PYL and eGFP-ABI at different timepoints before and after addition of varying concentrations of OP-AM. Scale bars at 40 µm.

Concentration		50 nM	500 nM	5 µM
Compound		OP	OP	OP
Receptor protein		PYL	PYL	PYL
eGFP channel	Prior addition	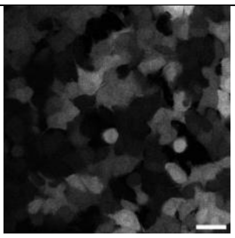	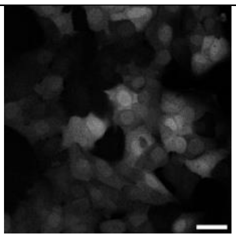	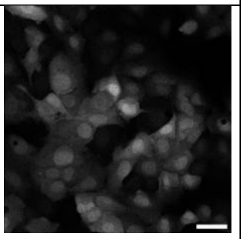
	5 min post addition	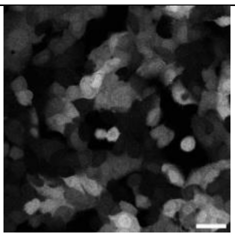	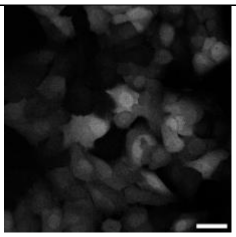	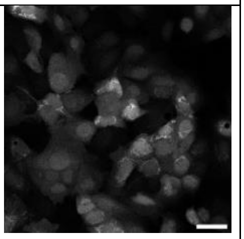
	10 min post addition	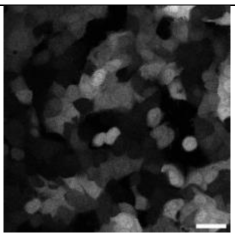	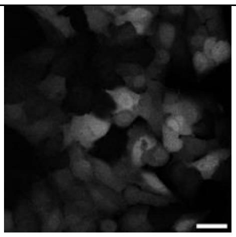	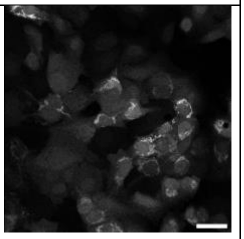
	20 min post addition	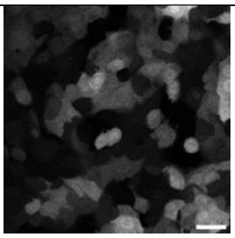	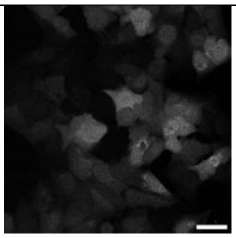	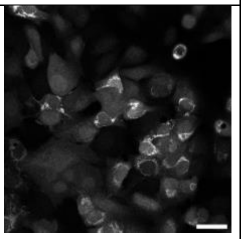
	30 min post addition	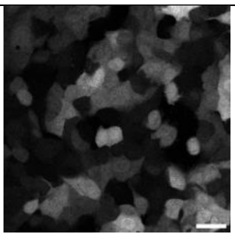	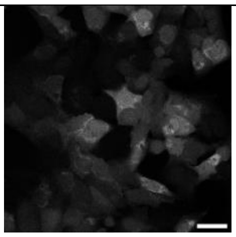	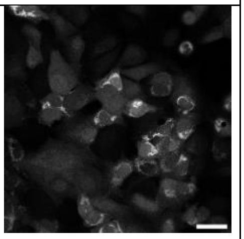
mCherry channel	30 min post addition	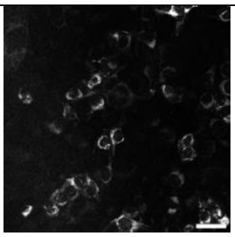	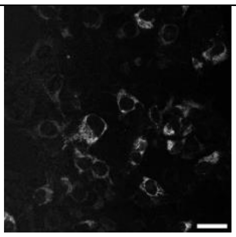	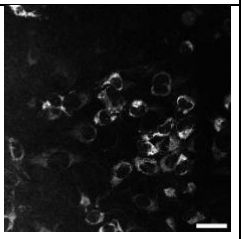

Figure S12: Titration of OP in cellulo. Confocal microscopy images of U2OS FlpIN cells stably expressing TOMM20-mCherry-PYL and eGFP-ABI at different timepoints before and after addition of varying concentrations of OP. Scale bars at 40 µm.

Concentration		5 nM	50 nM	500 nM
Compound		ABA-AM	ABA-AM	ABA-AM
Receptor protein		PYL	PYL	PYL
eGFP channel	Prior addition	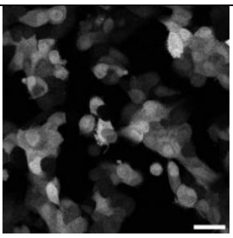	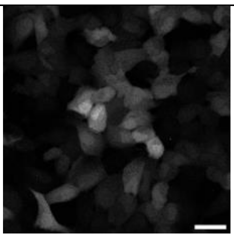	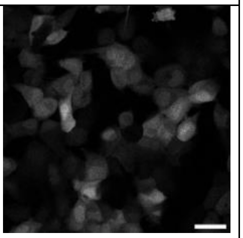
	5 min post addition	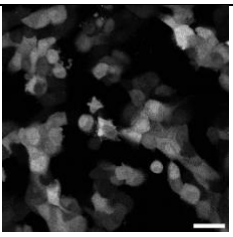	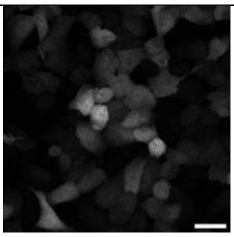	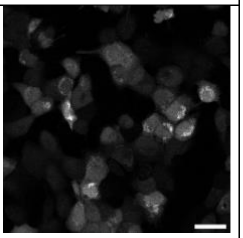
	10 min post addition	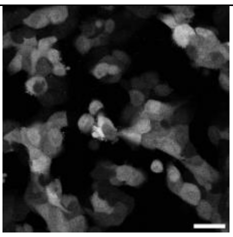	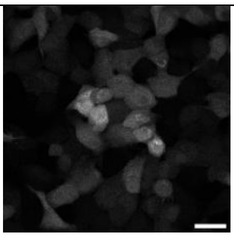	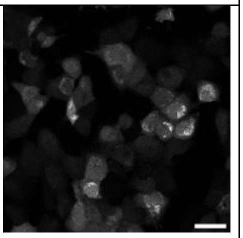
	20 min post addition	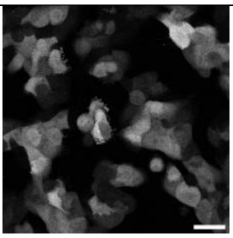	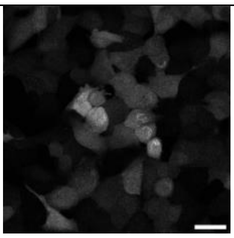	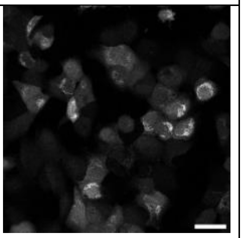
	30 min post addition	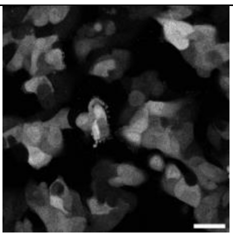	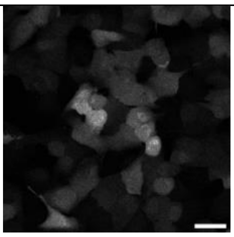	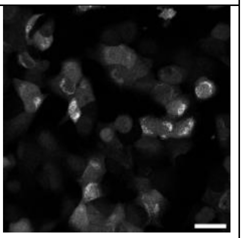
mCherry channel	30 min post addition	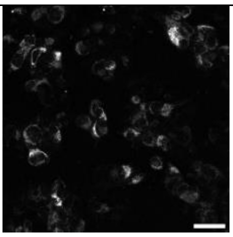	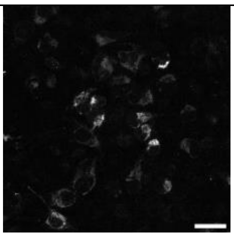	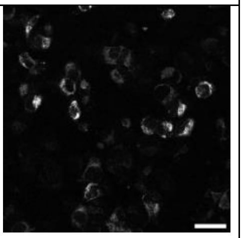

Figure S13: Titration of abscisic acid acetoxymethyl ester (ABA-AM) in cellulo. Confocal microscopy images of U2OS FlpIN cells stably expressing TOMM20-mCherry-PYL and eGFP-ABI at different timepoints before and after addition of varying concentrations of ABA-AM. Scale bars at 40 µm.

Concentration		500 nM	5 µM	50 µM
Compound		ABA	ABA	ABA
Receptor protein		PYL	PYL	PYL
eGFP channel	Prior addition	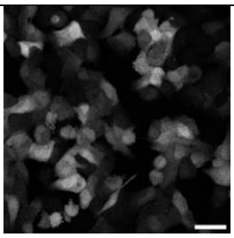	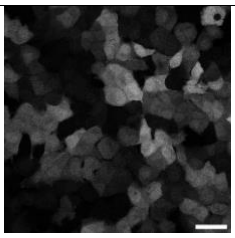	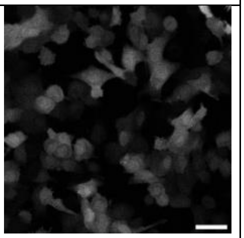
	5 min post addition	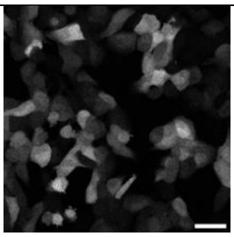	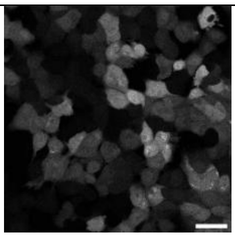	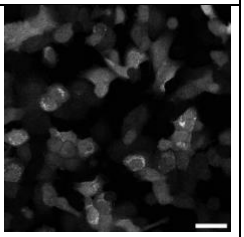
	10 min post addition	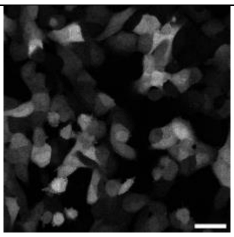	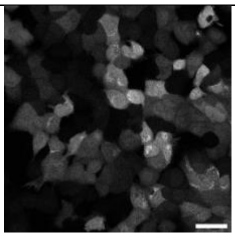	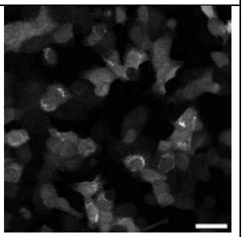
	20 min post addition	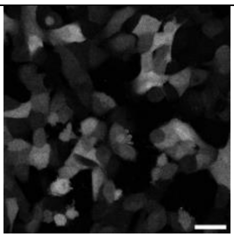	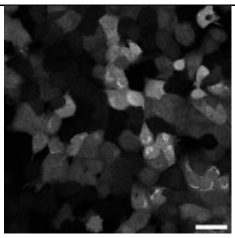	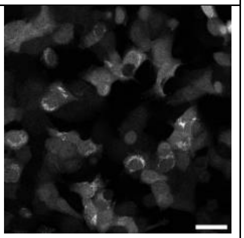
	30 min post addition	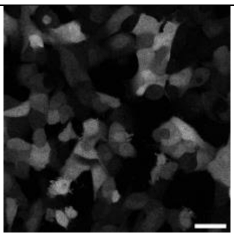	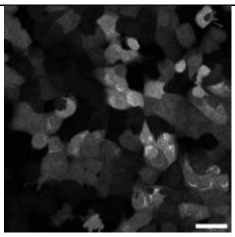	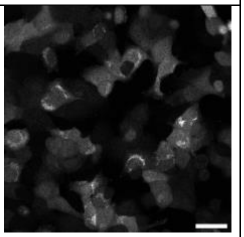
mCherry channel	30 min post addition	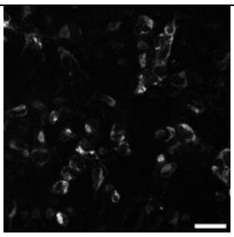	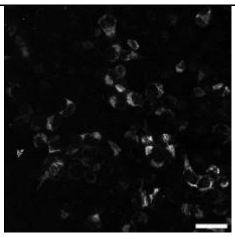	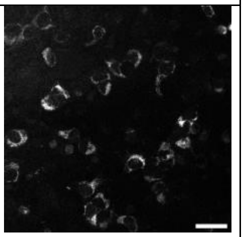

Figure S14: Titration of abscisic acid (ABA) in cellulo. Confocal microscopy images of U2OS FlpIN cells stably expressing TOMM20-mCherry-PYL and eGFP-ABI at different timepoints before and after addition of varying concentrations of ABA. Scale bars at 40 µm.

Concentration		50 nM	500 nM	5 µM
Compound		OP-AM	OP-AM	OP-AM
Receptor protein		PYR^{Mandi}	PYR^{Mandi}	PYR^{Mandi}
eGFP channel	Prior addition	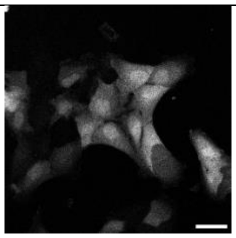	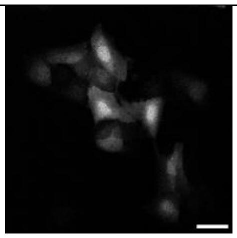	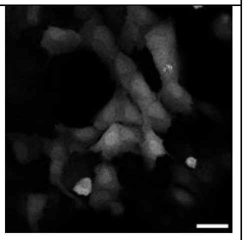
	5 min post addition	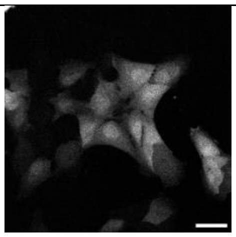	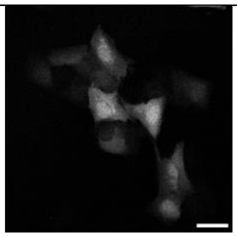	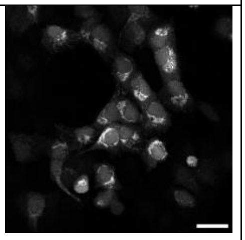
	10 min post addition	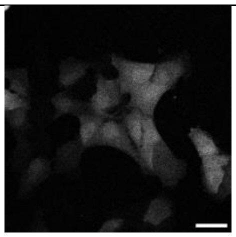	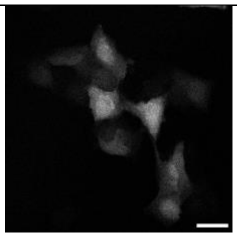	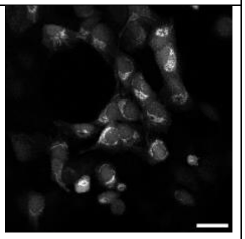
	15 min post addition	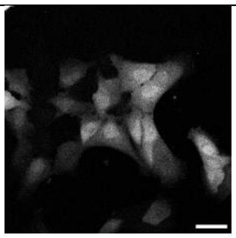	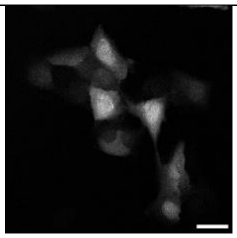	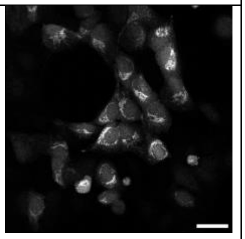
	20 min post addition	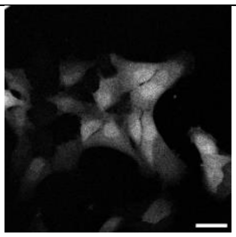	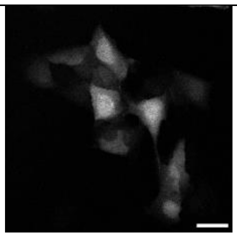	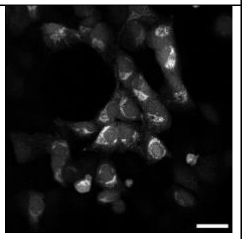
mCherry channel	20 min post addition	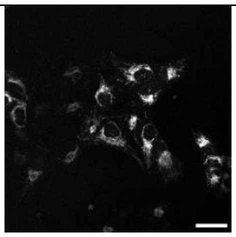	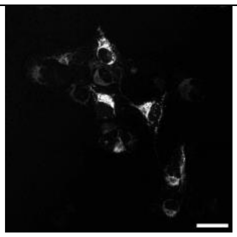	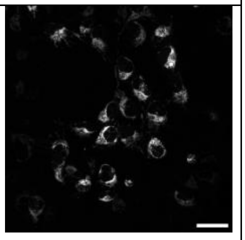

Figure S15: Titration of OP-AM on cells expressing the Mandi receptor PYR^{Mandi}: The Mandi receptor PYR^{Mandi} responds to OP-AM treatment only at micromolar concentration. Confocal microscopy images of U2OS FlpIN cells stably expressing TOMM20-mCherry-PYR^{Mandi} and eGFP-ABI at different timepoints before and after addition of varying concentrations of OP-AM. Scale bars at 40 µm.

Concentration		50 µM
Compound		Mandi
Receptor protein		PYL
eGFP channel	Prior addition	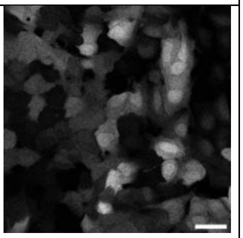
	5 min post addition	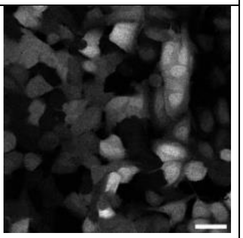
	10 min post addition	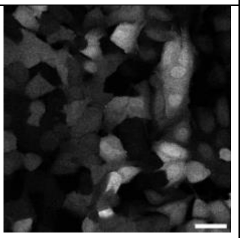
	20 min post addition	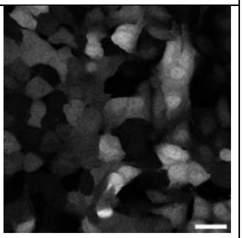
	30 min post addition	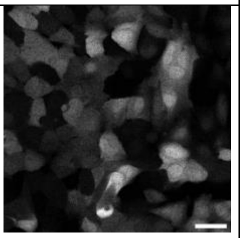
mCherry channel	30 min post addition	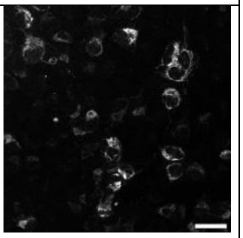

Figure S16: Addition of Mandi to cells expressing the abscisic acid (ABA) / OP receptor PYL does not induce protein proximity. Confocal microscopy images of U2OS FlpIN cells stably expressing TOMM20-mCherry-PYL and eGFP-ABI at different timepoints before and after addition of 50 µM Mandi. Scale bars at 40 µm.

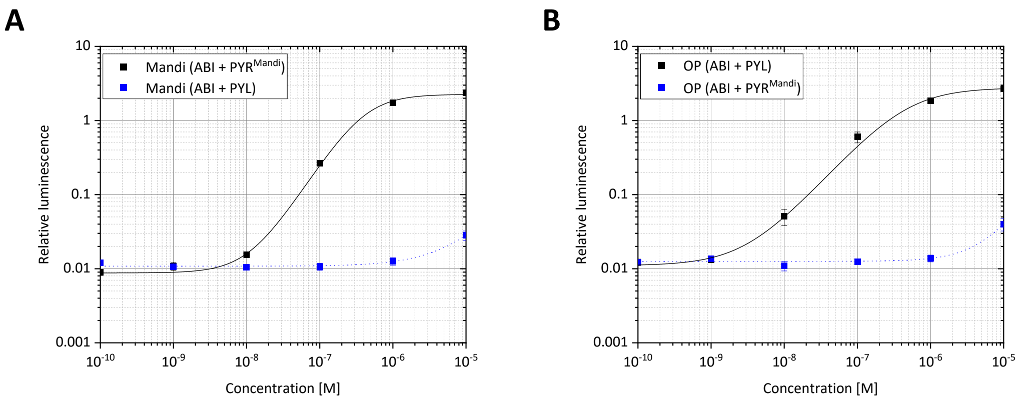

Figure S17: Examination of the orthogonality of Mandi and OP via the dose-response profile for induced luciferase expression with each PYR^{Mandi} or PYL and ABI. A Hill function was fitted to the data. Mandi (A) and OP (B) are both three orders of magnitude more potent in inducing luciferase expressing in combination with their respective receptor protein (PYR^{Mandi} for Mandi and PYL for OP) compared to the non-target receptor protein (PYL for Mandi, PYR^{Mandi} for OP). 293 FlpIN TREx cells expressing an ABI/PYR^{Mandi} or ABI/PYL-based transcription assay were incubated with different concentrations of Mandi or OP overnight.

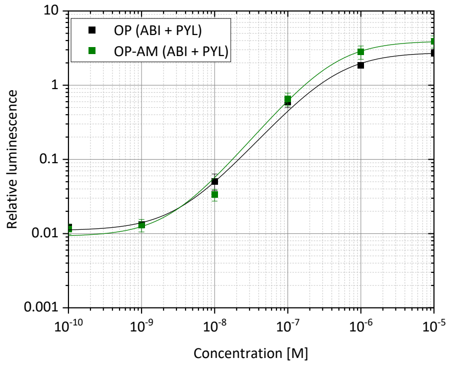

Figure S18: Comparison of OP and OP-AM regarding their dose-response profile for induced luciferase expression with PYL and ABI. A Hill function was fitted to the data. Both compounds achieve similar dose-response profiles. 293 FlpIN TREx cells expressing an ABI/PYL-based transcription assay were incubated with different concentrations of OP or OP-AM overnight.

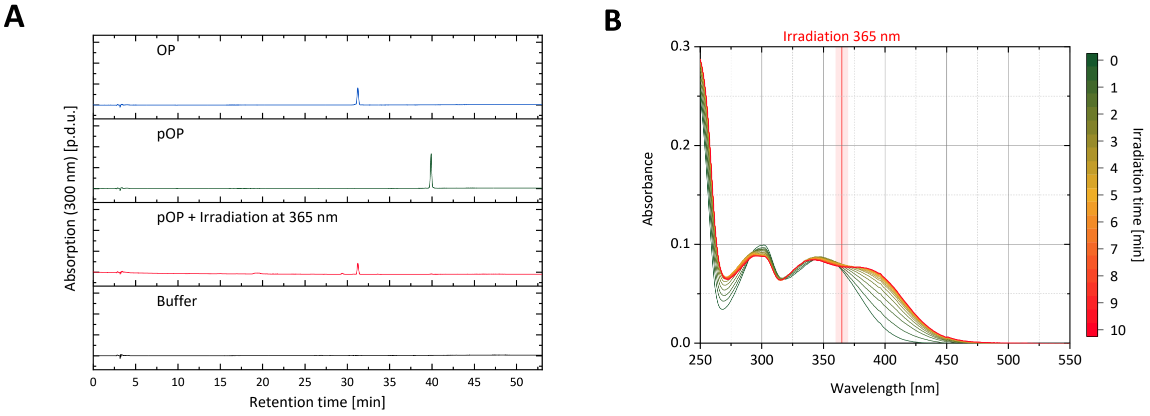

Figure S19: In vitro irradiation of pOP at 365 nm: pOP decays to OP in a unimolecular reaction. (A) HPLC traces at 300 nm of OP, pOP and buffer without irradiation and of pOP after irradiation (20 mW, 10 min). (B) Absorption spectra of a solution of pOP after different irradiation times (20 mW).

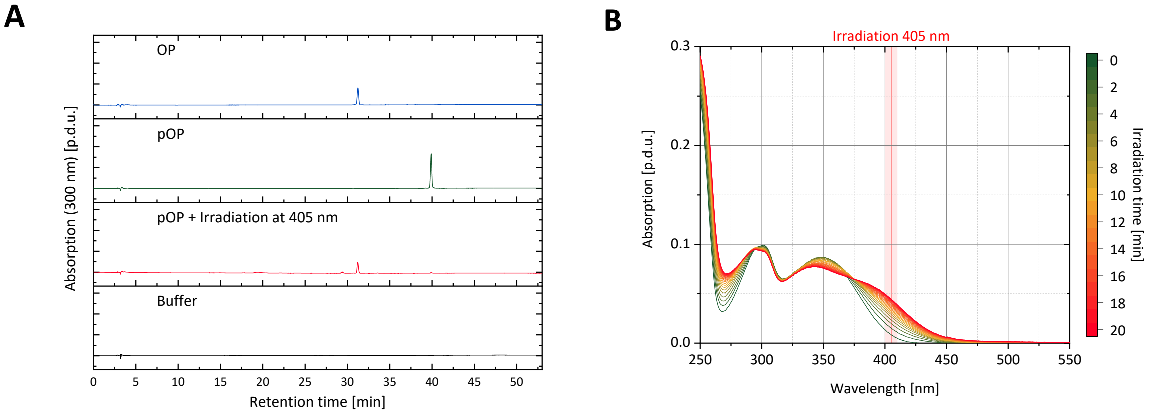

Figure S20: In vitro irradiation of pOP at 405 nm: pOP decays to OP in a unimolecular reaction. (A) HPLC traces at 300 nm of OP, pOP and buffer without irradiation and of pOP after irradiation (70 mW, 40 min). (B) Absorption spectra of a solution of pOP after different irradiation times (70 mW).

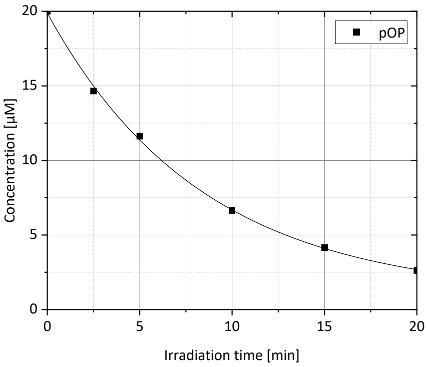

Figure S21: Quantification of the decay of pOP upon irradiation (405 nm). Solutions of pOP (initial concentration 20 µM) were irradiated for different times and analyzed via HPLC. The peak area of pOP (HPLC trace at 300 nm) was measured and normalized by the peak area of pOP upon HPLC analysis of a non-irradiated reference solution. An exponential decay function was fitted to the data. In relation to the light power hitting the sample (70 mW) the quantum yield of photouncaging was determined to $\Phi = 0.4$ %.

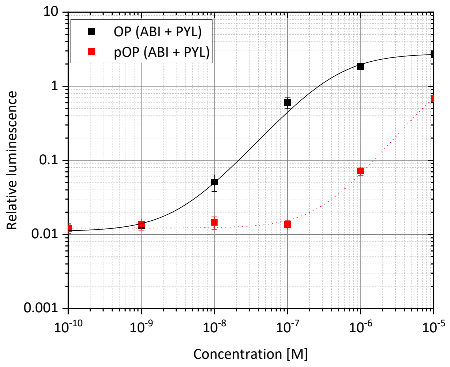

Figure S22: Examination of the background activity of pOP without irradiation via the dose-response profile for induced luciferase expression with PYL and ABI for pOP (in comparison to OP). A Hill function was fitted to the data. The background activity of pOP is two orders of magnitude lower than the activity of OP. 293 FlpIN TREx cells expressing an ABI/PYL-based transcription assay were incubated with different concentrations of either OP or pOP overnight.

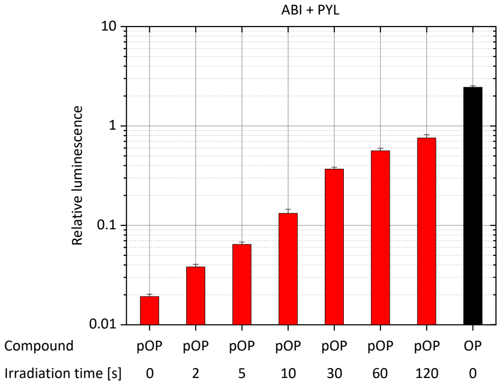

Figure S23: Light-induced luciferase expression with ABI and PYL in the presence of pOP upon irradiation (405 nm, 217 mW). The luciferase expression depends on the irradiation time. 293 FlpIN TREx cells expressing an ABI/PYL-based transcription assay were treated with 1 µM pOP, subsequently irradiated for different times and incubated overnight. As control, 293 FlpIN TREx cells expressing an ABI/PYL-based transcription assay were incubated with 1 µM OP overnight.

<table>
<tr><td colspan="2">Concentration</td><td>500 nM</td></tr>
<tr><td colspan="2">Compound</td><td>pOP</td></tr>
<tr><td colspan="2">Receptor protein</td><td>PYL</td></tr>
<tr><td rowspan="2">eGFP channel</td><td>Prior addition</td><td>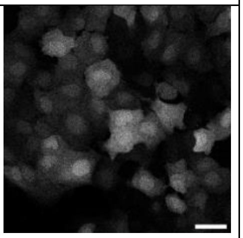</td></tr>
<tr><td>120 min post addition</td><td>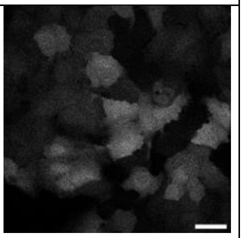</td></tr>
<tr><td>mCherry channel</td><td>120 min post addition</td><td>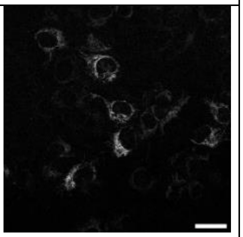</td></tr>
</table>

Figure S24: Addition of the photocaged molecule OP does not induce protein proximity without irradiation. Confocal microscopy images of U2OS FlpIN cells stably expressing TOMM20-mCherry-PYL and eGFP-ABI before and 120 min post addition of 500 nM pOP. Scale bars at 40 µm.

Concentration		500 nM	
Compound		pOP	DMSO
Receptor protein		PYL	PYL
eGFP channel	Prior addition	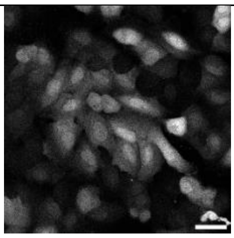	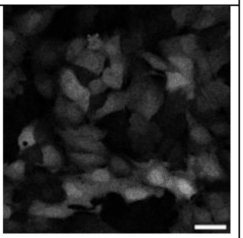
	20 min post addition	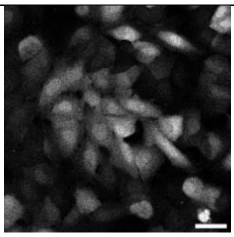	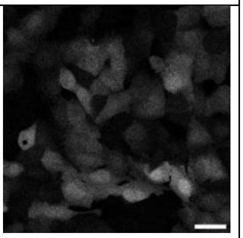
	1 min post irradiation	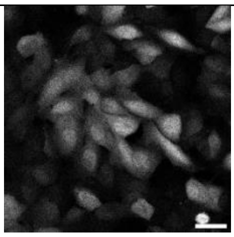	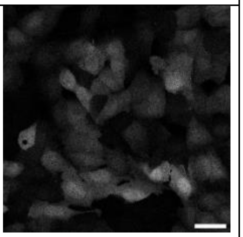
	5 min post irradiation	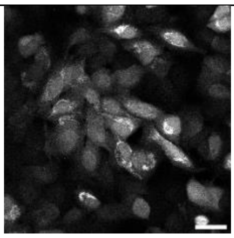	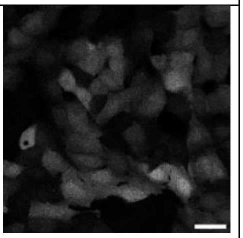
	20 min post irradiation	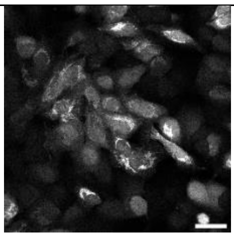	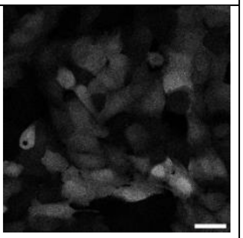
mCherry channel	20 min post irradiation	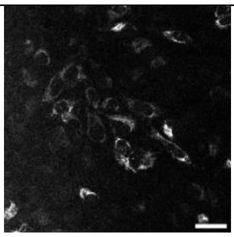	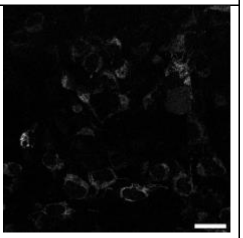

Figure S25: Single irradiation (405 nm) of the whole field of view of cells treated with pOP at the confocal microscope induces permanent protein proximity. In cells not treated with pOP no protein proximity is induced upon irradiation (DMSO control). Irradiation was performed immediately ***after*** image acquisition at 20 min post addition. Confocal microscopy images of U2OS FlpIN cells stably expressing TOMM20-mCherry-PYL and eGFP-ABI before and after addition of 500 nM pOP or 0.5 % DMSO, and at different timepoints after the irradiation of the whole field of view. Scale bars at 40 µm, whole field of view depicted.

Concentration	500 nM			
Compound	pOP			
Receptor protein	PYL			
Prior addition	20 min post addition	1 min post irradiation 1	5 min post irradiation 1	10 min post irradiation 1
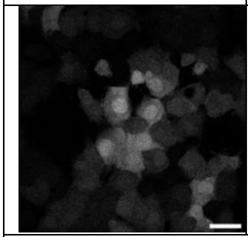	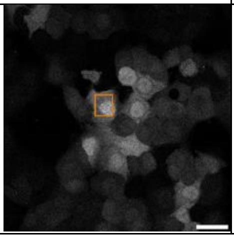	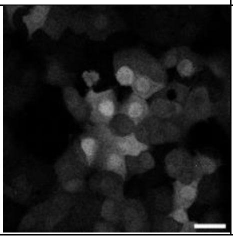	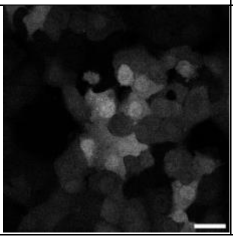	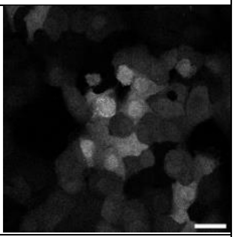
eGFP channel				
15 min post irradiation 1	20 min post irradiation 1	30 min post irradiation 1	1 min post irradiation 2	5 min post irradiation 2
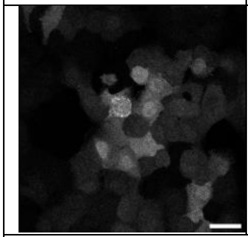	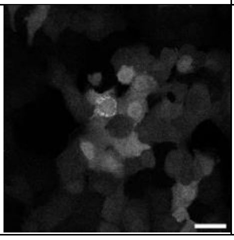	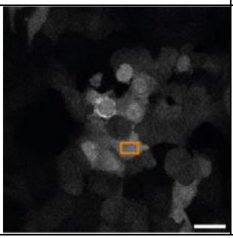	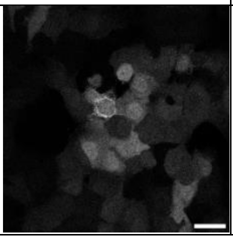	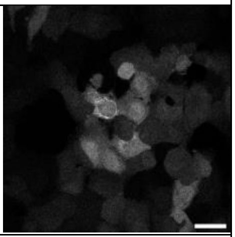
eGFP channel				
10 min post irradiation 2	15 min post irradiation 2	20 min post irradiation 2	30 min post irradiation 2	1 min post irradiation 3
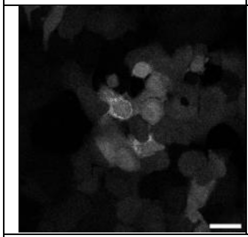	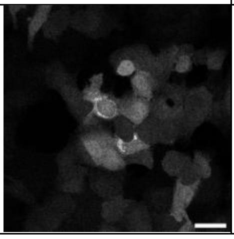	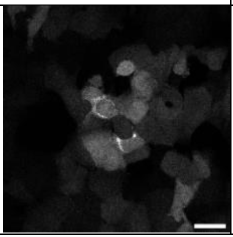	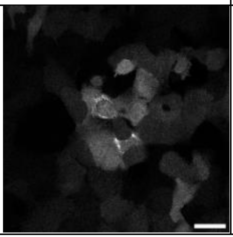	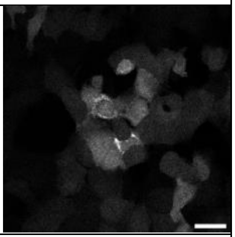
eGFP channel				
5 min post irradiation 3	10 min post irradiation 3	20 min post irradiation 3	30 min post irradiation 3	30 min post irradiation 3
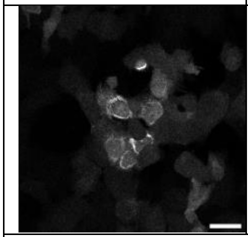	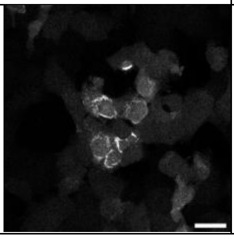	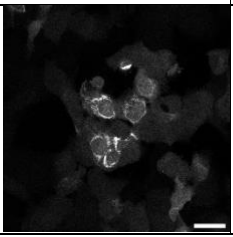	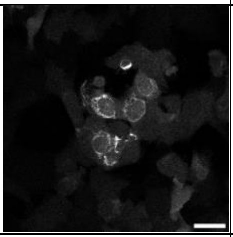	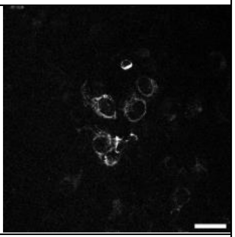
eGFP channel				mCherry channel

Figure S26: Time course of irradiation experiment of cells treated with pOP at the confocal microscope. Three successive irradiations (405 nm) in a 30 min interval: Irradiation 1 and 2: single cells (area outlined in orange), Irradiation 3: whole field of view. First irradiation was performed immediately after image acquisition at 20 min post addition. Permanent protein proximity is only induced in the irradiated cells. Confocal microscopy images of U2OS FlpIN cells stably expressing TOMM20-mCherry-PYL and eGFP-ABI before and after addition of 500 nM pOP, and at different timepoints after the irradiations. Scale bars at 40 µm, whole field of view depicted. Representative data of three experiments.

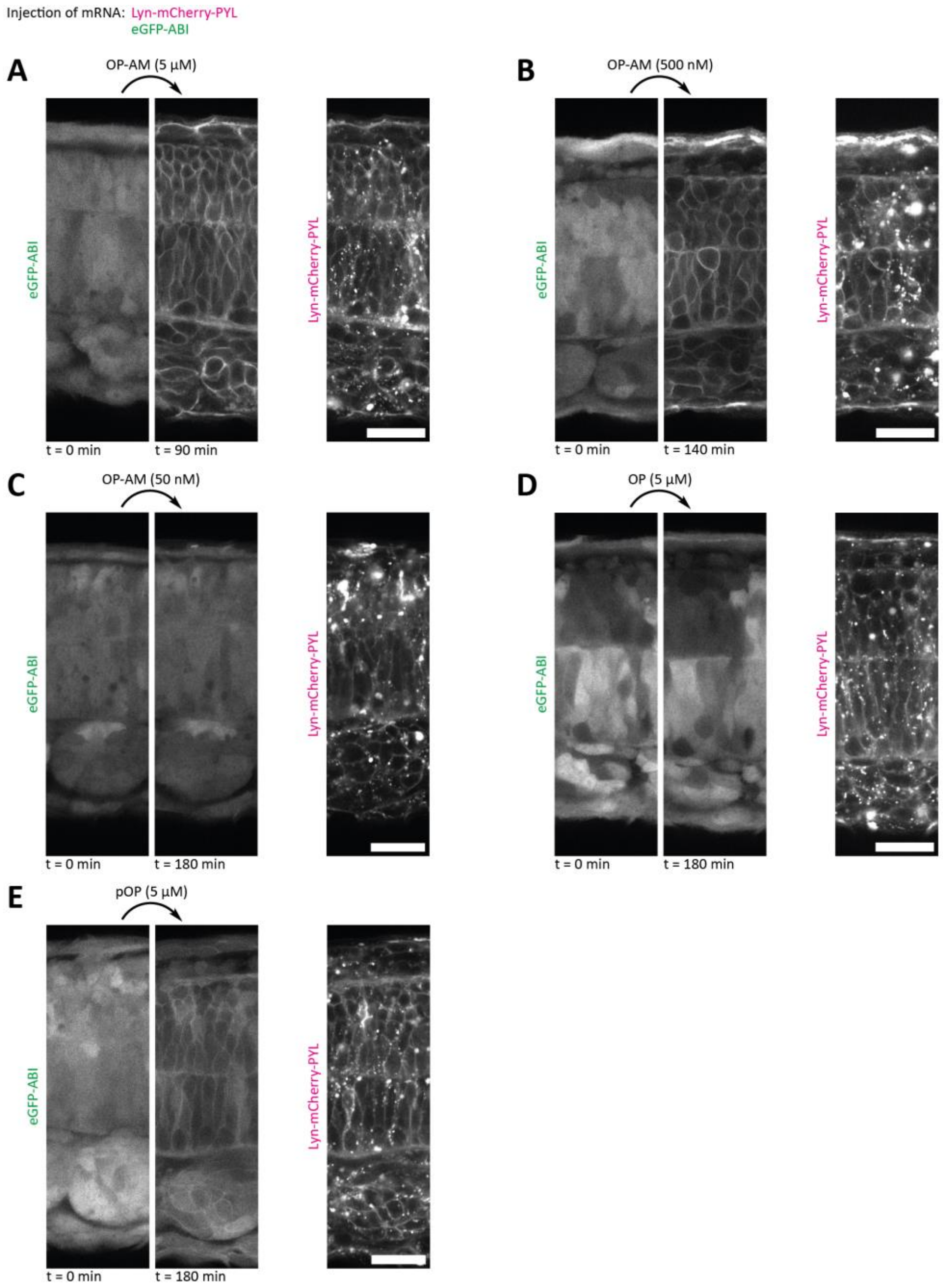

Figure S27: Overview of experiments with medaka embryos expressing Lyn-mCherry-PYL and eGFP-ABI. Confocal microscopy images, scale bars at 20 µm, selected ROI of the whole field of view depicted. Representative data of each two to three experiments. (A) Treatment with OP-AM induces protein proximity. Images before and 90 min after addition of 5 µM OP-AM. (B) Treatment with OP-AM induces protein proximity at nanomolar concentration. Images before and 140 min after addition of 500 nM OP-AM. (C) Treatment with a low nanomolar concentration of OP-AM does not induce protein proximity. Images before and 180 min after addition of 50 nM OP-AM. (D) Treatment with OP with a free carboxylic acid does not induce protein proximity. Images before and 180 min after addition of 5 µM OP. (E) Treatment with pOP induces protein proximity without irradiation. Images before and 180 min after addition of 5 µM pOP.

Concentration	5 µM	
Compound	OP-AM	
Receptor protein	PYL	
Prior addition	30 min post addition	60 min post addition
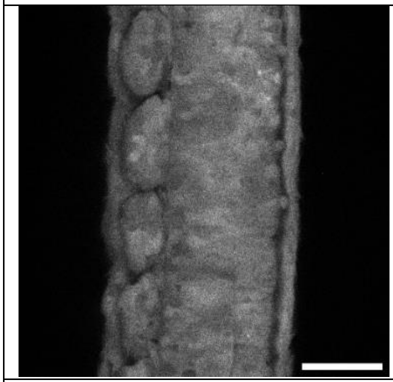	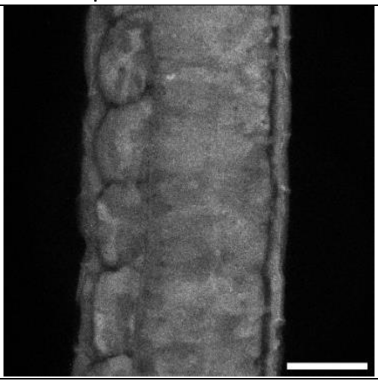	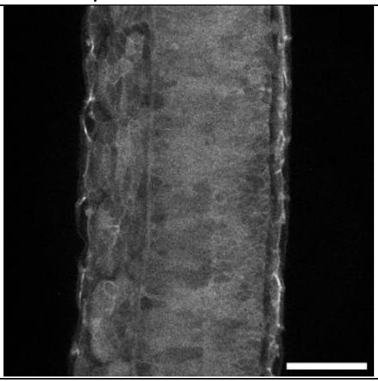
eGFP channel		
90 min post addition	110 min post addition	110 min post addition
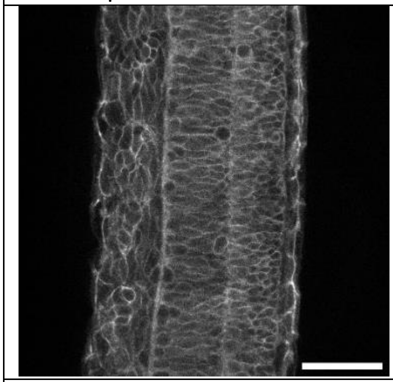	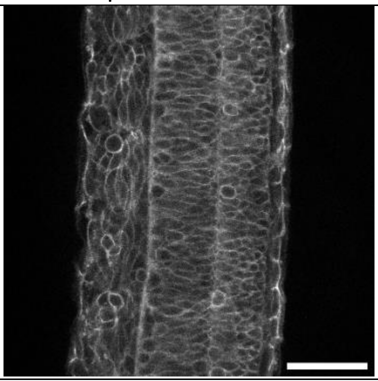	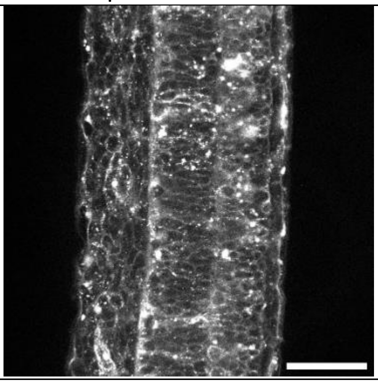
eGFP channel		mCherry channel

Figure S28: Addition of 5 µM OP-AM to medaka embryos expressing Lyn-mCherry-PYL and eGFP-ABI induces protein proximity. Confocal microscopy images of the tail of an medaka embryo mounted in agarose at different timepoints before and after addition of 5 µM OP-AM. Scale bars at 40 µm, whole field of view depicted. Representative data of three experiments.

Concentration	500 nM	
Compound	OP-AM	
Receptor protein	PYL	
Prior addition	30 min post addition	60 min post addition
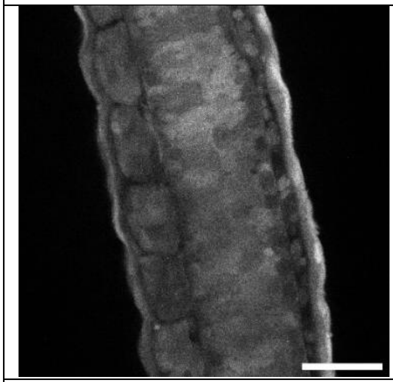	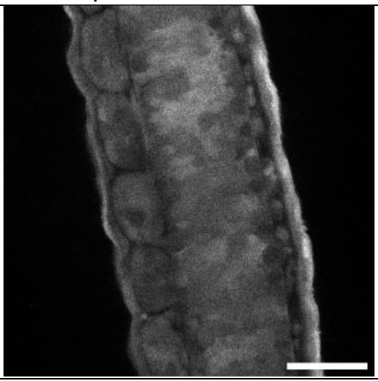	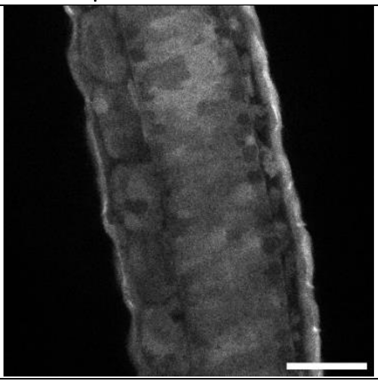
eGFP channel		
90 min post addition	120 min post addition	140 min post addition
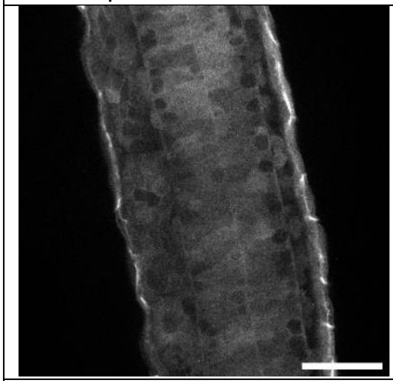	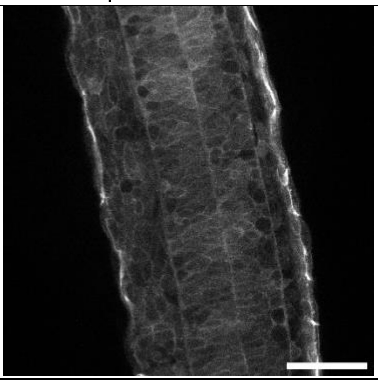	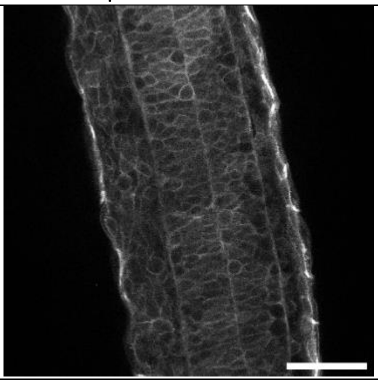
eGFP channel		
		140 min post addition
		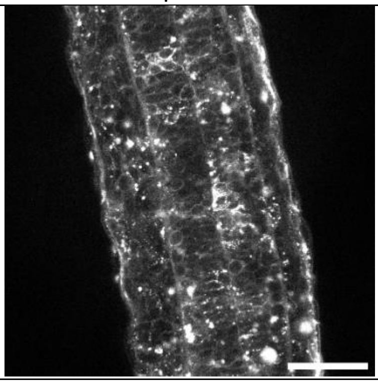
		mCherry channel

Figure S29: Addition of 500 nM OP-AM to medaka embryos expressing Lyn-mCherry-PYL and eGFP-ABI induces protein proximity. Confocal microscopy images of the tail of an medaka embryo mounted in agarose at different timepoints before and after addition of 500 nM OP-AM. Scale bars at 40 µm, whole field of view depicted. Representative data of three experiments.

Concentration	50 nM	
Compound	OP-AM	
Receptor protein	PYL	
Prior addition	180 min post addition	180 min post addition
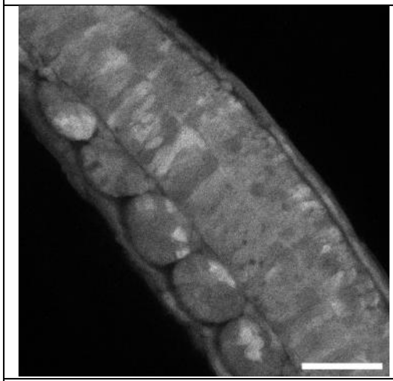	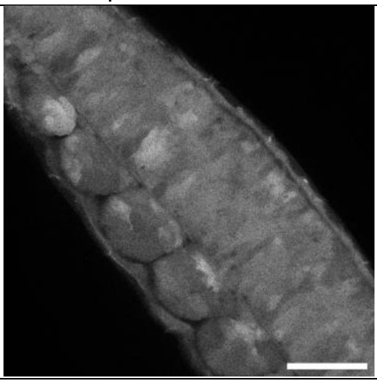	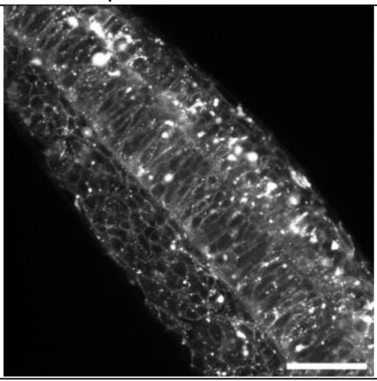
eGFP channel		mCherry channel

Figure S30: Addition of 50 nM OP-AM to medaka embryos expressing Lyn-mCherry-PYL and eGFP-ABI does not induce protein proximity. Confocal microscopy images of the tail of an medaka embryo mounted in agarose before and after addition of 50 nM OP-AM. Scale bars at 40 µm, whole field of view depicted. Representative data of three experiments.

Concentration	5 µM	
Compound	OP	
Receptor protein	PYL	
Prior addition	180 min post addition	180 min post addition
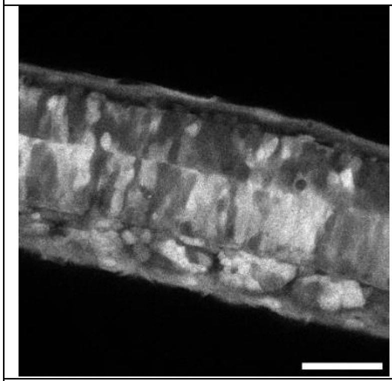	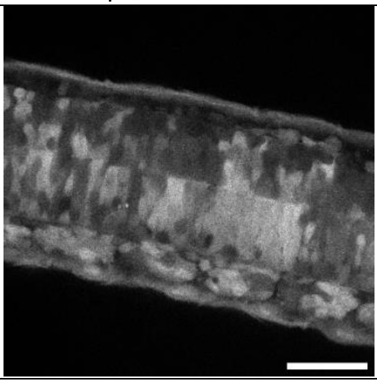	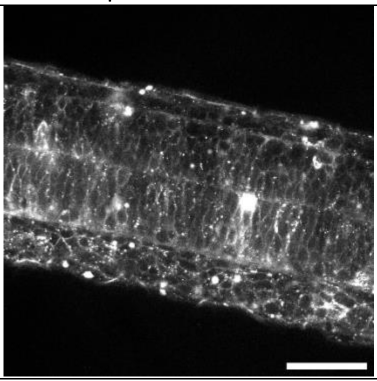
eGFP channel		mCherry channel

Figure S31: Addition of 5 µM OP to medaka embryos expressing Lyn-mCherry-PYL and eGFP-ABI does not induce protein proximity. Confocal microscopy images of the tail of an medaka embryo mounted in agarose before and after addition of 5 µM OP. Scale bars at 40 µm, whole field of view depicted. Representative data of three experiments.

Concentration	5 µM	
Compound	pOP	
Receptor protein	PYL	
Prior addition	180 min post addition	180 min post addition
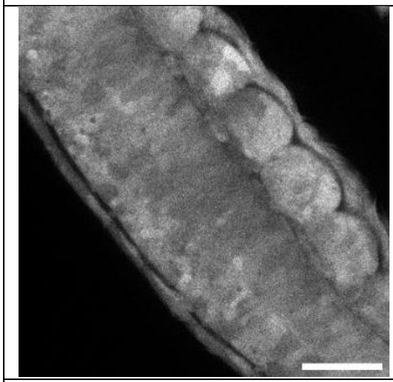	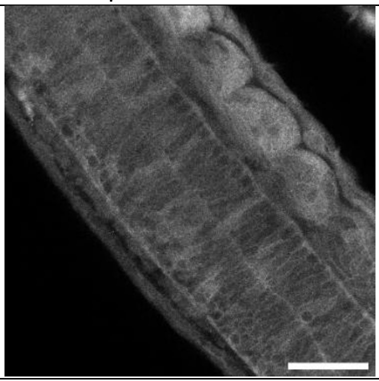	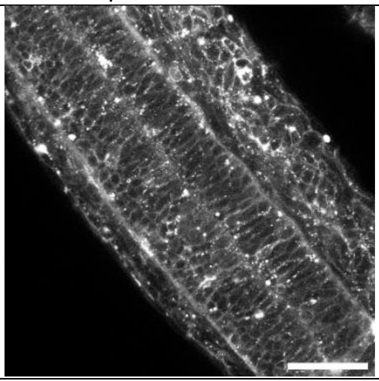
eGFP channel		mCherry channel

Figure S32: Addition of the photocaged molecule pOP to medaka embryos expressing Lyn-mCherry-PYL and eGFP-ABI induces protein proximity without irradiation. Confocal microscopy images of the tail of an medaka embryo mounted in agarose before and after addition of 5 µM pOP. Scale bars at 40 µm, whole field of view depicted. Representative data of two experiments.

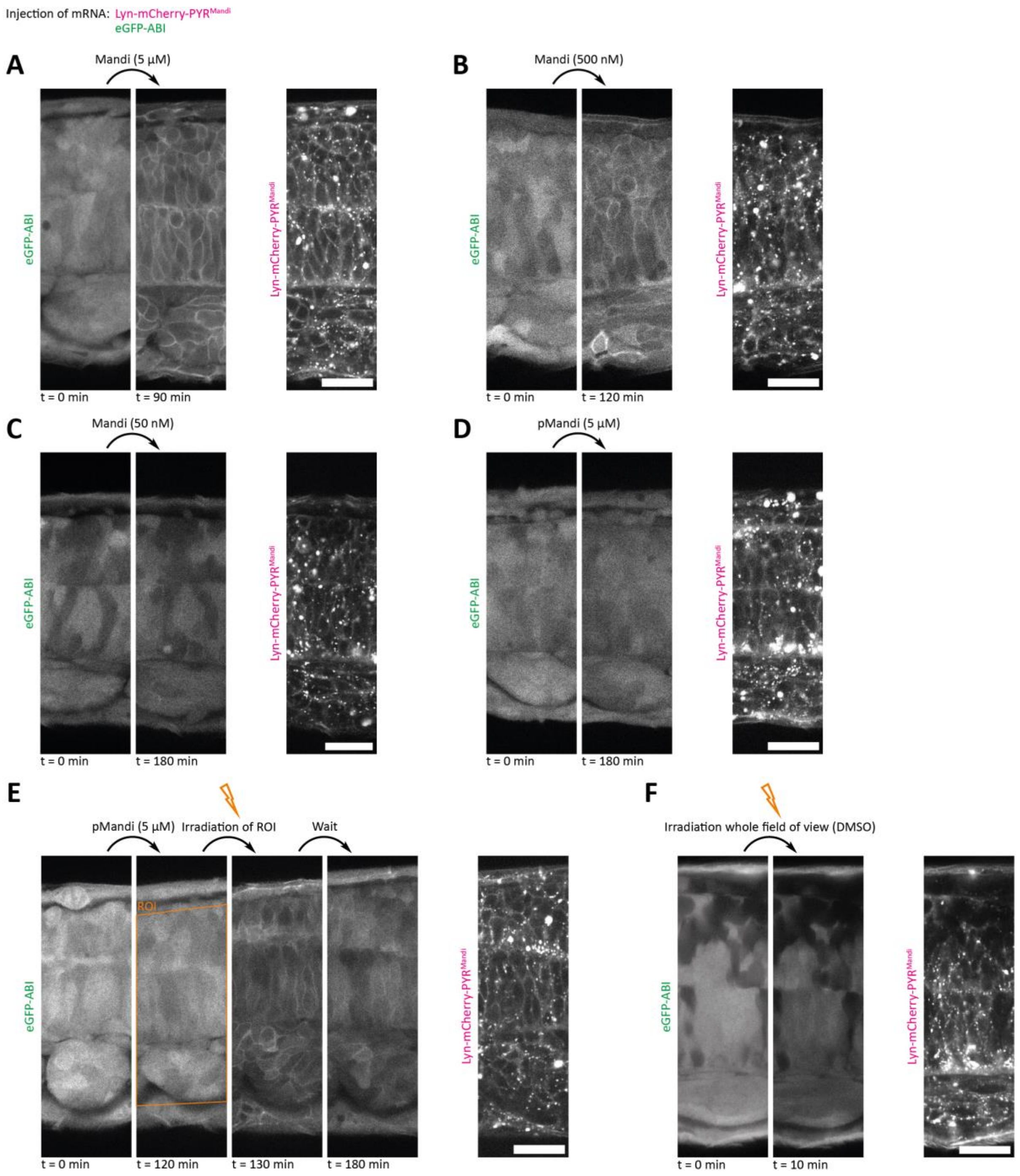

Figure S33: Overview of experiments with medaka embryos expressing Lyn-mCherry-PYR^{Mandi} and eGFP-ABI. Confocal microscopy images, scale bars at 20 µm, selected ROI of the whole field of view depicted. Representative data of each three to five experiments. (A) Treatment with Mandi induces protein proximity. Images before and 90 min after addition of 5 µM Mandi. (B) Treatment with Mandi induces protein proximity at nanomolar concentration. Images before and 120 min after addition of 500 nM Mandi. (C) Treatment with a low nanomolar concentration of Mandi does not induce protein proximity. Images before and 180 min after addition of 50 nM Mandi. (D) Treatment with pMandi does not induce protein proximity without irradiation. Images before and 180 min after addition of 5 µM pMandi. (E) Treatment with pMandi induces protein proximity upon irradiation (405 nm). Images before and 120 min after addition of 5 µM pMandi as well as 10 min and 60 min after local irradiation of the ROI covered by the medaka embryo in the field of view. Irradiation was performed immediately after image acquisition at 120 min post addition. (F) DMSO control: Irradiation (405 nm) alone does not induce protein proximity. Images before and 10 min after irradiation of the whole field of view.

Concentration	5 µM	
Compound	Mandi	
Receptor protein	PYR^{Mandi}	
Prior addition	30 min post addition	60 min post addition
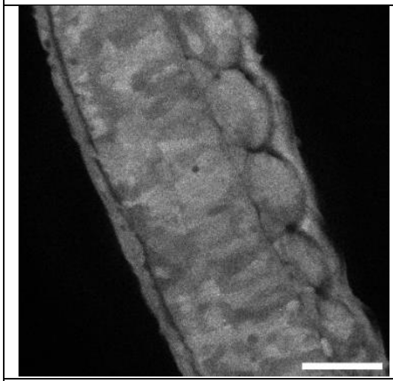	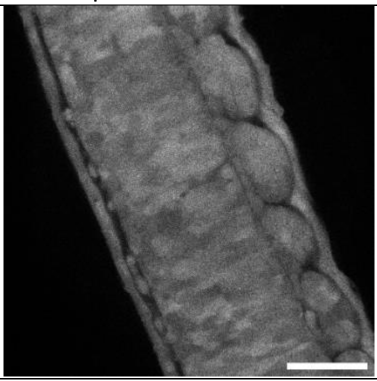	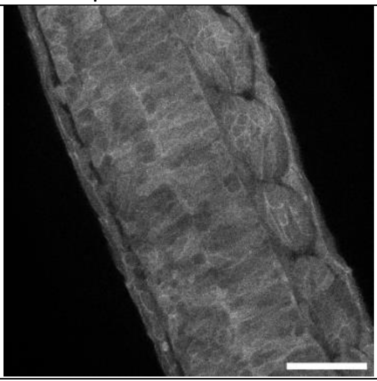
eGFP channel		
90 min post addition	100 min post addition	100 min post addition
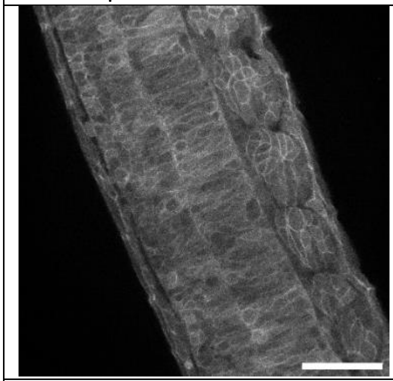	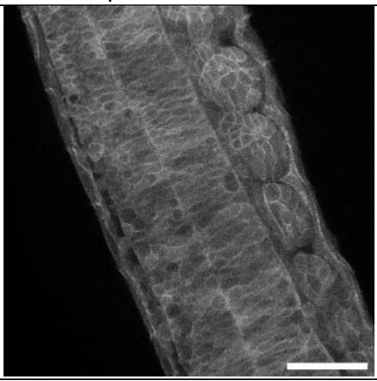	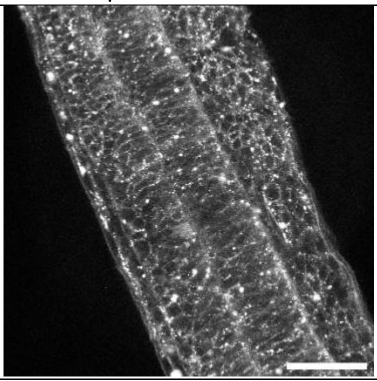
eGFP channel		mCherry channel

Figure S34: Addition of 5 µM Mandi to medaka embryos expressing Lyn-mCherry-PYR^{Mandi} and eGFP-ABI induces protein proximity. Confocal microscopy images of the tail of an medaka embryo mounted in agarose at different timepoints before and after addition of 5 µM Mandi. Scale bars at 40 µm, whole field of view depicted. Representative data of three experiments.

Concentration	500 nM	
Compound	Mandi	
Receptor protein	PYR[Mandi]	
Prior addition	30 min post addition	60 min post addition
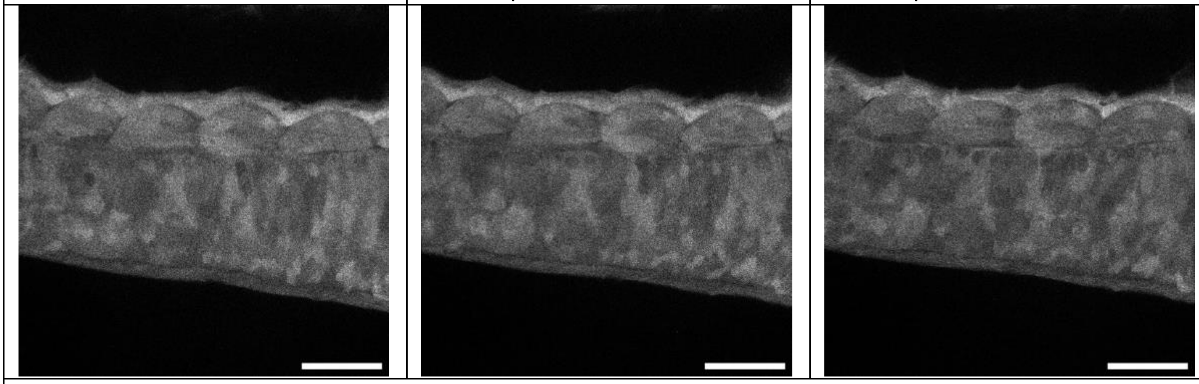		
eGFP channel		
90 min post addition	120 min post addition	120 min post addition
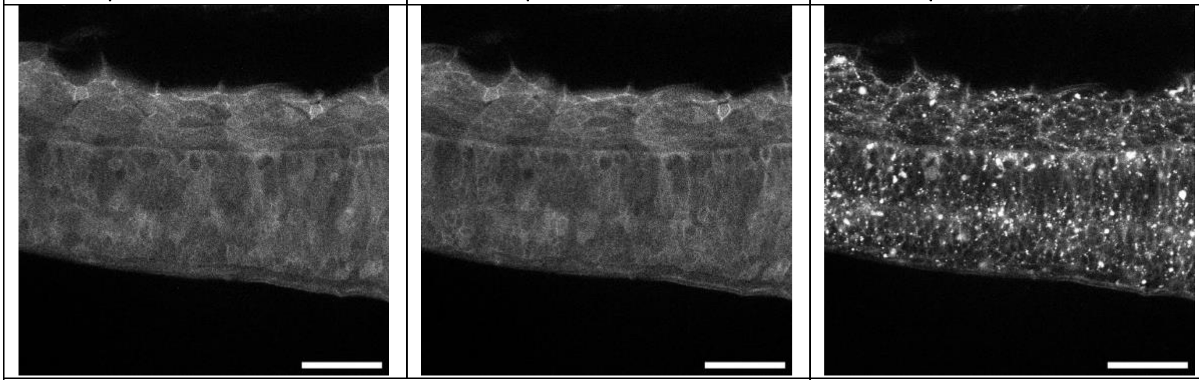		
eGFP channel		mCherry channel

Figure S35: Addition of 500 nM Mandi to medaka embryos expressing Lyn-mCherry-PYR[Mandi] and eGFP-ABI induces protein proximity. Confocal microscopy images of the tail of an medaka embryo mounted in agarose at different timepoints before and after addition of 500 nM Mandi. Scale bars at 40 µm, whole field of view depicted. Representative data of three experiments.

Concentration	50 nM	
Compound	Mandi	
Receptor protein	PYR^{Mandi}	
Prior addition	180 min post addition	180 min post addition
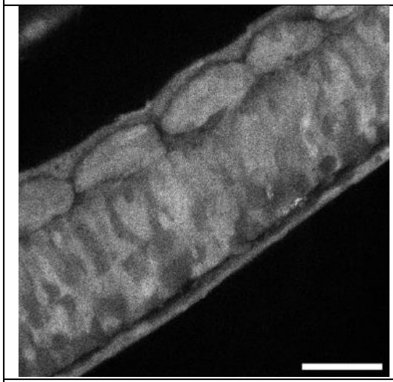	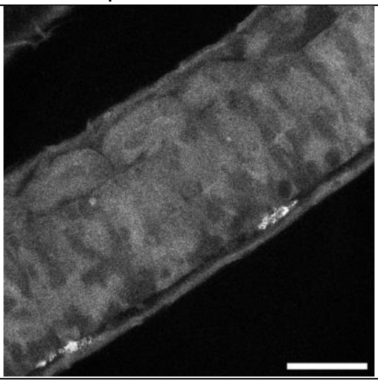	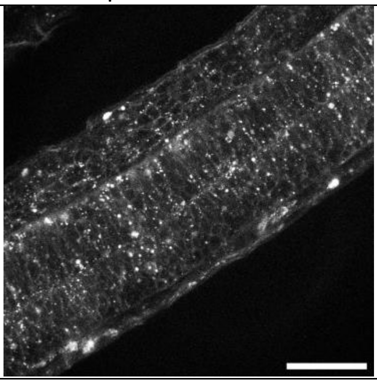
eGFP channel		mCherry channel

Figure S36: Addition of 50 nM Mandi to medaka embryos expressing Lyn-mCherry-PYR^{Mandi} and eGFP-ABI does not induce protein proximity. Confocal microscopy images of the tail of an medaka embryo mounted in agarose before and after addition of 50 nM Mandi. Scale bars at 40 µm, whole field of view depicted. Representative data of three experiments.

Concentration	5 µM	
Compound	pMandi	
Receptor protein	PYR^{Mandi}	
Prior addition	180 min post addition	180 min post addition
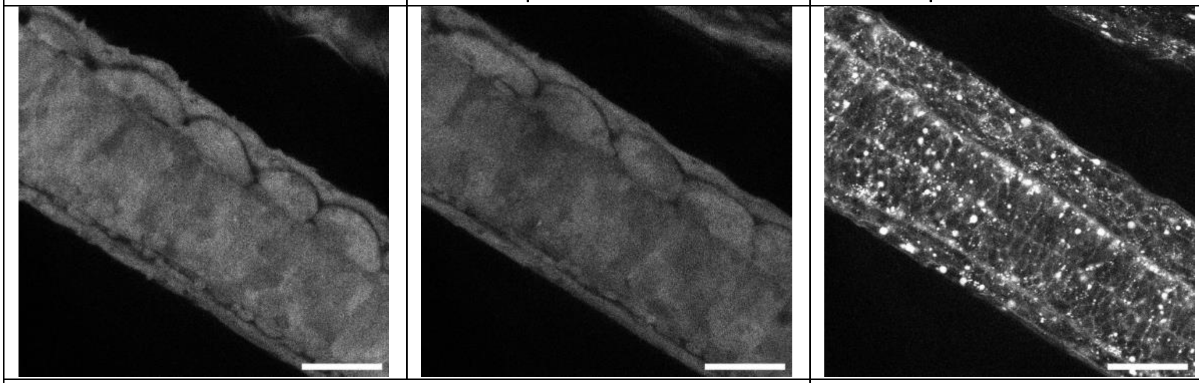		
eGFP channel		mCherry channel

Figure S37: Addition of the photocaged molecule pMandi to medaka embryos expressing Lyn-mCherry-PYR^{Mandi} and eGFP-ABI does not induce protein proximity without irradiation. Confocal microscopy images of the tail of an medaka embryo mounted in agarose before and after addition of 5 µM pMandi. Scale bars at 40 µm, whole field of view depicted. Representative data of four experiments.

Concentration		
Compound	DMSO	
Receptor protein	PYR^{Mandi}	
Prior irradiation	10 min post irradiation	10 min post irradiation
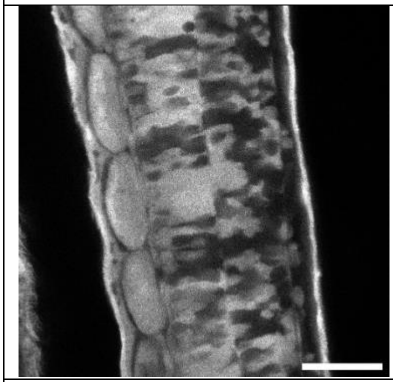	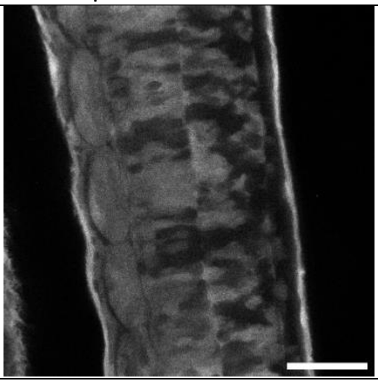	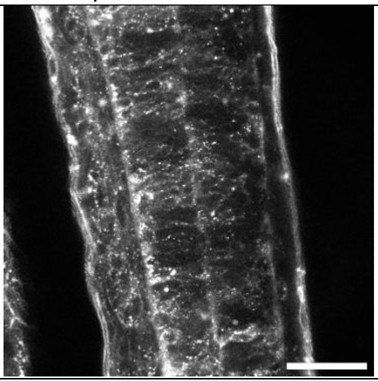
eGFP channel		mCherry channel

Figure S38: Irradiation (405 nm) of medaka embryos expressing Lyn-mCherry-PYR^{Mandi} and eGFP-ABI and treated with DMSO (0.5 %) at the confocal microscope does not induce protein proximity. Confocal microscopy images of the tail of an medaka embryo mounted in agarose at different timepoints before and after irradiation (405 nm) of the whole field of view. Scale bars at 40 µm, whole field of view depicted. Representative data of four experiments.

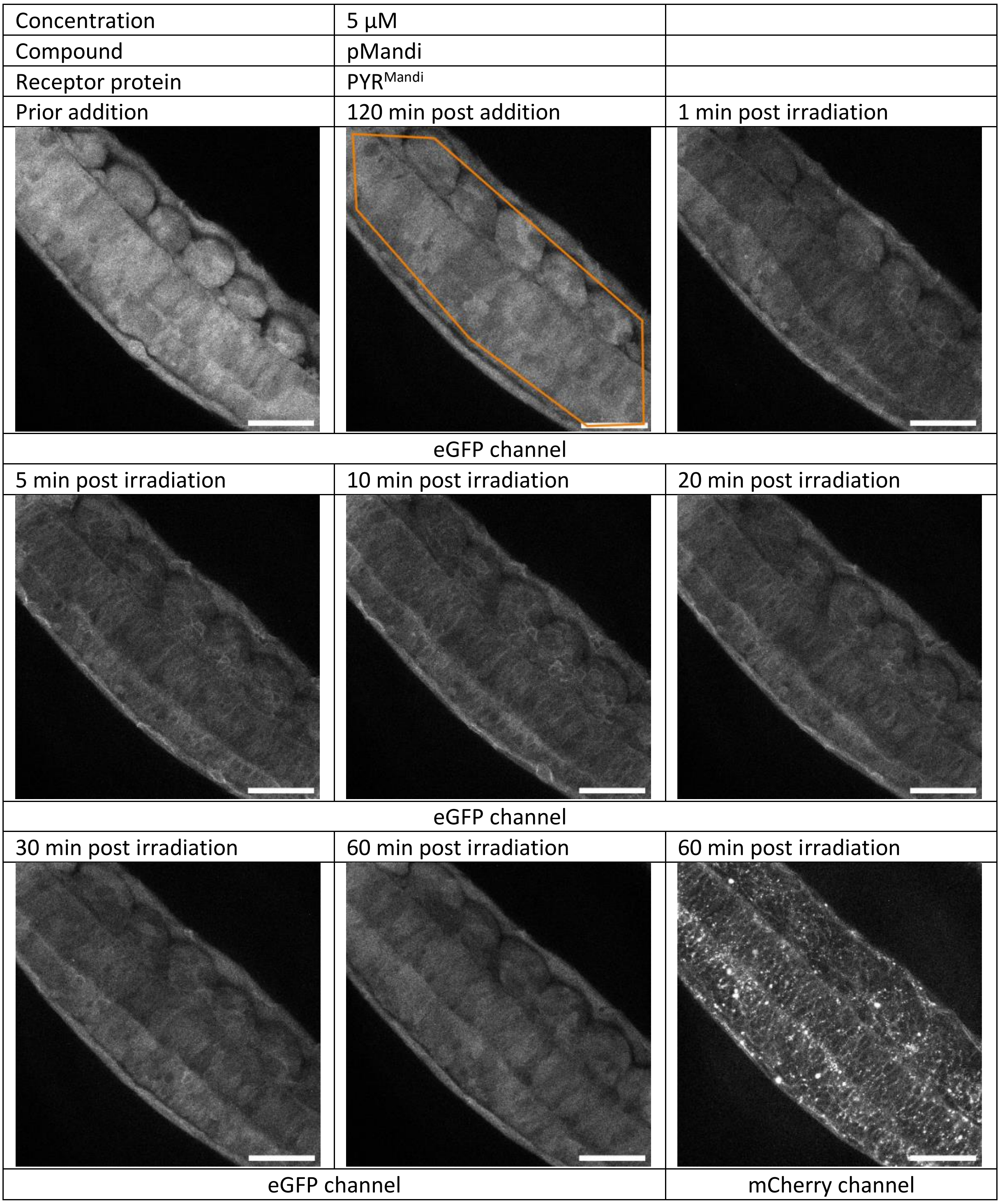

Figure S39: Time course of irradiation experiment with medaka embryos expressing Lyn-mCherry-PYR^{Mandi} and eGFP-ABI and treated with pMandi at the confocal microscope. Protein proximity is induced upon irradiation and reverses over time thereafter. Confocal microscopy images of the tail of an medaka embryo mounted in agarose at different timepoints before and after addition of 5 µM pMandi and after different timepoints after local irradiation (405 nm) of the ROI covered by the medaka embryo in the field of view (area outlined in orange). Irradiation was performed immediately ***after*** image acquisition at 120 min post addition. Scale bars at 40 µm, whole field of view depicted. Representative data of five experiments.

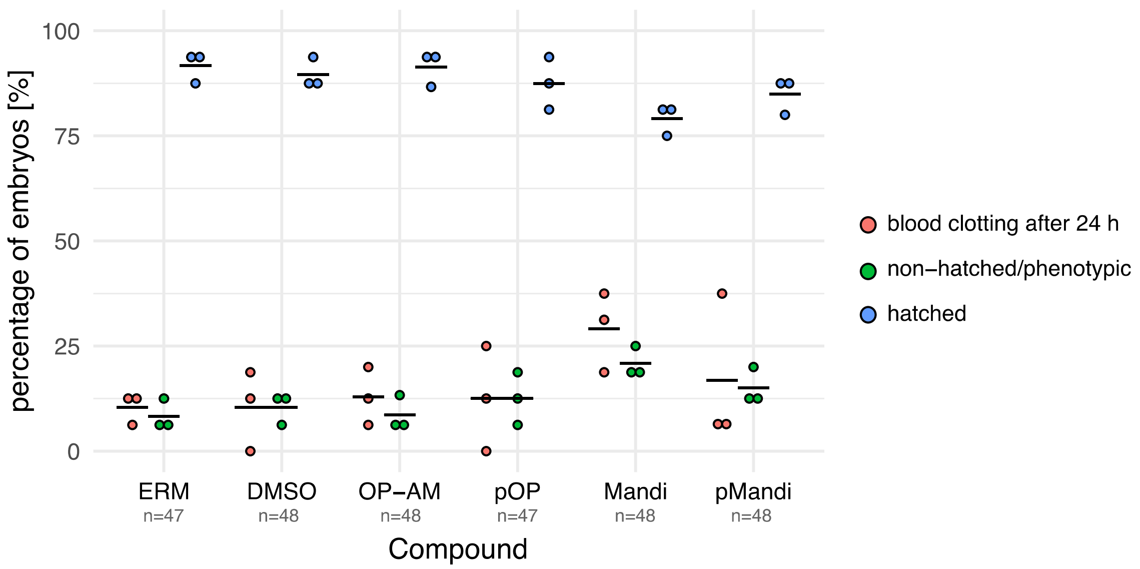

Figure S40: Toxicity test of OP-AM, pOP, Mandi and pMandi in medaka embryos. At 2 days post fertilization, medaka embryos were incubated with 5 µM of each compound and 0.1 % DMSO as well as 1x ERM only. Three independent experiments were performed with 15-16 embryos per condition in each experiment. After 3 h and 6 h no phenotypes were visible. After 24 h blood clotting was visible in a few embryos. Many individuals recovered from the blood clotting after media was changed to 1x ERM after 24 h of compound incubation.

2 Methods

2.1 In vitro irradiation experiments

For in vitro irradiation experiments solutions of the compounds were prepared with a concentration of 20 µM in a 1:1 mixture of acetonitrile and PBS containing 2 % of DMSO. The solutions were prepared from stock solutions in DMSO of a concentration of 1 mM and diluted with equal amounts of acetonitrile and PBS to a total volume of 1 ml.

The UV/Vis measurements were performed with a V-770 UV-Visible-Near Infrared Spectrophotometer (Jasco) in appropriate cuvettes with a total volume of 1 ml at 25 °C. Irradiation was performed with a SOLIS-365C LED (Thorlabs, nominal wavelength 365 nm, bandwidth (FWHM) 10 nm) and a SOLIS-405C LED (Thorlabs, nominal wavelength 405 nm, bandwidth (FWHM) 14 nm) controlled with a DC2200 driver (Thorlabs) and a photocurrent of 100 mA for pMandi or 1000 mA for pOP. The light source for irradiation was connected via a light guide to the top of the cuvette that was placed in the spectrometer. The samples were irradiated stepwise und the UV/Vis spectra were recorded between the individual irradiation periods.

For HPLC analysis the whole sample volume was injected into an UltiMate 3000 HPLC system (Thermo Fisher Scientific) equipped with an Ascentis C18 column (Supleco, 25 cm x 10 mm, 5 µm). A gradient acetonitrile:water + 0.1 % TFA from 90:10 to 10:90 was used and the absorption was detected from 200 nm to 800 nm over time (bandwidth 2 nm). The HPLC traces are shown at the absorption maxima of the compounds. A flow rate of 4 ml min^{-1} was used.

To investigate the decay of pMandi and pOP quantitively, samples were irradiated for different times and analyzed by HPLC. The peak area of pMandi or pOP was determined and normalized to the peak area of a reference injection of the respective compound in a known concentration (pOP: HPLC trace at 300 nm, pMandi: HPLC trace at 280 nm). The following exponential decay was fitted to the data with OriginPro 2021 (v9.8.0.200):

$$y = A_1\left(e^{-\frac{x}{t_1}}\right) + y_0\,,$$

with the molar concentration y, the offset y_0, the amplitude A_1 and the time constant t_0. The initial slope (rate of decay) was calculated to minimize errors due to light absorption of decay products after photolysis. To set the rate of decay in relation to the photon influx and thus to calculated the quantum yield of photouncaging the fraction of light absorbed by compound and the power of the light used for irradiation was determined.

The fraction of light absorbed by the compounds was determined with the Beer-Lambert law via the extinction coefficient.

To determine the power of the light used for irradiation, the output power was measured with a power meter at the outlet of the light guide. To further correct for light beam broadening and therefore light not hitting the sample in this setup a reference measurement was performed. The light power hitting the sample was determined using ortho-nitrobenzaldehyde (2 mM in acetonitrile) as chemical actinometer (photolysis quantum yield in acetonitrile $\Phi = 50\,\%$)[1]. Samples were irradiated for different times and analyzed by HPLC. The peak area of ortho-nitrobenzaldehyde was determined and normalized to a reference injection of ortho-nitrobenzaldehyde in a known concentration (HPLC trace at 300 nm). This revealed that 30 % of light power at the outlet of the light guide hit the sample, which corresponded to measurements of the light power behind an empty cuvette with a power meter. The light power was corrected by this factor and the photon influx calculated.

Values from the light power measurements:

Wavelength [nm]	Photocurrent [mA]	Output power at the outlet of the light guide [mW]	Light power hitting the sample in the in vitro irradiation experiments [mW]
365	100	6	2
365	1000	62	20
405	100	20	6
405	1000	217	70

To obtain the quantum yield the photon influx was multiplied with the fraction of fraction of light absorbed by compound and set in relation to the respective rate of decay.

Determined values:

Compound	Extinction coefficient $\varepsilon_{405\ nm}$ [M^{-1} cm^{-1}]	Quantum yield Φ [%]	Photochemical efficiency / uncaging cross section ε_u [M^{-1} cm^{-1}]
pMandi	526	2.2	12
pOP	428	0.4	2

2.2 Cloning and plasmid preparation

For the construction of plasmids, fragments were amplified by PCR from appropriate sources with primers purchased from Sigma-Aldrich. The PCR reaction mixture was digested with DpnI (Thermo Scientific), analyzed by agarose gel electrophoresis and purified using the QIAquick PCR Purification Kit (Qiagen). Then, Gibson Assembly was performed and plasmids were purified using the QIAprep Spin Miniprep Kit (Qiagen). The plasmid sequences were validated by Sanger sequencing (Eurofins) using standard and custom-made primers.

List of plasmids:

Coding sequence (CDS)	Plasmid backbone
TOMM20-mCherry-PYR^{Mandi}-P2A-eGFP-ABI	pcDNA5/FRT
TOMM20-eGFP-PYL-P2A-eGFP-ABI	pcDNA5/FRT
eGFP-ABI	pCS2+
Lyn-mCherry-PYR^{Mandi}	pCS2+
Lyn-mCherry-PYL	pCS2+

List of fragments, corresponding PCR primers used for plasmid construction and sequencing primers used for validation of the plasmid sequences:

Plasmid	Fragments	PCR primers	Sequencing primers
pcDNA5/FRT-TOMM20-mCherry-PYR^{Mandi}	TOMM20-mCherry-PYR^{Mandi} from pTOMM20-mCherry-PYRMandi-IRES-EGFP-ABI[2]	#1, #2	CMVfor, pcDNA3_for, pcDNA3.1-RP_1, pCR3.1-BGHrev
	pcDNA5/FRT Vector from Thermo Fisher Scientific	#3, #4	
pcDNA5/FRT-eGFP-ABI	eGFP-ABI from pTOMM20-mCherry-PYR^{Mandi}-IRES-EGFP-ABI[2]	#5, #6	CMVfor, pcDNA3.1-RP_1
	pcDNA5/FRT Vector from Thermo Fisher Scientific	#7, #8	
pcDNA5/FRT-TOMM20-mCherry-PYR^{Mandi}-P2A-eGFP-ABI	P2A-eGFP-ABI from pcDNA5/FRT-eGFP-ABI	#9, #10	CMVfor, pcDNA3.1-RP_1, EGFP-Nrev, PYRMandi_fw (ATAGTAATATCCGGTCTCCCTGC)
	TOMM20-mCherry-PYR^{Mandi} from pcDNA5/FRT-TOMM20-mCherry-PYRMandi	#11, #12	
pcDNA5/FRT-TOMM20-eGFP-PYL-P2A-eGFP-ABI	pcDNA5/FRT-TOMM20-mCherry-PYR^{Mandi}-P2A-eGFP-ABI	#13, #14	CMVfor, pcDNA3.1-RP_1, EGFP-Nrev, ABI_rev (AGCCACGTATCACCATCGCAG)
	TOMM20-mCherry-PYL from pTOMM20-mCherry-PYLI[2]	#15, #16	

pcDNA5/FRT-Lyn-mCherry-PYR^{Mandi}	Lyn-mCherry-PYR^{Mandi} from pLyn-mCherry-PYR^{Mandi}[2]	#17, #18	pcDNA3_for, pcDNA3.1-RP_1
	pcDNA5/FRT Vector from Thermo Fisher Scientific	#19, #20	
pcDNA5/FRT-Lyn-mCherry-PYL	Lyn-mCherry-PYL from pLyn-mCherry-PYL[2]	#21, #22	pcDNA3_for, pcDNA3.1-RP_1
	pcDNA5/FRT Vector from Thermo Fisher Scientific	#23, #24	
pCS2+-eGFP-ABI	eGFP-ABI from pcDNA5/FRT-eGFP-ABI	#25, #26	CMVfor, SP6, pCEP-Reverse
	pCS2+ Vector	#27, #28	
pCS2+-Lyn-mCherry-PYR^{Mandi}	Lyn-mCherry-PYR^{Mandi} from pcDNA5/FRT-Lyn-mCherry-PYR^{Mandi}	#29, #30	CMVfor, SP6, pCEP-Reverse
	pCS2+ Vector	#31, #32	
pCS2+-Lyn-mCherry-PYL	Lyn-mCherry-PYL from pcDNA5/FRT- Lyn-mCherry-PYL	#33, #34	CMVfor, SP6, pCEP-Reverse
pCS2+-Lyn-mCherry-PYL	pCS2+ Vector	#35, #36	CMVfor, SP6, pCEP-Reverse

List of PCR primers and their sequences used for plasmid construction:

PCR primers	Sequence (5’-3’)
#1	GTTCCGACCCATGGTGGTAAGTTTAAACGCT
#2	CAGGTCACGTAGTCTAGAGGGCCCGTTTA
#3	ACCACCATGGGTCGGAACAGCGCCAT
#4	TCTAGACTACGTGACCTGGCTGCCAG
#5	TGATTTGAAGTAGTCTAGAGGGCCCGTTTA
#6	CTTGCTCACCATGGTGGTAAGTTTAAACGCT
#7	ACCACCATGGTGAGCAAGGGCGAGGAG
#8	CTCTAGACTACTTCAAATCAACCACCACCACA
#9	CAGGTCACGGGCTCTGGTGCCACAAACTTCTCTCTGCTAAAGCAAGCAGGTGATGTTGAA GAAAACCCCGGGCCTGGCGGTTCTATGGTGAGCAAGGGCGAG
#10	CTCTAGACTACTTCAAATCAACCACCACCACA
#11	TGATTTGAAGTAGTCTAGAGGGCCCGTTTA
#12	ACCAGAGCCCGTGACCTGGCTGCCAG
#13	CTTACCACCATGGGTCGGAACAGCGC
#14	ACCAGAGCCGTTCATAGCTTCAGTGATCGAAGC
#15	GCTATGAACGGCTCTGGTGCCACAAACTT
#16	CCGACCCATGGTGGTAAGTTTAAACGCTAGCC
#17	GGAGGACCAATGCCATCTGAATTGACCCCT
#18	ACATCCCATGGTGGTAAGTTTAAACGCTAGCC
#19	CTTACCACCATGGGATGTATTAAATCAAAAAGGA
#20	AGATGGCATTGGTCCTCCTGCGCTCTT
#21	GGAGGACCAACTCAAGACGAATTCACCCAAC
#22	CCGACCCATGGTGGTAAGTTTAAACGCTAGCC
#23	CTTACCACCATGGGTCGGAACAGCGC
#24	GTCTTGAGTTGGTCCTCCTGCGCTCTT

#25	TTCGAATTCGCTGCCAACATGGTGAGCAAGGGCGAG
#26	ATAGTTCTAGACTACTTCAAATCAACCACCACCAC
#27	ATTTGAAGTAGTCTAGAACTATAGTGAGTCGT
#28	GTTGGCAGCGAATTCGAATCGATGGGATCCTG
#29	TTCGAATTCGCTGCCAACATGGGATGTATTAAATCAAAAAGGA
#30	AGTTCTAGACTACGTGACCTGGCTGCC
#31	GTCACGTAGTCTAGAACTATAGTGAGTCGT
#32	GTTGGCAGCGAATTCGAATCGATGGGATCCTG
#33	TTCGAATTCGCTGCCAACATGGGATGTATTAAATCAAAAAGGA
#34	ATAGTTCTAGACTAGTTCATAGCTTCAGTGATCGA
#35	CTATGAACTAGTCTAGAACTATAGTGAGTCGT
#36	GTTGGCAGCGAATTCGAATCGATGGGATCCTG

List of sequences of the gene fragments:

Name	TOMM20
Sequence (5’-3’)	ATGGGTCGGAACAGCGCCATCGCCGCGGGCGTGTGCGGTGCCCTCTTCATAGGGTACTGCAT<br>CTACTTTGACCGCAAAAGACGAAGTGACCCCAACTTC
Name	Lyn
Sequence (5’-3’)	ATGGGATGTATTAAATCAAAAAGGAAAGAC
Name	mCherry
Sequence (5’-3’)	ATGGTGAGCAAGGGCGAGGAGGATAACATGGCCATCATCAAGGAGTTCATGCGCTTCAAGG<br>TGCACATGGAGGGCTCCGTGAACGGCCACGAGTTCGAGATCGAGGGCGAGGGCGAGGGCC<br>GCCCCTACGAGGGCACCCAGACCGCCAAGCTGAAGGTGACCAAGGGTGGCCCCCTGCCCTTC<br>GCCTGGGACATCCTGTCCCCTCAGTTCATGTACGGCTCCAAGGCCTACGTGAAGCACCCCGCC<br>GACATCCCCGACTACTTGAAGCTGTCCTTCCCCGAGGGCTTCAAGTGGGAGCGCGTGATGAA<br>CTTCGAGGACGGCGGCGTGGTGACCGTGACCCAGGACTCCTCCCTGCAGGACGGCGAGTTC<br>ATCTACAAGGTGAAGCTGCGCGGCACCAACTTCCCCTCCGACGGCCCCGTAATGCAGAAGAA<br>GACCATGGGCTGGGAGGCCTCCTCCGAGCGGATGTACCCCGAGGACGGCGCCCTGAAGGGC<br>GAGATCAAGCAGAGGCTGAAGCTGAAGGACGGCGGCCACTACGACGCTGAGGTCAAGACC<br>ACCTACAAGGCCAAGAAGCCCGTGCAGCTGCCCGGCGCCTACAACGTCAACATCAAGTTGGA<br>CATCACCTCCCACAACGAGGACTACACCATCGTGGAACAGTACGAACGCGCCGAGGGCCGCC<br>ACTCCACCGGCGGCATGGACGAACTGTACAAG
Name	eGFP
Sequence (5’-3’)	ATGGTGAGCAAGGGCGAGGAGCTGTTCACCGGGGTGGTGCCCATCCTGGTCGAGCTGGACG<br>GCGACGTAAACGGCCACAAGTTCAGCGTGTCCGGCGAGGGCGAGGGCGATGCCACCTACGG<br>CAAGCTGACCCTGAAGTTCATCTGCACCACCGGCAAGCTGCCCGTGCCCTGGCCCACCCTCGT<br>GACCACCCTGACCTACGGCGTGCAGTGCTTCAGCCGCTACCCCGACCACATGAAGCAGCACG<br>ACTTCTTCAAGTCCGCCATGCCCGAAGGCTACGTCCAGGAGCGCACCATCTTCTTCAAGGACG<br>ACGGCAACTACAAGACCCGCGCCGAGGTGAAGTTCGAGGGCGACACCCTGGTGAACCGCAT<br>CGAGCTGAAGGGCATCGACTTCAAGGAGGACGGCAACATCCTGGGGCACAAGCTGGAGTAC<br>AACTACAACAGCCACAACGTCTATATCATGGCCGACAAGCAGAAGAACGGCATCAAGGTGA<br>ACTTCAAGATCCGCCACAACATCGAGGACGGCAGCGTGCAGCTCGCCGACCACTACCAGCAG<br>AACACCCCCATCGGCGACGGCCCCGTGCTGCTGCCCGACAACCACTACCTGAGCACCCAGTC<br>CGCCCTGAGCAAAGACCCCAACGAGAAGCGCGATCACATGGTCCTGCTGGAGTTCGTGACCG<br>CCGCCGGGATCACTCTCGGCATGGACGAGCTGTACAAG

Name	PYR^{Mandi}
Sequence (5’-3’)	ATGCCATCTGAATTGACCCCTGAGGAACGCTCCGAATTGAAAAATTCAATCGCCGAATTCCAT<br>ACCTATCAGCTCGACCCCGGATCTTGCAGTTCACTGCATGCACAGCGCATCCACGCGCCCCCA<br>GAATTGGTGTGGTCTATCGTTCGCCGCTTTGACAAACCCCAAACGCACCGGCACTTCATAAAG<br>TCATGTTCAGTTGAACAGAATTTCGAAATGCGAGTGGGCTGCACCAGAGATATAATAGTAAT<br>ATCCGGTCTCCCTGCAAATACATCCACGGAGCGACTGGACATACTTGACGATGAAAGAAGAG<br>TTACGGGCGCTTCTATAATTGGGGGCGAACACCGGCTGACTAACTATAAGGGCGTCACAACC<br>GTTCACCGCTTCGAGAAGGAAAACCGCATCTGGACTGTAGTGTTGGAAAGCTATGTAGTGGA<br>TATGCCTGAAGGAAATTCTGAAGACGACACTAGGATGCTTGCGGATACAGTCGTCAAACTTA<br>ACCTCCAGAAACTTGCTACTGTAGCGGAGGCTATGGCCCGGAACTCAGGTGATGGCTCTGGC<br>AGCCAGGTCACG
Name	PYL
Sequence (5’-3’)	ACTCAAGACGAATTCACCCAACTCTCCCAATCAATCGCCGAGTTCCACACGTACCAACTCGGT<br>AACGGCCGTTGCTCATCTCTCCTAGCTCAGCGAATCCACGCGCCGCCGGAAACAGTATGGTC<br>CGTGGTGAGACGTTTCGATAGGCCACAGATTTACAAACACTTCATCAAAAGCTGTAACGTGA<br>GTGAAGATTTCGAGATGCGAGTGGGATGCACGCGCGACGTGAACGTGATAAGTGGATTACC<br>GGCGAATACGTCTCGAGAGAGATTAGATCTGTTGGACGATGATCGGAGAGTGACTGGGTTT<br>AGTATAACCGGTGGTGAACATAGGCTGAGGAATTATAAATCGGTTACGACGGTTCATAGATT<br>TGAGAAAGAAGAAGAAGAAGAAAGGATCTGGACCGTTGTTTTGGAATCTTATGTTGTTGATG<br>TACCGGAAGGTAATTCGGAGGAAGATACGAGATTGTTTGCTGATACGGTTATTAGATTGAAT<br>CTTCAGAAACTTGCTTCGATCACTGAAGCTATGAAC
Name	ABI
Sequence (5’-3’)	GTGCCTTTGTATGGTTTTACTTCGATTTGTGGAAGAAGACCTGAGATGGAAGCTGCTGTTTCG<br>ACTATACCAAGATTCCTTCAATCTTCCTCTGGTTCGATGTTAGATGGTCGGTTTGATCCTCAAT<br>CCGCCGCTCATTTCTTCGGTGTTTACGACGGCCATGGCGGTTCTCAGGTAGCGAACTATTGTA<br>GAGAGAGGATGCATTTGGCTTTGGCGGAGGAGATAGCTAAGGAGAAACCGATGCTCTGCGA<br>TGGTGATACGTGGCTGGAGAAGTGGAAGAAAGCTCTTTTCAACTCGTTCCTGAGAGTTGACT<br>CGGAGATTGAGTCAGTTGCGCCGGAGACGGTTGGGTCAACGTCGGTGGTTGCCGTTGTTTC<br>CCGTCTCACATCTTCGTCGCTAACTGCGGTGACTCTAGAGCCGTTCTTTGCCGCGGCAAAACT<br>GCACTTCCATTATCCGTTGACCATAAACCGGATAGAGAAGATGAAGCTGCGAGGATTGAAGC<br>CGCAGGAGGGAAAGTGATTCAGTGGAATGGAGCTCGTGTTTTCGGTGTTCTCGCCATGTCGA<br>GATCCATTGGCGATAGATACTTGAAACCATCCATCATTCCTGATCCGGAAGTGACGGCTGTGA<br>AGAGAGTAAAAGAAGATGATTGTCTGATTTTGGCGAGTGACGGGGTTTGGGATGTAATGAC<br>GGATGAAGAAGCGTGTGAGATGGCAAGGAAGCGGATTCTCTTGTGGCACAAGAAAAACGC<br>GGTGGCTGGGGATGCATCGTTGCTCGCGGATGAGCGGAGAAAGGAAGGGAAAGATCCTGC<br>GGCGATGTCCGCGGCTGAGTATTTGTCAAAGCTGGCGATACAGAGAGGAAGCAAAGACAAC<br>ATAAGTGTGGTGGTGGTTGATTTGAAG
Name	Linker-P2A-Linker
Sequence (5’-3’)	GGCTCTGGTGCCACAAACTTCTCTCTGCTAAAGCAAGCAGGTGATGTTGAAGAAAACCCCGG<br>GCCTGGCGGTTCT
Name	Flexible linker between TOMM20 and mCherry and between Lyn and mCherry
Sequence (5’-3’)	GGATCCGGAGCAAGTGGA
Name	Flexible linker between eGFP and ABI
Sequence (5’-3’)	AGCGCAGGAGGAACGCGT

Name	Flexible linker between mCherry and PYR^{Mandi} and between mCherry and PYL
Sequence (5'-3')	AGCGCAGGAGGACCA
Name	Stop codon
Sequence (5'-3')	TAG
Name	Kozak sequence in pCS2+ plasmids
Sequence (5'-3')	GCTGCCAAC

2.3 Cell culture

2.3.1 General conditions

Cells were grown at 37 °C and 5 % CO_2 in Dulbecco's Modified Eagle Medium (DMEM; Sigma-Aldrich) supplemented with 2 mM L-glutamine, 1 mM sodium pyruvate and 10 % (vol/vol) fetal bovine serum. Cells were routinely passaged after 2 to 3 days or upon reaching 80 % confluency.

2.3.2 Generation of stable cell lines

Stable cell lines were generated with U2OS FlpIN TREx cells using the Flp-In System (Thermo Fisher Scientific) according to the manufacturer's protocol with the plasmids pcDNA5/FRT-TOMM20-mCherry-PYR^{Mandi}-P2A-eGFP-ABI and pcDNA5/FRT-TOMM20-eGFP-PYL-P2A-eGFP-ABI.

2.3.3 Sample preparation for microscopy

2.3.3.1 Confocal fluorescence microscopy

One day prior imaging cells were seeded on microscopy dishes (µ-Slide 8 Well Glass Bottom (ibidi)) in DMEM without phenol red.

The medium was exchanged prior imaging with 200 µl DMEM without phenol red. The compounds were diluted from DMSO stocks in DMEM without phenol red to a total volume of 200 µl and this solution was added to the cell suspension at the microscope. The final DMSO fraction was 0.5 %.

Compounds were added immediately after image acquisition at t = 0 min.

2.3.3.2 Widefield fluorescence microscopy

One day prior imaging cells were seeded on microscopy dishes (µ-Slide 18 Well Glass Bottom (ibidi)) in DMEM without phenol red.

The medium was exchanged prior imaging with 100 µl DMEM without phenol red. The compounds were diluted from DMSO stocks in DMEM without phenol red to a total volume of 100 µl and this solution was added to the cell suspension at the microscope. The final DMSO fraction was 0.5 %.

Compounds were added immediately after image acquisition at t = 0 min.

2.4 Luciferase transcription assay

The design of the luciferase transcription assay was based on the CheckMate Mammalian Two-Hybrid System (Promega) as in previous publication[2]. 293 FlpIN TREx cells (Thermo Fisher Scientific Inc.) were seeded on 96-well plates in 200 µl DMEM without phenol red. On the next day, cells were transiently transfected with different transfection mixtures (each 20 µl, see below). Transfection mixtures were prepared with Lipofectamine 3000 Transfection Reagent (Thermo Fisher Scientific) according to the manufacturer's protocol.

Positive control	ABI/PYR^{Mandi}	ABI/PYL
50 ng pGL 4.31 (fLuc, Promega)	50 ng pGL 4.31 (fLuc, Promega)	50 ng pGL 4.31 (fLuc, Promega)
50 ng pACT-MyoD (Promega)	50 ng pVP16AD-PYR^{Mandi}-IRES-GAL4BD-ABI (plasmid from previous publication[2])	50 ng SV-ABAactDA (Addgene plasmid #38247)
50 ng pBIND-Id (Promega)		

One day after transfection, the medium was exchanged by medium (again 200 µl DMEM without phenol red) containing the compounds (Mandi, pMandi, OP, OP-AM or pOP) in different concentrations (each 3 wells per condition, final DMSO fraction ≤ 1 %).

For studying light-induced luciferase expression upon irradiation in the presence of pMandi or pOP irradiation was performed immediately after compound addition. Irradiation was performed with a SOLIS-405C LED (Thorlabs, nominal wavelength 405 nm, bandwidth (FWHM) 14 nm) controlled with a DC2200 driver (Thorlabs) and a photocurrent of 100 mA for Mandi and 1000 mA for pOP. The output power was measured with a power meter at the outlet of the light guide (405 nm, 100 mA: 20 mW; 405 nm, 1000 mA: 217 mW). The light source for irradiation was connected via a light guide to the top of the each well. With this setup, the light guide was so close to the sample, that no loss of light power because of light beam broadening and light not hitting the sample was apparent.

After overnight incubation, the kit Steady-Glo Luciferase Assay System (Promega) was performed and bioluminescence was read out on a Tecan Spark plate reader at 25 °C. Luminescence values were normalized to the mean luminescence values of the positive control on the same plate to obtain the relative luminescence.

For data analysis the following Hill equation was fitted to the relative luminescence values with OriginPro 2021 (v9.8.0.200):

$$y = START + (END - START)\frac{x^n}{k^n + x^n},$$

with the measured relative luminescence y, the maximum asymptote END, the minimum asymptote $START$, the compound concentration x, the EC50 k and the number of cooperative sites n. The determined EC50 values are shown below. For the EC50 values depicted in gray the maximum asymptote END was set to upper plateau of the experiments with Mandi and PYR^{Mandi} or OP and PYL, respectively. A comparison of Mandi with ABA was shown in previous publication.[2]

Compound	Receptor protein	k = EC50 [M]
Mandi	PYR^{Mandi}	(3.93 ± 0.78) 10^{-7}
OP	PYL	(4.50 ± 1.28) 10^{-7}
OP-AM	PYL	(4.30 ± 0.73) 10^{-7}
pMandi	PYR^{Mandi}	(2.04 ± 1.05) 10^{-4}
pOP	PYL	(2.48 ± 0.32) 10^{-5}
Mandi	PYL	(1.12 ± 1.85) 10^{-3}
OP	PYR^{Mandi}	(2.88 ± 4.19) 10^{-4}

Relative luminescence values depicted in figures in this work represent the average from two experiments with each three replicates; the error bars denote the standard deviation of the mean.

2.5 Fluorescence microscopy

2.5.1 Confocal fluorescence microscopy

Confocal fluorescence imaging was performed with a SP8 microscope (Leica) equipped with a White Light Laser (WLL, 470 nm to 670 nm) and a 405 nm diode laser for excitation.

For imaging of eGFP excitation was conducted at 488 nm and the emission was detected from 503 nm to 563 nm on a HyD 2 SMD detector (Leica). mCherry was imaged with excitation at 587 nm and the emission was detected from 607 nm to 667 nm on a HyD 4 SMD detector (Leica). The different channels were imaged simultaneously.

Irradiation for photouncaging was performed immediately after the previous image was acquired.

2.5.1.1 Microscopy settings for imaging of live cells

For live-cell imaging a HC PL APO CS2 20x/0.75 DRY objective was used. The following settings were applied: zoom factor 2.00, line average 4, pixel size 142 nm x 142 nm, format 2048 x 2048 pixels, pixel dwell time 787 ns, pinhole 1.2 airy units (67.94 µm). Irradiation for in cellulo photouncaging of pMandi was conducted at 405 nm with a power of 0.43 mW and normal imaging settings of the whole field of view. Irradiation for in cellulo photouncaging of pOP was conducted at 405 nm with a power of 2.31 mW and normal imaging settings of a defined region of interest (ROI) or the whole field of view. Imaging was performed at 37 °C. The pearson coefficient was calculated with the plugin Colocalization_finder (version 1.6) in Fiji ImageJ using the eGFP and mCherry channels of confocal fluorescence microscopy images.

2.5.1.2 Microscopy settings for imaging of live medaka embryos

For imaging of live medaka embryos a HC PL APO CS2 63x/1.40 OIL objective was used. The following settings were applied: zoom factor 2.00, line average 4, pixel size 90 nm x 90 nm, format 2048 x 2048 pixels, pixel dwell time 787 ns, pinhole 1.2 airy units (114.6 µm). Irradiation for in vivo photouncaging of pMandi was conducted at 405 nm with a power of 1.10 mW and normal imaging settings of a defined region of interest (ROI) or the whole field of view. Imaging was performed at 27 °C.

2.5.2 Widefield fluorescence microscopy

For widefield fluorescence imaging a DMi8 microscope from Leica was used, equipped with 4 LEDs (405, 488, 552 and 638 nm) for excitation and dichroic mirrors (409, 493, 573 and 652 nm) for the detection of the emission. For imaging of eGFP and mCherry excitation prefilters were used (mCherry: 580 nm, eGFP: 490 nm). An HC PL APO 20x/0.80 DRY objective was used. Irradiation for in cellulo photouncaging of pMandi was conducted at 405 nm with a power of 53 mW. Imaging was performed at 37 °C.

Irradiation for photouncaging was performed immediately after the previous image was acquired.

2.6 Medaka embryo experiments

2.6.1 Fish maintenance

Adult medaka (*Oryzias latipes*, Cab strain) stocks were raised and maintained as closed stocks at 28°C on a 14 h:10 h light:dark cycle at Heidelberg University. Fish husbandry and experiments were performed in accordance with the local animal welfare guidelines (Tierschutzgesetz §11, Abs. 1, Nr. 1, husbandry permit number 35-9185.64/BH Wittbrodt). The fish facility is under the supervision of the local representative of the animal welfare agency.

2.6.2 In vitro transcription of mRNA for microinjections

For in vitro transcription of mRNA, first the plasmids pCS2+-eGFP-ABI, pCS2+-Lyn-mCherry-PYR^{Mandi} and pCS2+-Lyn-mCherry-PYL were linearized with HpaI (NEB) according to the manufacturers protocol. Then, the linearized plasmids were purified using the QIAquick PCR Purification Kit (Qiagen) and transcribed in vitro with the mMESSAGE mMACHINE SP6 Transcription Kit (Thermo Fisher Scientific). The mRNA was purified using the RNeasy Mini Kit (Qiagen) and the quality of the mRNA was assessed via agarose gel electrophoresis.

2.6.3 Microinjections

Microinjections were performed in wild-type Cab embryos at the one-cell stage. Fertilized embryos were injected with 50 ng/µl eGFP-ABI and Lyn-mCherry-PYR^{Mandi} mRNA or 50 ng/µl eGFP-ABI and Lyn-mCherry-PYL mRNA. Injected embryos were maintained in embryo rearing medium (1x ERM: 17 mM NaCl, 40 mM KCl, 0.27 mM $CaCl_2 \cdot 2\,H_2O$, 0.66 mM $MgSO_4 \cdot 7\,H_2O$ and 17 mM HEPES) and incubated at 26 °C. Embryos were screened for eGFP and mCherry expression 1 day after injections using a Nikon SMZ18 stereomicroscope. Only fluorescent positive and properly developed embryos were continued with.

2.6.4 Sample preparation for microscopy

Confocal fluorescence imaging was performed 2 days post fertilization (dpf).[3] Embryos were dechorionated using hatching enzyme and washed with 1x ERM. For imaging embryos were mounted dorsally on 12-well glass-bottom plates (Matek) in 200 µl low melting agarose (1 %) supplemented with 1x Tricaine and covered with 1 ml 1x ERM supplemented with 1x Tricaine. The compounds were diluted from DMSO stocks in 1 ml ERM supplemented with 1x Tricaine and the solution was added to the embedded embryos. The final DMSO fraction was 0.5 %.

2.6.5 Toxicity test of OP-AM, pOP, Mandi and pMandi in medaka embryos

To assess the influence of the compounds OP-AM, pOP, Mandi and pMandi in medaka embryos a toxicity test was performed. 10 µM DMSO stock solutions were prepared of each compound and further diluted in 1x ERM to the final concentration. Wild-type Cab embryos were collected and kept at 26 °C. At 2 dpf embryos were transferred into a 96-well plate and treated with 5 µM of each

compound (final DMSO fraction: 0.1 %) and 0.1 % DMSO as well as 1x ERM only as controls. The embryos were phenotyped 3 h, 6 h and 24 h after treatment and after hatch. After 24 h of compound incubation the media was exchanged for 1x ERM and refreshed every 24 h until hatch. Phenotypes were assessed by imaging on a Nikon SMZ18 stereomicroscope. Result of the toxicity test are shown in Figure S40.

2.7 Chemical synthesis and characterization

2.7.1 General experimental conditions and analytical methods

2.7.1.1 Chemicals and reagents

The used chemicals were obtained from commercial vendors (Sigma-Aldrich, Carl Roth GmbH + Co. KG, TCI Deutschland GmbH, Merck KGaA, Acros Organics or abcr GmbH) and used without further purification. Anhydrous solvents were stored over molecular sieve. Water was purified prior usage with the Milli-Q system (Merck KGaA).

2.7.1.2 Synthesis conditions

All reactions with water or oxygen sensitive reagents or intermediates were performed under argon atmosphere and in anhydrous solvents. Standard Schlenk-techniques were used.

2.7.1.3 Chromatography

For reaction control and analysis thin layer chromatography (TLC) was performed using polyester plates with silica gel 60 and fluorescence indicator (Macherey-Nagel POLYGRAM SIL G/UV_{254}). Compound were visualized with UV light (254 nm or 365 nm) or common staining reagents. Moreover, liquid chromatography – mass spectrometry (LC-MS) was conducted on a LCMS-2020 system from Shimadzu (acetonitrile:water gradient + 0.1 % formic acid) equipped with a C18 column from Supelco with the size 1.9 µm, 2.1 x 50 mm and a SPD-20AV UV-VIS photodiode array detector for product visualization.

Column chromatography was performed on the flash column chromatography Isolera One from Biotage with SiliaSep columns (silica gel 40 µm to 63 µm) from Silicycle.

For preparative high performance liquid chromatography (HPLC) the system Alliance e2695 (Waters) or the system UltiMate 3000 (Thermo Fisher Scientific Inc.) was used. Both systems were connected to C18 columns from Supelco with the sizes 5 µm, 10 x 250 mm (flow rate 4 ml min^{-1}) or 5 µm, 21.2 x 250 mm (flow rate 8 ml min^{-1}). Fractions were collected with an automated fraction collector based on the absorption maxima of the purified compounds.

2.7.1.4 Nuclear magnetic resonance spectroscopy

Nuclear magnetic resonance (NMR) spectra were recorded on Bruker Avance III HD spectrometer system equipped with a Bruker AscendTM 400 magnet (field strength: 9.4 T, ^{1}H-NMR frequency: 400.15 MHz, ^{13}C{^{1}H}-NMR frequency: 100.62 MHz) at 298.15 K. Chemical shifts δ are reported in ppm, internally referenced to the residual ^{1}H-NMR signal of the solvent (e.g. chloroform at δ = 7.26 ppm, methanol at δ = 3.31 ppm) in ^{1}H-NMR spectra and to the ^{13}C-isotopes of the solvent (e.g. deuterated chloroform at δ = 77.16 ppm, deuterated methanol at δ = 49.00 ppm, deuterated DMSO at δ = 39.52 ppm) in ^{13}C-NMR spectra.[4] The multiplicity is given with the following abbreviations: s = singlet, d = doublet, t = triplet, q = quartet, qui = quintet, dd = doublet of doublet, br s = broad singlet etc. coupling constants *J* are given in Hz. NMR spectra were analyzed with the software MestReNova (v14.1.0-24037).

2.7.1.5 Mass spectrometry

Mass spectrometry (MS) measurements were performed with electrospray ionisation (ESI) in positive (+) or in negative (-) mode by the mass spectrometry core facility of the Max Planck Institute for Medical Research Heidelberg on a Bruker maXis II™ ETD mass spectrometer. As solvent acetonitrile was used.

2.7.2 Purchased reagents ABA and Mandi

(+)-Abscisic acid (ABA, **6**) and mandipropamid (Mandi, **1**) were purchased from Sigma-Aldrich as analytical standard and used without further purification.

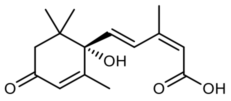

ABA (**6**)

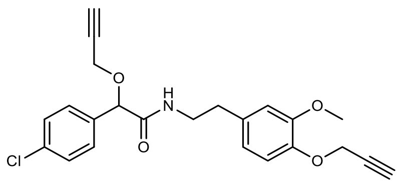

Mandi (**1**)

2.7.3 Synthesis of ABA-AM

(+)-Abscisic acid acetoxymethyl ester (7)

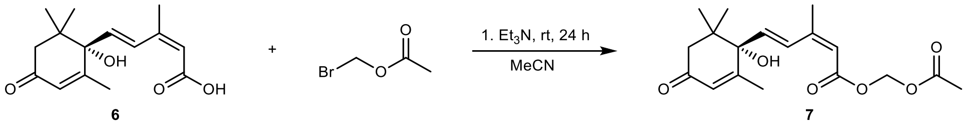

(+)-Abscisic acid acetoxymethyl ester (**7**) was prepared according to procedures described in previous publications[2]:

(+)-Abscisic acid (**6**) (10 mg, 37.8 µmol, 1.00 eq) was dissolved in dry acetonitrile (1 ml). Then bromomethyl acetate (4.82 µl, 7.52 mg, 49.2 µmol, 1.30 eq) and triethylamine (6.84 µl, 4.98 mg, 49.2 µmol, 1.30 eq) were added and the reaction mixture was stirred for 24 h at room temperature. The solvent was removed in vacuo and the crude product was purified by preparative reverse phase HPLC (C18, acetonitrile:water gradient + 0.1 % TFA). The product was obtained as colorless solid (9.60 mg, 28.5 µmol, 76 %).

^{1}H-NMR (400.15 MHz, CD_3OD): δ = 7.80 (d, J = 16.1 Hz, 1H), 6.35 (d, J = 16.1 Hz, 1H), 5.94 (s, 1H), 5.77 (s, 1H), 5.75 (s, 2H), 2.54 (d, J = 17.0 Hz, 1H), 2.20 (d, J = 16.9 Hz, 1H), 2.08 (s, 6H), 1.94 (d, J = 1.3 Hz, 3H), 1.07 (s, 3H), 1.03 (s, 3H) ppm.

^{13}C-NMR (100.62 MHz, $(CD_3)_2SO$): δ = 197.2, 169.4, 163.6, 162.8, 153.7, 139.7, 126.8, 126.1, 115.3, 78.7, 78.4, 49.3, 41.3, 24.2, 23.2, 21.0, 20.5, 18.8 ppm.

HRMS (ESI(+), CH_3CN): m/z 337.1650 $[M+H]^+$, calculated for $C_{18}H_{25}O_6^+$: m/z 337.1646.

2.7.4 Synthesis of pMandi

pMandi (**2**) was synthesized according to the following synthetic procedure.

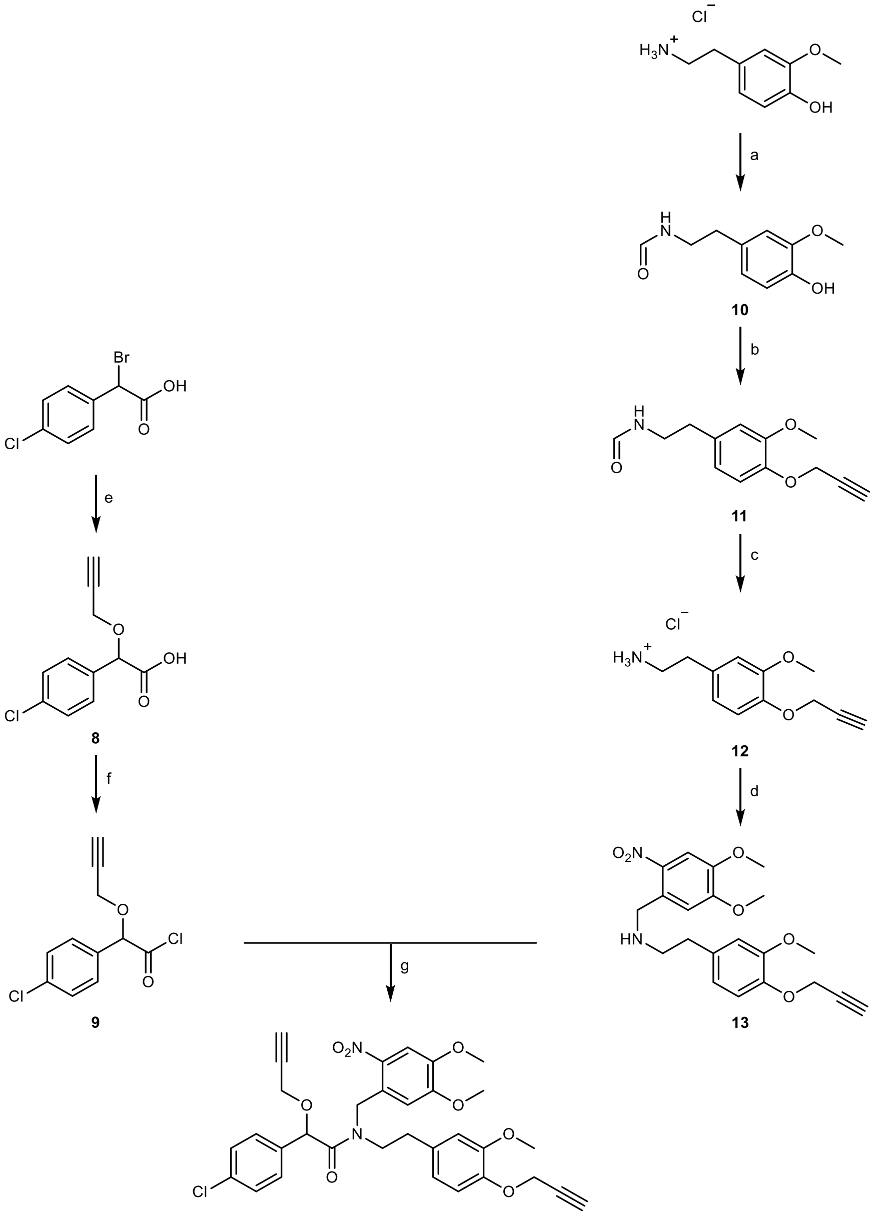

pMandi (**2**)

Scheme S1: Route for synthesis of pMandi (**2**). a: 2-propyn-1-ol, KOH, rt, 2 h; b: 1. oxalyl chloride, DMF (cat.), 0 °C; 2. rt, 2 h; c: Ac_2O, formic acid, 70 °C, 2 h; d: 3-bromoprop-1-yne, NaOMe, 65 °C,4 h; e: HCl, rt, 91 h; f: 1. 4,5-dimethoxy-2-nitrobenzaldehyd, $NaBH(OAc)_3$, rt; 2. 50 °C, 14 h; g: DIPEA, 0 °C -> rt, 21 h.

4-Chloro-α-(2-propyn-1-yloxy)benzeneacetic acid (8)

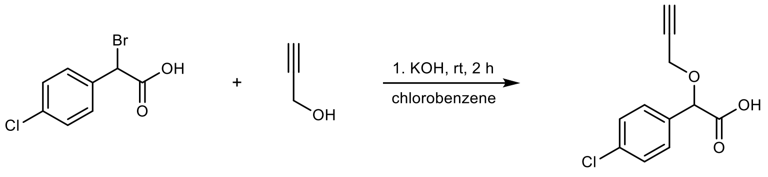

This synthesis was conducted according to literature[5] with slight modifications:

Potassium hydroxide (1.32 g, 24.0 mmol, 3.00 eq) and 2-propyn-1-ol (0.95 ml, 899 mg, 16.0 mmol, 2.00 eq) were suspended in chlorobenzene (6 ml). Then, a solution of α-bromo-4-chlorobenzeneacetic acid (2.00 g, 8.02 mmol, 1.00 eq) in chlorobenzene (8 ml) was added dropwise. The reaction mixture was stirred for 2 h at room temperature. Hydrochloric acid (1 mol l^{-1}, 20 ml) was added and the layers were separated. The aqueous layer was extracted with dichloromethane (3 x 50 ml) and the combined organic layer was dried over sodium sulphate. The solvent was removed under reduced pressure and the obtained residue was dried in vacuo. The product was obtained as slightly yellow solid (1.62 g, 7.20 mmol, 90 %).

^{1}H-NMR (400.15 MHz, $CDCl_3$): δ = 7.41-7.36 (m, 4H), 5.21 (s, 1H), 4.36-4.11 (m, 2H), 2.52 (s, 1H) ppm.

^{13}C-NMR (100.62 MHz, $CDCl_3$): δ = 173.5, 135.5, 133.1, 129.3, 129.1, 77.8, 76.6, 56.6 ppm; one signal superimposed by solvent peak.

HRMS (ESI(-), CH_3CN): *m/z* 223.0166 $[M-H]^-$, calculated for $C_{11}H_8O_3Cl^-$: *m/z* 223.0156.

4-Chloro-α-(2-propyn-1-yloxy)benzeneacetyl chloride (9)

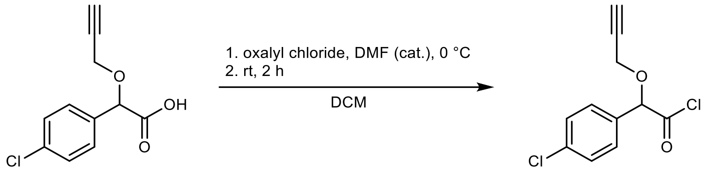

4-Chloro-α-(2-propyn-1-yloxy)benzeneacetic acid (**8**) (50.0 mg, 223 µmol, 1.00 eq) was dried in vacuo for 30 min and subsequently dissolved in dry dichloromethane (1 ml). Then, a drop of dry dimethylformamide (1.72 µl, 1.63 mg, 22.3 µmol, 0.10 eq) was added. At 0 °C, a solution of oxalyl chloride (56.5 mg, 445.2 µmol, 2.00 eq) in dichloromethane (2 mol l^{-1}, 233 µl) was added dropwise and the reaction mixture was stirred for 15 min at 0 °C and further 2 h at room temperature. The solvent was removed under reduced pressure. The obtained product (54.1 mg, 223 µmol, 100 %) was used in the next synthetic step without further purification with the assumption of complete conversion.

N-[2-(4-Hydroxy-3-methoxyphenyl)ethyl]formamide (10)

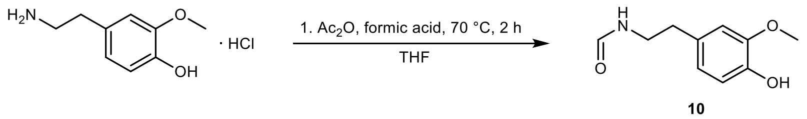

This synthesis was conducted according to literature[6] with slight modifications:

Acetic anhydride (3.46 ml, 3.76 g, 36.8 mmol, 15.0 eq) was cooled to 0 °C and formic acid (1.85 ml, 2.26 g, 49.1 mmol, 20.0 eq) was added dropwise. The mixture was stirred for 2 h at 55 °C and subsequently cooled to 0 °C again. Then, tetrahydrofuran (5 ml) and 4-(2-aminoethyl)-2-methoxyphenol hydrochloride (500 mg, 2.45 mmol, 1.00 eq) were added and the reaction mixture was stirred for 2 h at 70 °C. The solvent was removed in vacuo and the crude product was purified by column chromatography (silica gel, dichloromethane:ethyl acetate gradient). The product was obtained in mixture with side products as slightly yellow liquid (444 mg, 1.89 mmol, 77 %) and used in the next synthetic step without further purification.

^{1}H-NMR (400.15 MHz, $CDCl_3$): δ = 8.08 (d, *J* = 6.8 Hz, 1H), 6.96-6.67 (m, 3H), 5.86-5.64 (m, 1H), 3.87-3.81 (m, 3H), 3.57-3.52 (m, 2H), 2.84-2.74 (m, 2H) ppm; (all signals superimposed by signals of side products).

HRMS (ESI(+), CH_3CN): *m/z* 196.0968 [M+H]$^+$, calculated for $C_{10}H_{14}NO_3^+$: *m/z* 196.0968.

The following side products could be identified:

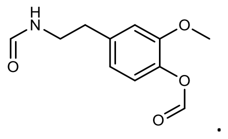

HRMS (ESI(+), CH_3CN): *m/z* 224.0916 [M+H]$^+$, calculated for $C_{11}H_{14}NO_4^+$: *m/z* 224.0917.

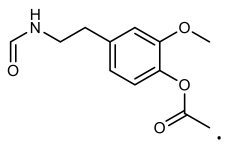

HRMS (ESI(+), CH_3CN): *m/z* 238.1071 [M+H]$^+$, calculated for $C_{12}H_{16}NO_4^+$: *m/z* 238.1074.

N-[2-[3-Methoxy-4-(2-propyn-1-yloxy)phenyl]ethyl]formamide (11)

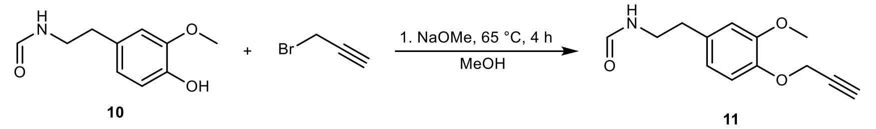

This synthesis was conducted according to literature[6]:

N-[2-(4-Hydroxy-3-methoxyphenyl)ethyl]formamide (**10**) (227 mg, 1.42 mmol, 1.00 eq) was dissolved in dry methanol (3.3 ml). Then, a solution of sodium methoxide (76.5 mg, 1.42 mmol, 1.00 eq) in methanol (25 wt%, 4.37 mol l^{-1}, 324 µl) and a solution of 3-bromoprop-1-yne (168 mg, 1.42 mmol, 1.00 eq) in xylene (80 wt%, 157 µl) were added. The reaction mixture was stirred for 4 h at 65 °C. The solvent was removed in vacuo and ethyl acetate (10 ml) was added to the residue. The organic layer was washed with water (2 x 10 ml) and dried over sodium sulphate. The solvent was removed under reduced pressure and the crude product was purified by column chromatography (silica gel, dichloromethane:ethyl acetate gradient). The product was obtained as slightly yellow liquid (142 mg, 609 µmol, 43 %).

^{1}H-NMR (400.15 MHz, $CDCl_3$): δ = 8.15 (s, 1H), 6.98 (d, *J* = 8.6 Hz, 1H), 6.74-6.70 (m, 2H), 5.55 (s, 1H), 4.74 (d, *J* = 2.4 Hz, 2H), 3.87 (s, 3H), 3.57 (q, *J* = 6.7 Hz, 2H), 2.80 (t, *J* = 6.9 Hz, 2H), 2.50 (t, *J* = 2.4 Hz, 1H) ppm.

^{13}C-NMR (100.62 MHz, $CDCl_3$): δ = 161.3, 150.0, 145.7, 132.6, 120.7, 114.9, 112.5, 78.8, 75.9, 57.0, 56.1, 39.4, 35.3 ppm.

HRMS (ESI(+), CH_3CN): *m/z* 234.1125 [M+H]$^+$, calculated for $C_{13}H_{16}NO_3^+$: *m/z* 234.1125.

3-Methoxy-4-(2-propyn-1-yloxy)benzeneethanamine hydrochloride (12)

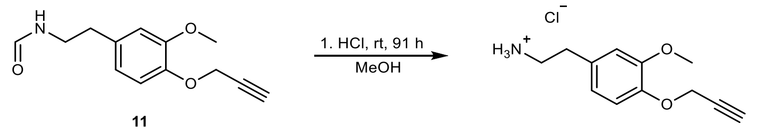

This synthesis was conducted according to literature[7] with slight modifications:

N-[2-[3-Methoxy-4-(2-propyn-1-yloxy)phenyl]ethyl]formamide (**11**) (562 mg, 2.41 mmol, 1.00 eq) was dissolved in dry methanol (15 ml) and a solution of hydrogen chloride (264 mg, 1.83 mmol, 3.00 eq) in methanol (1.25 mol l^{-1}, 5.78 ml) was added. The reaction mixture was stirred for 91 h at room temperature. The solvent was removed under reduced pressure and the residue was dissolved in methanol (5 ml). Then, diethyl ether (20 ml) was added resulting in the formation of a precipitate. The solvent was pipetted off and the residue was died in vacuo. The product was obtained as slightly brown solid (415 mg, 1.72 mmol, 71 %).

^{1}H-NMR (400.15 MHz, CD_3OD): δ = 7.03 (d, *J* = 8.2 Hz, 1H), 6.91 (d, *J* = 2.0 Hz, 1H), 6.81 (dd, *J* = 8.2 Hz, *J* = 2.0 Hz, 1H), 4.73 (d, *J* = 2.4 Hz, 2H), 4.58 (s, 3H), 3.85 (s, 3H), 3.16 (t, *J* = 7.6 Hz, 2H), 2.93-2.88 (m, 3H) ppm.

^{13}C-NMR (100.62 MHz, CD_3OD): δ = 151.7, 147.6, 132.0, 122.0, 116.9, 114.0, 79.8, 76.9, 57.9, 56.5, 42.0, 34.3 ppm.

HRMS (ESI(+), CH_3CN): *m/z* 206.1174 [M+H]$^+$, calculated for $C_{12}H_{16}NO_2^+$: *m/z* 206.1176.

N-(4,5-Dimethoxy-2-nitrobenzyl)-2-(3-methoxy-4-(prop-2-yn-1-yloxy)phenyl)ethan-1-amine (13)

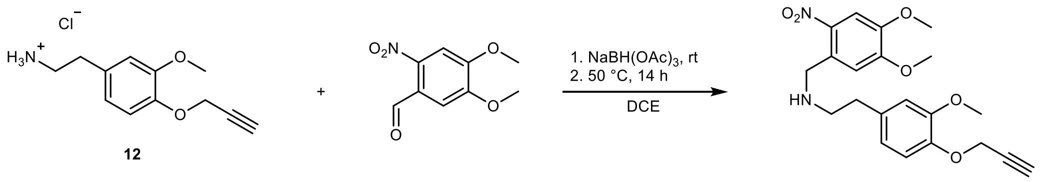

3-Methoxy-4-(2-propyn-1-yloxy)benzeneethanamine hydrochloride (**12**) (50.0 mg, 207 µmol, 1.00 eq) was dissolved in dry 1,2-dichloroethane (2 ml). Then, *N*,*N*-diisopropylethylamine (DIPEA) (103 µl, 80.2 mg, 621 µmol, 3.00 eq) and 4,5-dimethoxy-2-nitrobenzaldehyd (80 %, 54.6 mg, 207 µmol, 1.00 eq) were added. The yellow suspension was stirred at room temperature for 15 min and sodium triacetoxyborohydride (92.3 mg, 414 µmol, 2.00 eq) was added. The reaction mixture was stirred for 14 h at 50 °C. A saturated aqueous solution of sodium hydrogencarbonate (5 ml) was added and the resulting mixture was extracted with dichlormethane (3 x 10 ml). The combined organic layer was washed with brine (10 ml) and dried over sodium sulphate. The solvent was removed under reduced pressure and the crude product was purified by preparative reverse phase HPLC (C18, acetonitrile:water gradient + 0.1 % TFA). The product was obtained as brown solid (21.4 mg, 53.4 µmol, 26 %).

^{1}H-NMR (400.15 MHz, $CDCl_3$): δ = 7.56 (s, 1H), 7.14 (s, 1H), 6.99 (d, *J* = 8.5 Hz, 1H), 6.77-6.75 (m, 2H), 4.74 (d, *J* = 2.4 Hz, 2H), 4.35 (s, 2H), 3.94 (s, 3H), 3.87 (s, 3H), 3.86 (s, 3H), 3.40 (t, *J* = 7.5 Hz, 2H), 2.95 (t, *J* = 7.4 Hz, 2H), 2.51 (t, *J* = 2.4 Hz, 1H) ppm.

^{13}C-NMR (100.62 MHz, $CDCl_3$): δ = 154.2, 150.4, 150.2, 146.3, 141.6, 129.4, 120.7, 120.4, 115.5, 115.1, 112.4, 108.4, 78.5, 76.1, 57.0, 56.8, 56.6, 56.1, 49.7, 49.4, 32.2 ppm.

HRMS (ESI(+), CH_3CN): *m/z* 401.1704 $[M+H]^+$, calculated for $C_{21}H_{25}N_2O_6^+$: *m/z* 401.1707.

pMandi (2)

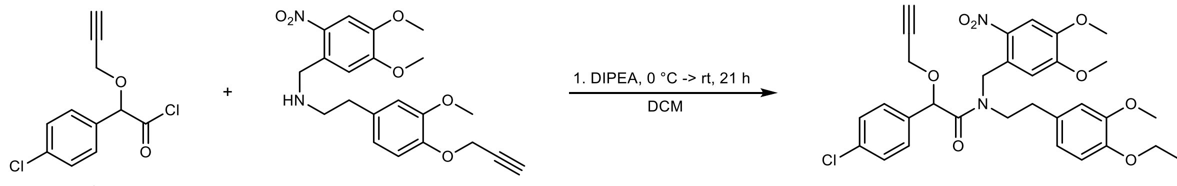

N-(4,5-Dimethoxy-2-nitrobenzyl)-2-(3-methoxy-4-(prop-2-yn-1-yloxy)phenyl)ethan-1-amine (**13**) (21.4 mg, 53.4 µmol, 1.00 eq) was dissolved in dry dichloromethane (1 ml) and *N*,*N*-diisopropylethylamine (DIPEA) (26.5 µl, 20.7 mg, 160 µmol, 3.00 eq) was added. At 0 °C, a solution of 4-Chloro-α-(2-propyn-1-yloxy)benzeneacetyl chloride (**9**) (13.0 mg, 53.4 µmol, 1.00 eq) in dry dichloromethane (1 ml) was added dropwise. The reaction mixture was stirred for 21 h at room temperature. Then, hydrochloric acid (1 mol l^{-1}, 1 ml) and dichloromethane (5 ml) were added and the layers were separated. The organic layer was washed a with saturated, aqueous solution of sodium hydrogencarbonate (5 ml) and dried over sodium sulphate. The solvent was removed under reduced pressure and the crude product was purified by preparative reverse phase HPLC (C18, acetonitrile:water gradient + 0.1 % TFA). The product was obtained as slightly yellow solid (6.9 mg, 11.4 µmol, 21 %).

The NMR spectra show the signals of a 1:1 mixture of the two rotamers.

^{1}H-NMR (400.15 MHz, $CDCl_3$): δ = 7.72 (s, 1H), 7.64 (s, 1H), 7.34 (d, J = 2.4 Hz, 4H), 7.26 (d, J = 1.8 Hz, 4H), 6.99 (d, J = 8.1 Hz, 1H), 6.92 (d, J = 8.1 Hz, 1H), 6.73-6.66 (m, 5H), 6.42 (s, 1H), 5.35 (s, 1H), 5.30 (s, 1H), 5.07 (d, J = 1.9 Hz, 2H), 4.95 (s, 2H), 4.76 (d, J = 2.4 Hz, 2H), 4.73 (d, J = 2.4 Hz, 2H), 4.29-3.98 (m, 4H), 3.96 (s, 3H), 3.93 (s, 3H), 3.86 (s, 3H), 3.82 (s, 3H), 3.78 (s, 3H), 3.71 (s, 3H), 3.67-3.56 (m, 2H), 3.54-3.43 (m, 2H), 2.84 (dd, J = 11.9 Hz, J = 8.1 Hz, 4H), 2.53 (t, J = 2.4 Hz, 1H), 2.51-2.49 (m, 2H), 2.33 (t, J = 2.4 Hz, 1H) ppm.

^{13}C-NMR (100.62 MHz, $CDCl_3$): δ = 170.5, 170.1, 154.1, 153.9, 150.1, 149.9, 148.2, 148.1, 146.0, 145.7, 141.0, 139.9, 135.4, 135.3, 133.8, 133.3, 132.3, 131.7, 129.6, 129.4, 129.3, 129.2, 127.9, 127.7, 120.8, 120.8, 114.9, 114.8, 112.8, 112.7, 110.1, 109.0, 108.8, 108.4, 78.8, 78.8, 78.7, 78.2, 76.3, 76.2, 76.0, 75.9, 57.0, 57.0, 56.7, 56.5, 56.4, 56.3, 56.3, 56.2, 56.0, 49.9, 49.4, 46.1, 34.8, 33.3 ppm.

HRMS (ESI(+), CH_3CN): *m/z* 607.1836 $[M+H]^+$, calculated for $C_{32}H_{32}N_2O_8Cl^+$: *m/z* 607.1842.

2.7.5 Synthesis of OP, pOP and OP-AM

Opabactin (OP, **3**) was synthesized according to literature[8] and further modified to pOP and OP-AM.

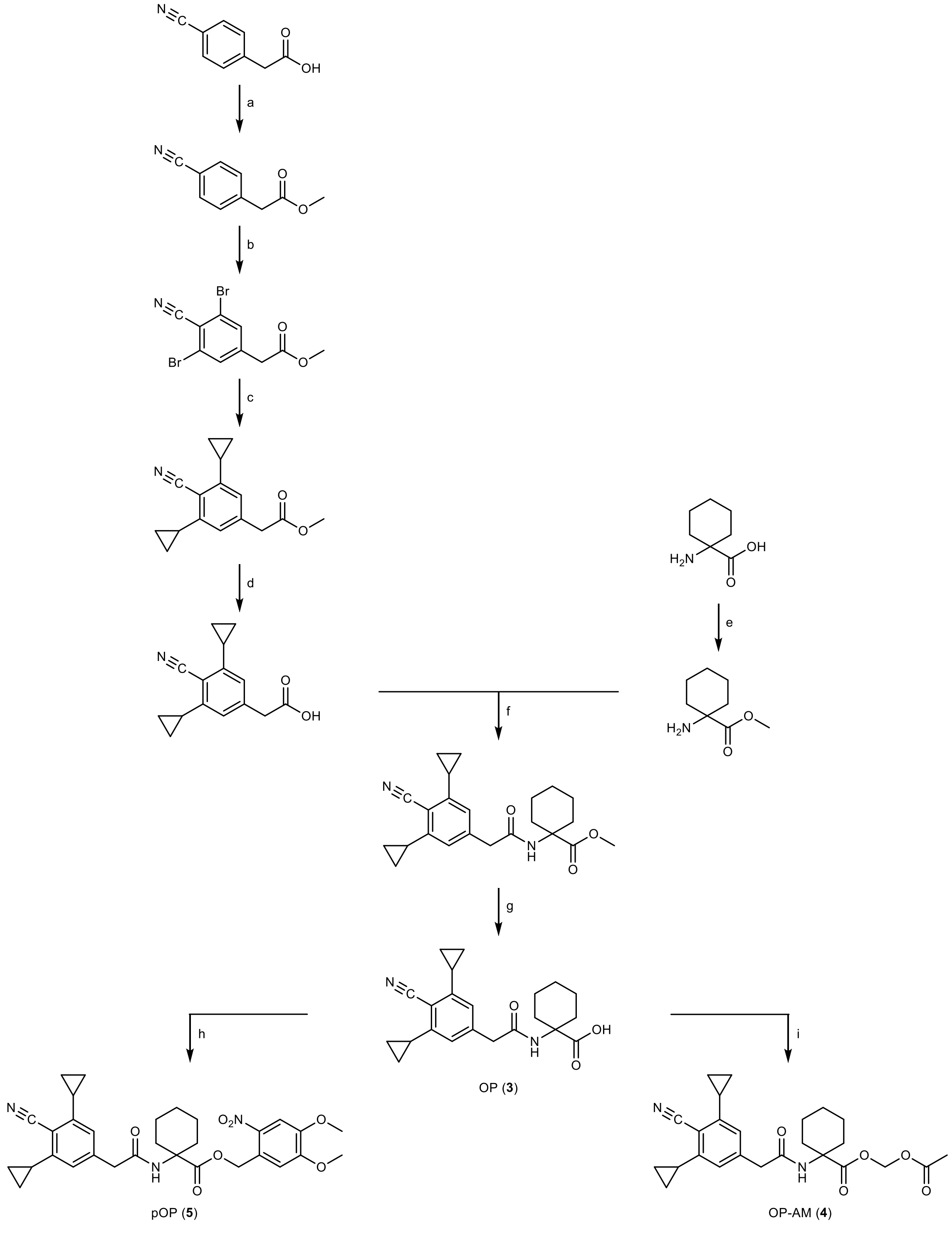

Scheme S2: Route for synthesis of pOP (**5**) and OP-AM (**4**). a: MeOH, H_2SO_4, 0 °C -> rt, 2 h; b: NBS, pTsOH, $Pd(OAc)_2$, 70 °C, 21 h; c: cyclopropylboronic acid, K_3PO_4, $P(Cy)_3$, $Pd(OAc)_2$, 110 °C, 3 h; d: LiOH, rt, 16 h; e: thionyl chloride, 0 °C -> rt, 72 h; f: EDC, DMAP, rt, 16 h; g: LiOH, 50 °C, 4 h; h: 1-(bromomethyl)-2-nitro-4,5-dimethoxybenzene, DIPEA, rt, 72 bromomethyl acetate, DIPEA, rt, 72 h.

OP (3)

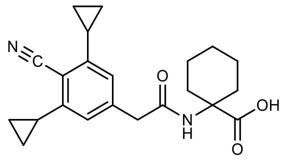

3

Opabactin (OP, **3**) was synthesized according to literature[8] and purified by preparative reverse phase HPLC (C18, acetonitrile:water gradient + 0.1 % TFA).

^{1}H-NMR (400.15 MHz, CD_3OD): δ = 8.15 (s, 1H), 6.79 (s, 2H), 3.51 (s, 2H), 2.26-2.20 (m, 2H), 2.08-2.03 (m, 2H), 1.84-1.76 (m, 2H), 1.64-1.58 (m, 2H), 1.50-1.29 (m, 4H), 1.13-1.08 (m, 4H), 0.83-0.79 (m, 4H) ppm.

^{13}C-NMR (100.62 MHz, CD_3OD): δ = 177.7, 172.6, 149.4, 142.8, 123.3, 118.4, 112.9, 60.3, 60.3, 43.7, 43.7, 33.2, 33.2, 26.4, 22.5, 15.1, 9.7 ppm.

HRMS (ESI(+), CH_3CN): *m/z* 367.2015 $[M+H]^+$, calculated for $C_{22}H_{27}N_2O_3^+$: *m/z* 367.2016.

pOP (5)

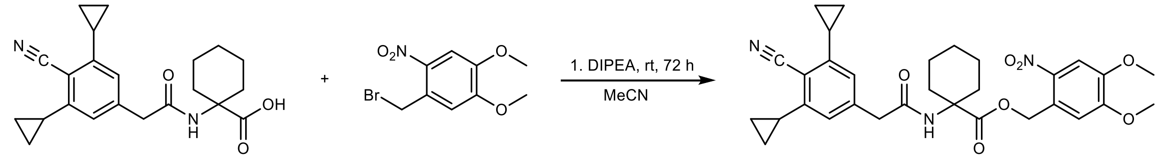

3 5

Opabactin (OP, **3**) (25.0 mg, 68.2 µmol, 1.00 eq) was dissolved in dry acetonitrile (6 ml). Then, *N*,*N*-diisopropylethylamine (DIPEA) (12.3 mg, 15.8 µl, 95.5 µmol, 1.40 eq) and 1-(bromomethyl)-2-nitro-4,5-dimethoxybenzene (26.4 mg, 95.5 µmol, 1.40 eq) were added and the reaction mixture was stirred for 72 h at room temperature. The solvent was removed in vacuo and the crude product was purified by preparative reverse phase HPLC (C18, acetonitrile:water gradient + 0.1 % TFA). The product was obtained as an off-white solid (10.0 mg, 17.8 µmol, 26 %).

^{1}H-NMR (400.15 MHz, $CDCl_3$): δ = 7.70 (s, 1H), 7.06 (s, 1H), 6.61 (s, 2H), 5.56 (s, 2H), 5.54 (s, 1H), 3.97 (s, 3H), 3.96 (s, 3H), 3.49 (s, 2H), 2.30-2.23 (m, 2H), 2.07-2.03 (m, 2H), 1.90-1.83 (m, 2H), 1.68-1.59 (m, 2H), 1.32-1.19 (m, 4H), 1.14-1.09 (m, 4H), 0.75-0.71 (m, 4H) ppm.

^{13}C-NMR (100.62 MHz, $CDCl_3$): δ = 173.7, 169.8, 154.0, 148.9, 148.4, 139.8, 139.7, 127.5, 122.4, 117.5, 113.3, 110.9, 108.2, 64.5, 59.5, 56.9, 56.6, 43.9, 32.4, 25.1, 21.6, 14.5, 9.6 ppm.

HRMS (ESI(+), CH_3CN): *m/z* 562.2549 $[M+H]^+$, calculated for $C_{31}H_{36}N_3O_7^+$: *m/z* 562.2548.

OP-AM (4)

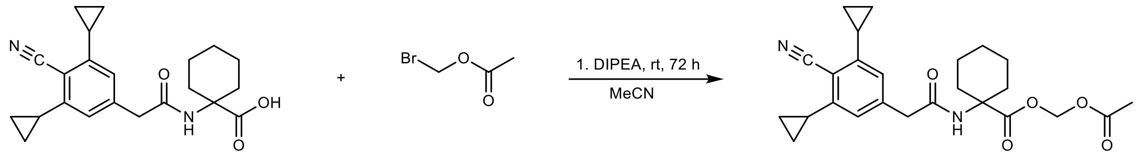

Opabactin (OP, **3**) (25.0 mg, 68.2 µmol, 1.00 eq) was dissolved in dry acetonitrile (4 ml) Then, *N*,*N*-diisopropylethylamine (DIPEA) (12.3 mg, 15.8 µl, 95.5 µmol, 1.40 eq) and bromomethyl acetate (14.6 mg, 9.37 µl, 95.5 µmol, 1.40 eq) were added and the reaction mixture was stirred for 72 h at room temperature. The solvent was removed in vacuo and the crude product was purified by preparative reverse phase HPLC (C18, acetonitrile:water gradient + 0.1 % TFA). The product was obtained as a colorless solid (8.0 mg, 18.2 µmol, 27 %).

^{1}H-NMR (400.15 MHz, CD_3OD): δ = 8.29 (s, 1H), 6.77 (s, 2H), 5.65 (s, 2H), 3.48 (s, 2H), 2.27-2.20 (m, 2H), 1.99-1.94 (m, 5H), 1.84-1.77 (m, 2H), 1.64-1.58 (m, 2H), 1.52-1.29 (m, 4H), 1.14-1.09 (m, 4H), 0.84-0.80 (m, 4H) ppm.

^{13}C-NMR (100.62 MHz, CD_3OD): δ = 174.2, 172.4, 171.1, 149.4, 142.5, 123.5, 118.4, 113.1, 80.6, 60.1, 43.4, 33.1, 26.2, 22.4, 20.5, 15.1, 9.7 ppm.

HRMS (ESI(+), CH_3CN): *m/z* 439.2227 $[M+H]^+$, calculated for $C_{25}H_{31}N_2O_5^+$: *m/z* 439.2227.

2.7.6 NMR spectra

N-(4,5-Dimethoxy-2-nitrobenzyl)-2-(3-methoxy-4-(prop-2-yn-1-yloxy)phenyl)ethan-1-amine (**13**)

^{1}H-NMR:

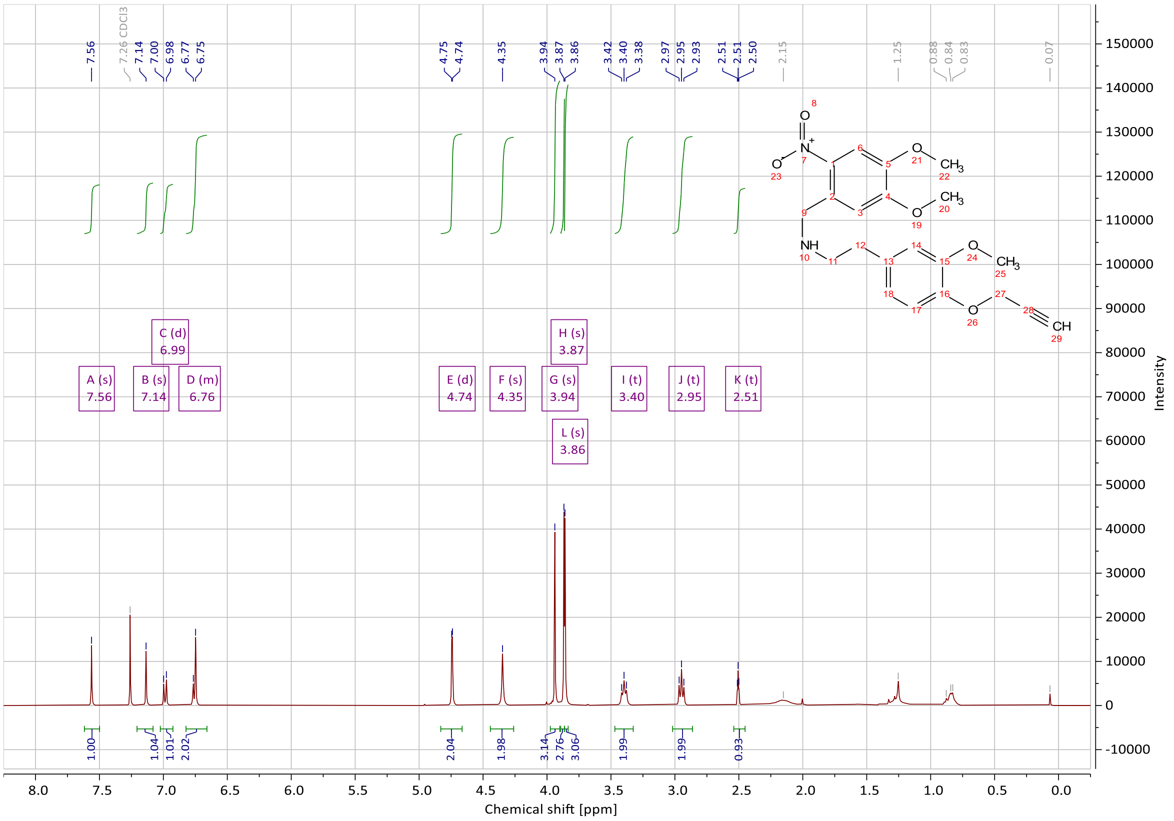

^{13}C-NMR:

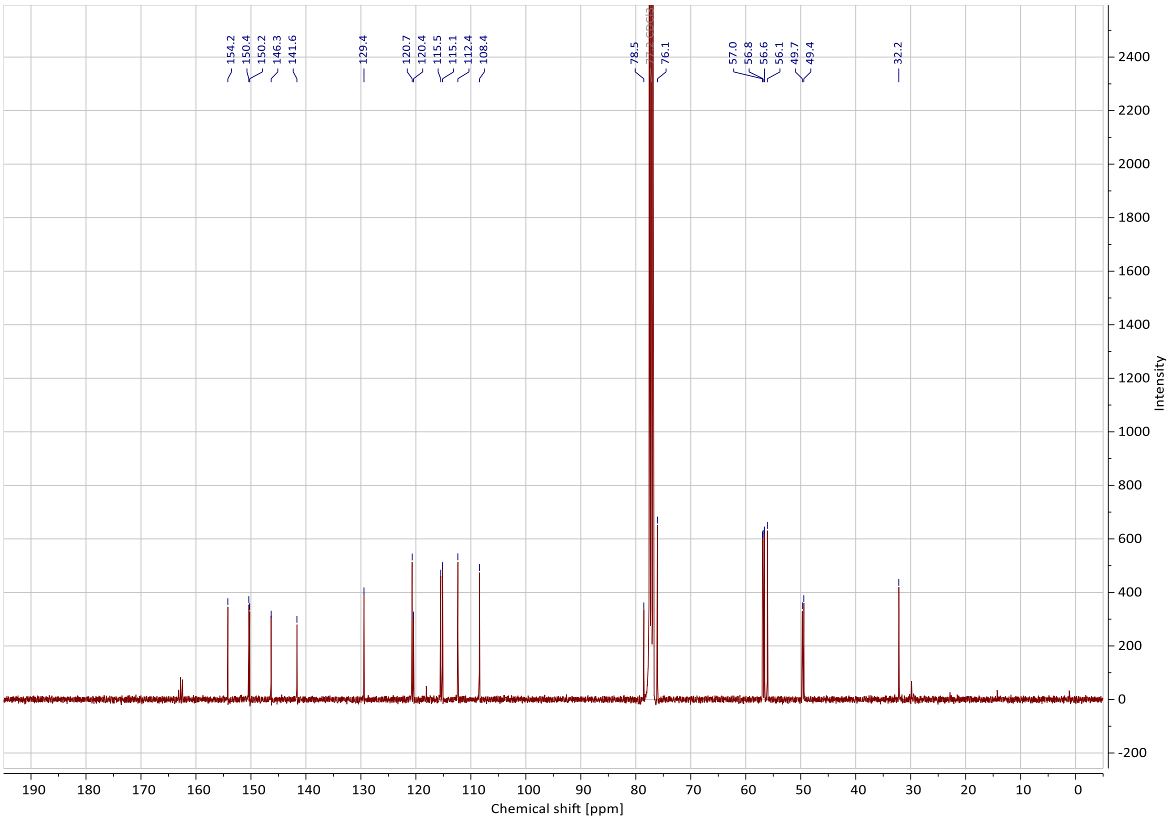

pMandi (**2**)

^{1}H-NMR:

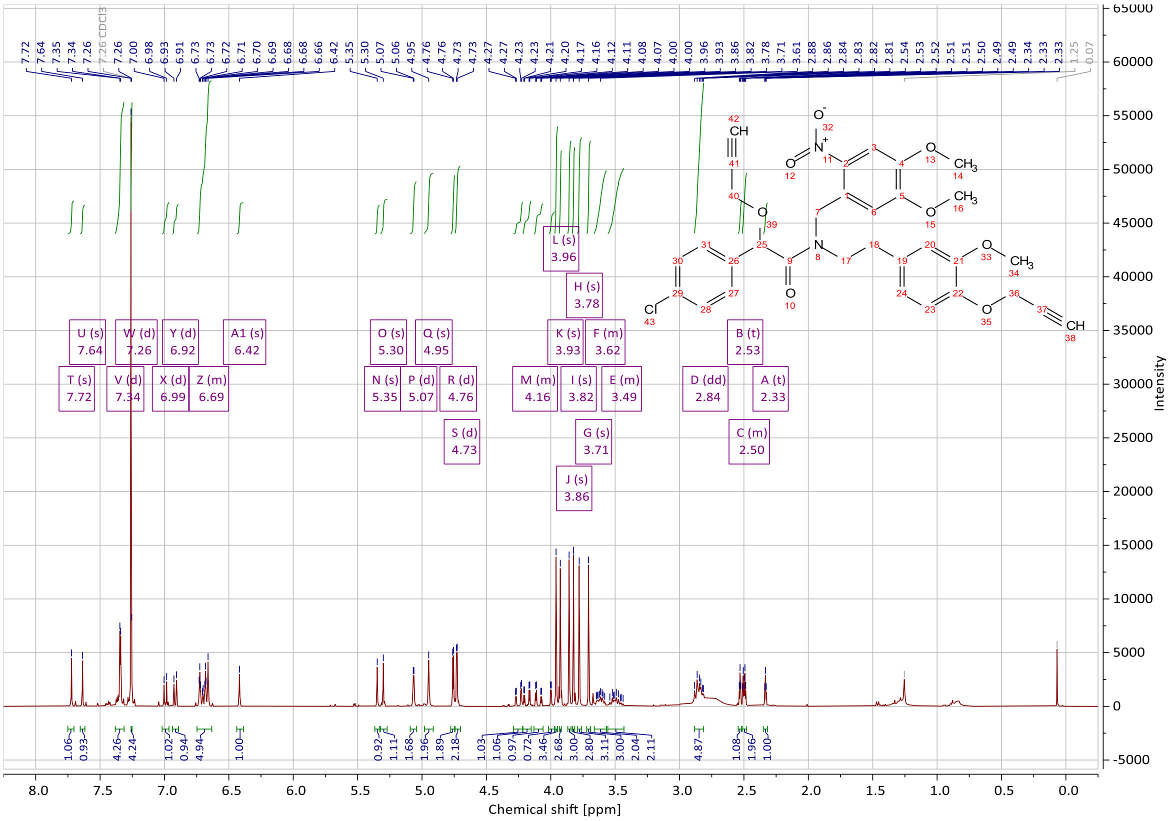

^{13}C-NMR:

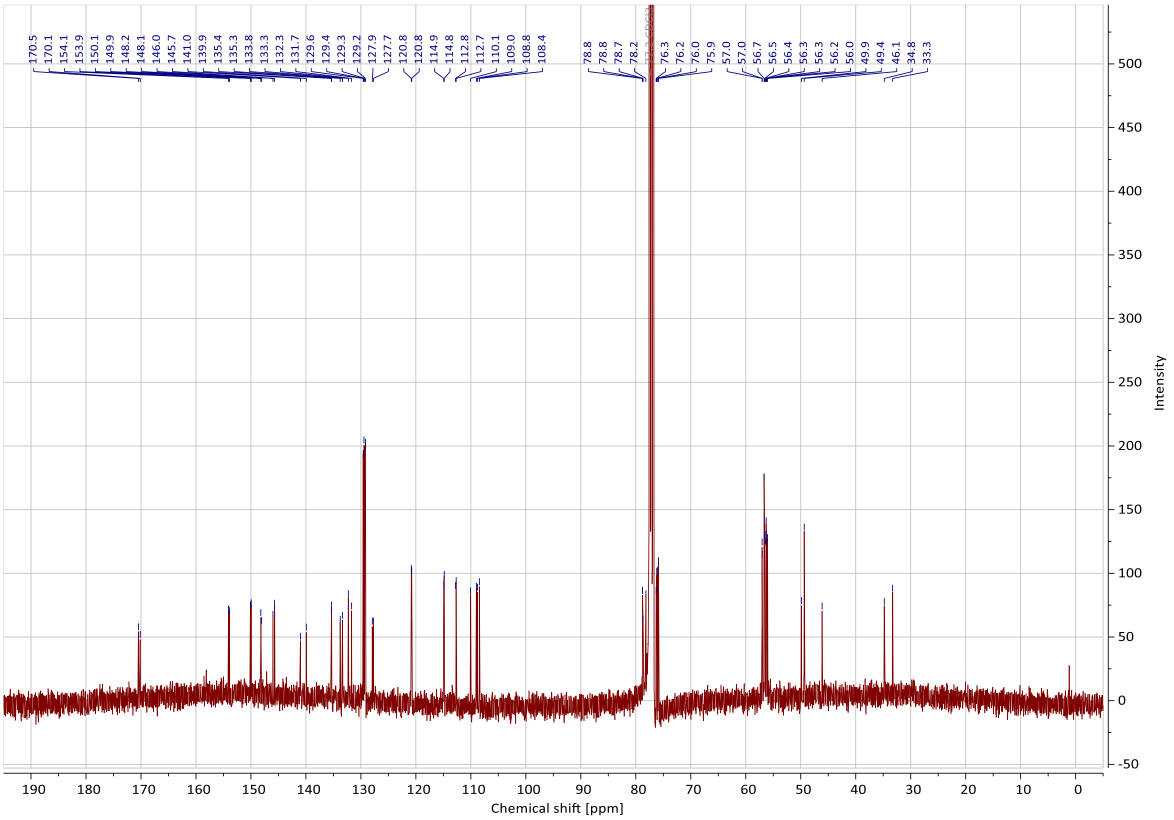

OP (**3**)

^{1}H-NMR:

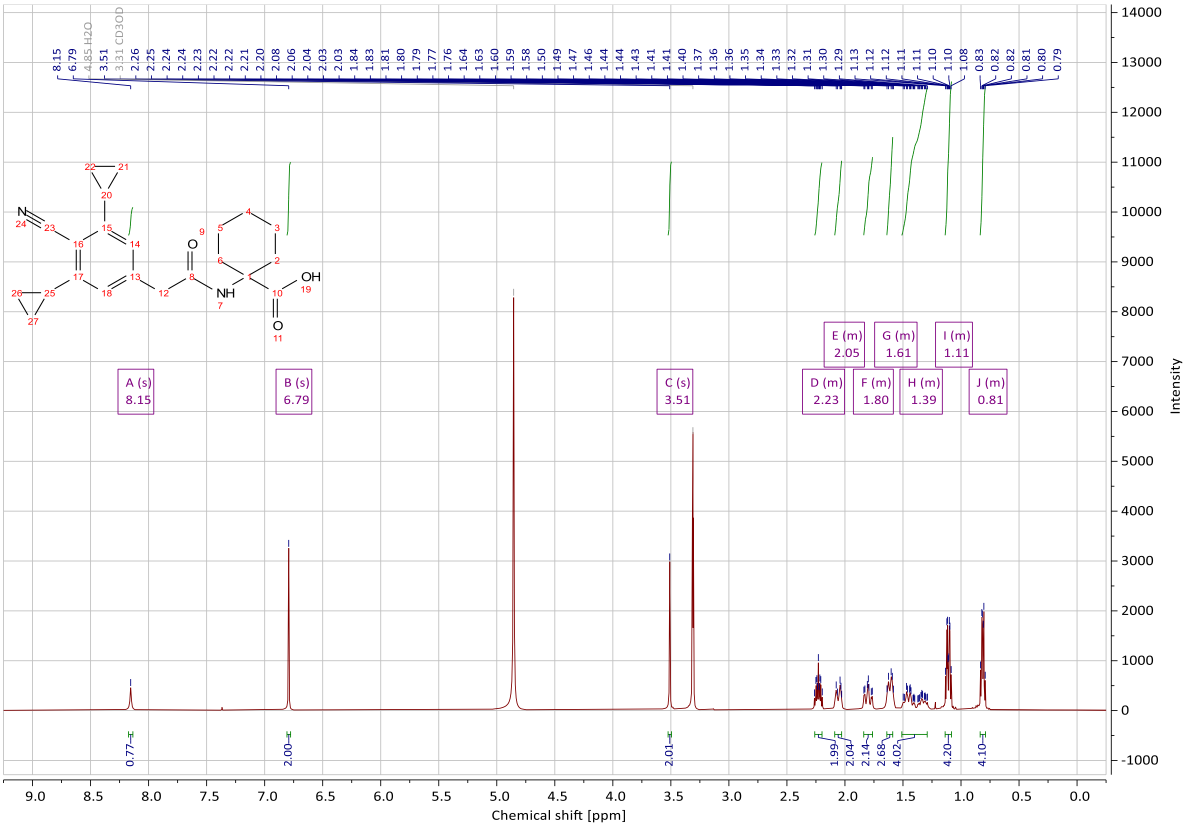

^{13}C-NMR:

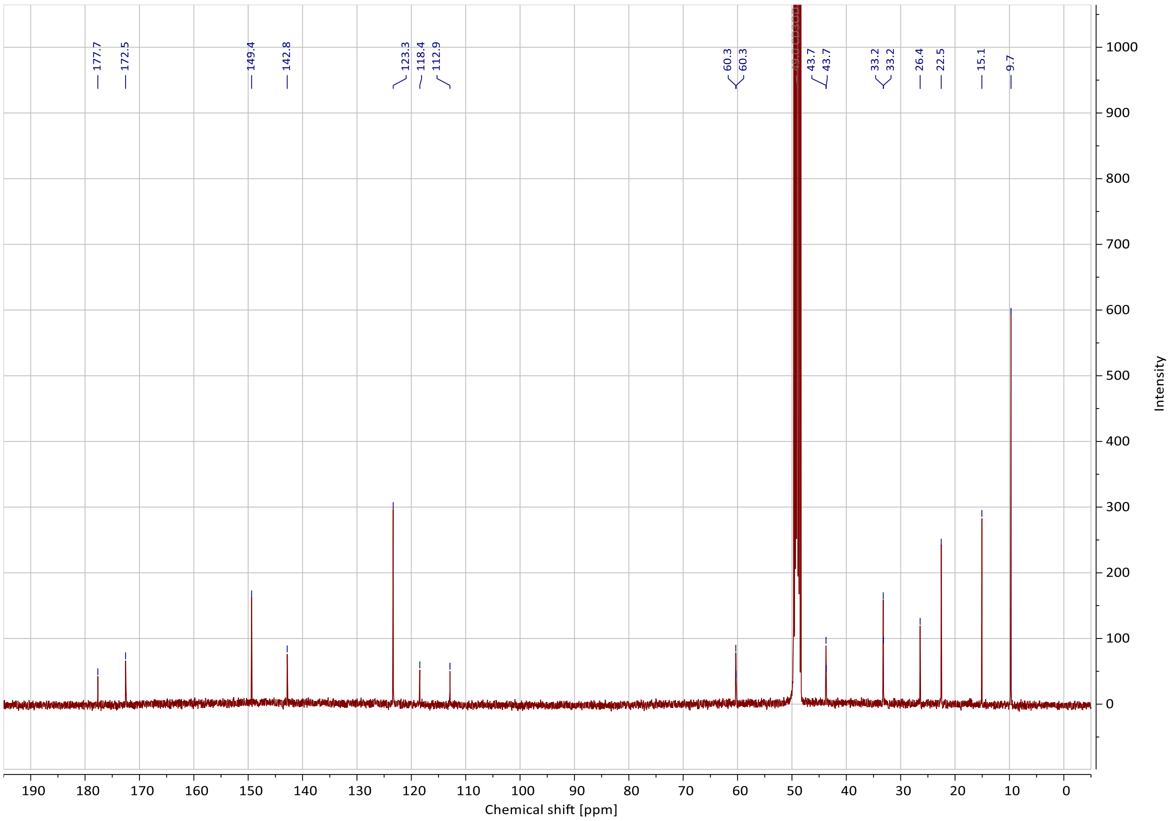

OP-AM (**4**)

^{1}H-NMR:

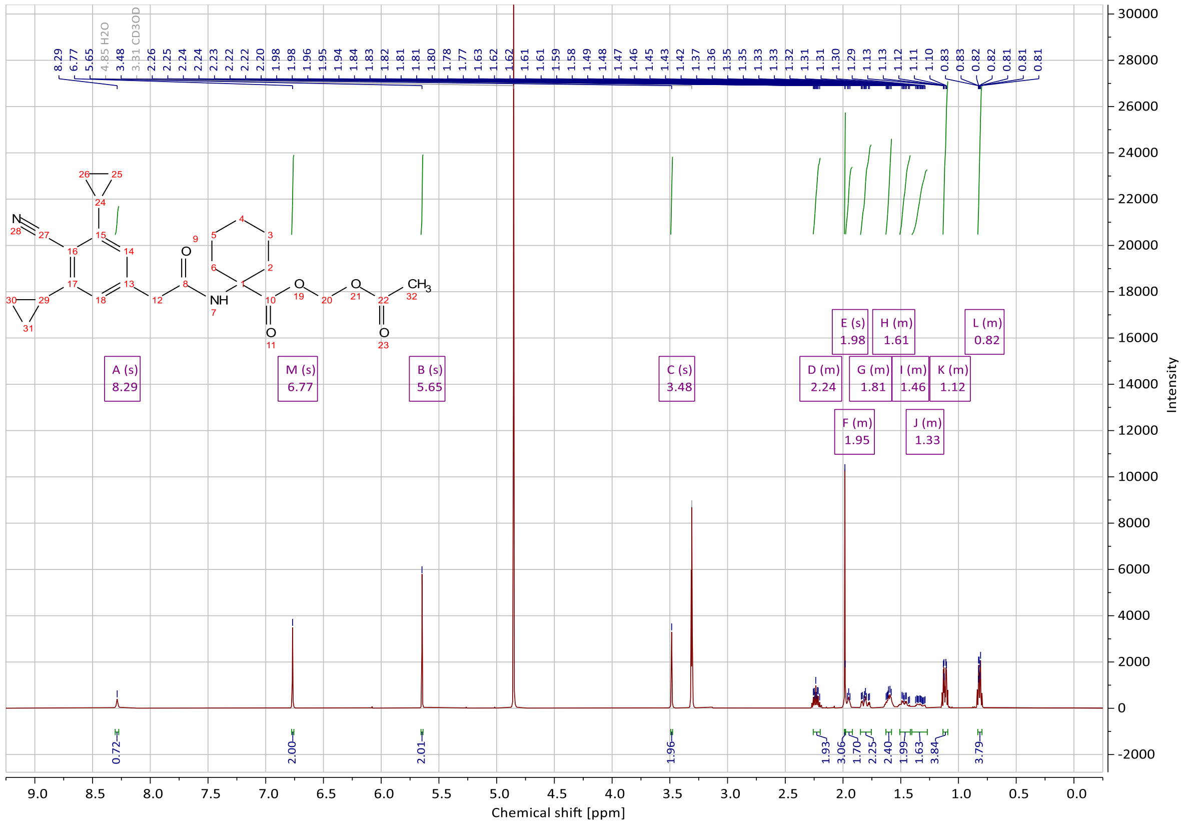

^{13}C-NMR:

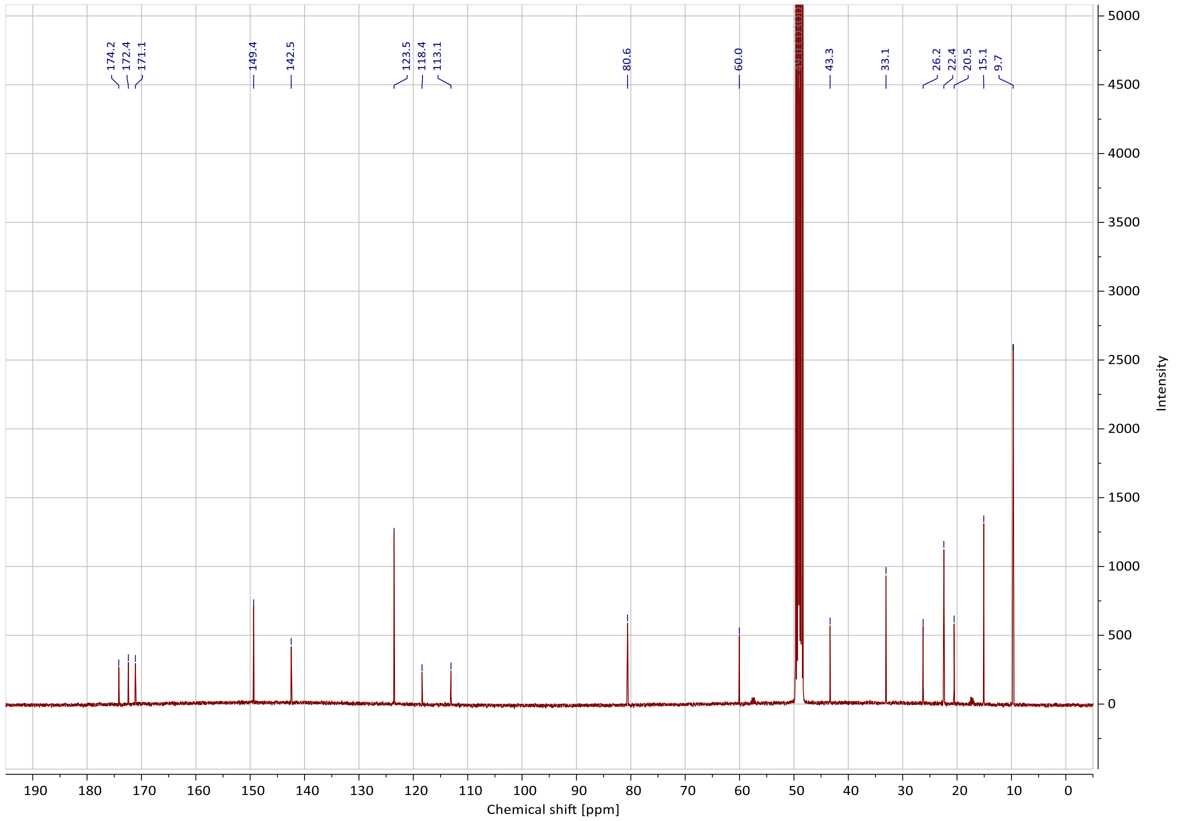

pOP (**5**)

^{1}H-NMR:

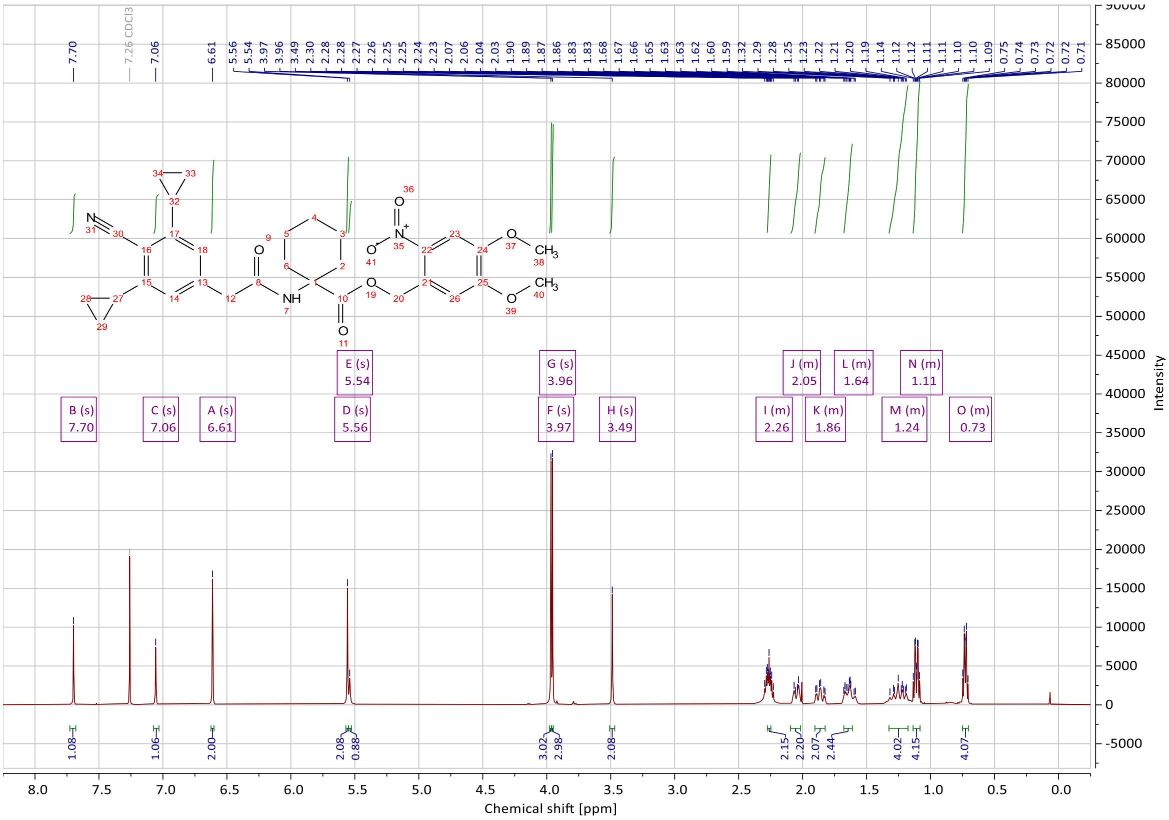

^{13}C-NMR:

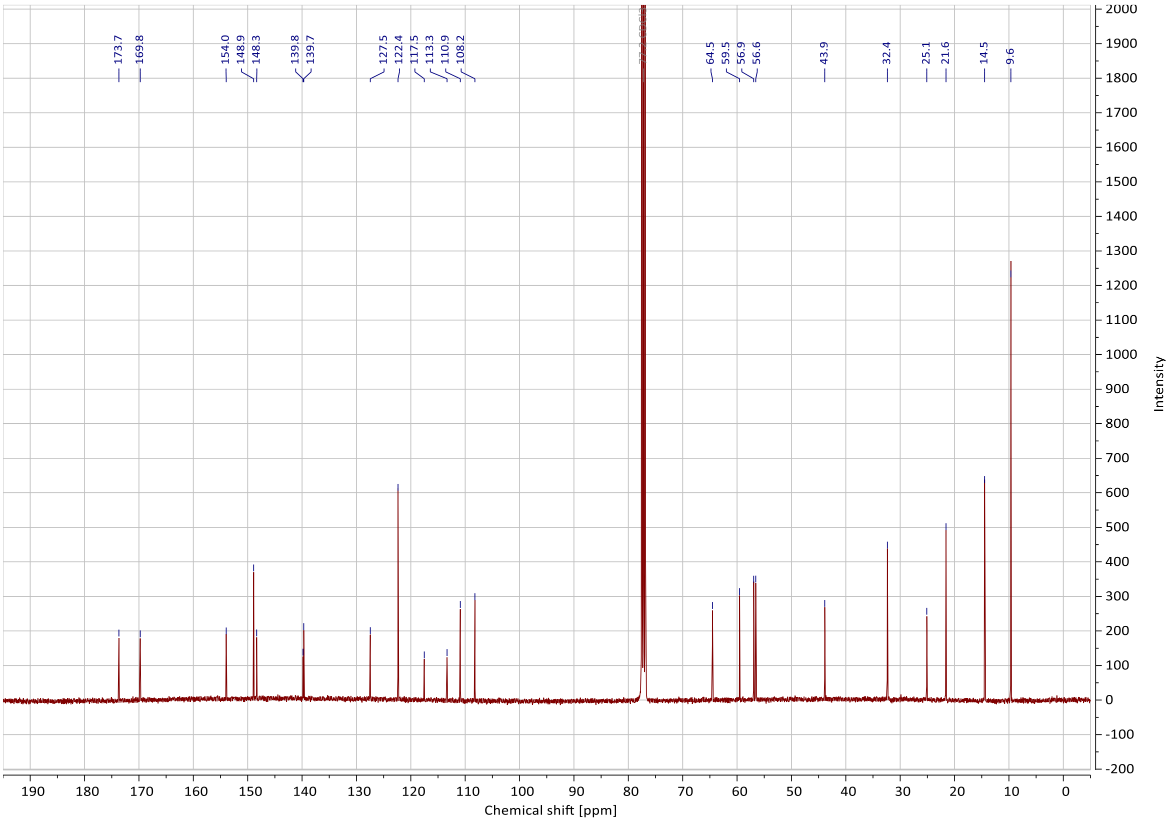

ABA-AM (**7**)

^{1}H-NMR:

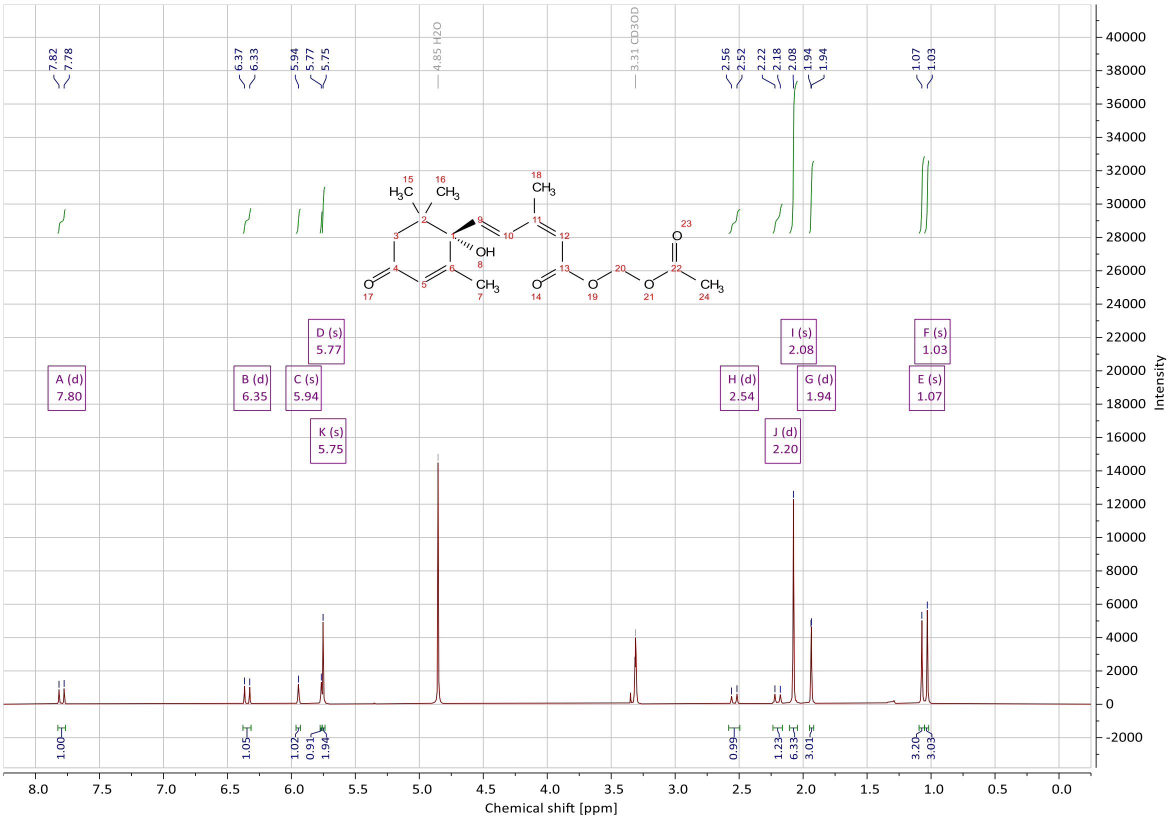

^{13}C-NMR:

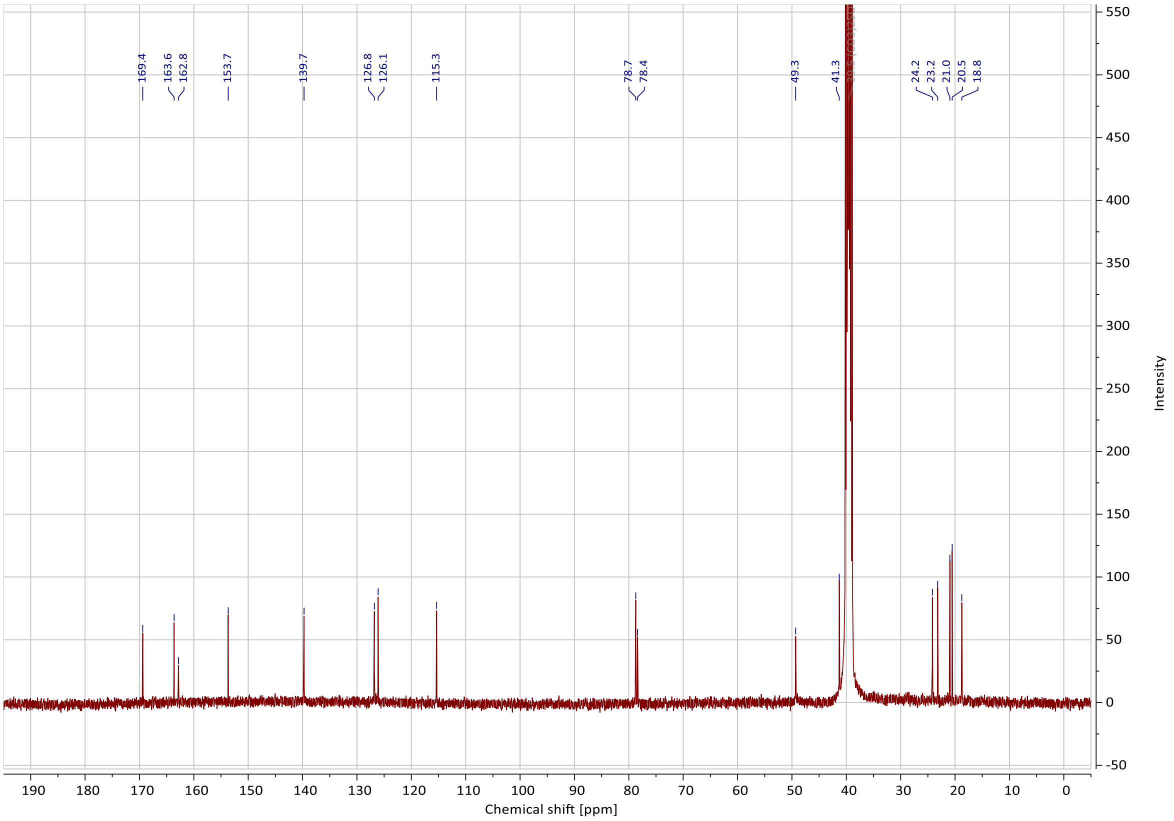

3 Author contributions

P.P., M.Z and R.W. designed this study. P.P. and C.B. conducted the chemical synthesis. C.K. helped with synthesis in the initial phase of the project. P.P. designed and cloned vectors, performed live-cell microscopy, luciferase assays and in vitro irradiation experiments. P.P. and K.P. conducted the medaka embryo experiments with support of J.W.. B.K. provided help with live-cell microscopy and cell culture. P.P. and R.W. wrote the manuscript with support of all authors. R.W. supervised the research.

4 Supplementary references

[1] M. V. George, J. C. Scaiano, *J. Phys. Chem.* **1980**, *84*, 492.

[2] M. J. Ziegler, K. Yserentant, V. Dunsing, V. Middel, A. J. Gralak, K. Pakari, J. Bargstedt, C. Kern, A. Petrich, S. Chiantia et al., *Nat. Chem. Biol.* **2021**.

[3] T. Iwamatsu, *Mech. Dev.* **2004**, *121*, 605.

[4] G. R. Fulmer, A. J. M. Miller, N. H. Sherden, H. E. Gottlieb, A. Nudelman, B. M. Stoltz, J. E. Bercaw, K. I. Goldberg, *Organometallics* **2010**, *29*, 2176.

[5] M. C. Bowden, T. C. Clark, F. D. B. Giordano, B. Jau, H. Schneider, G. Seifert, J. Wiss, M. Zeller, D. Faber, WO 2007/020381 A2, **2007**.

[6] M. Zeller, C. Lamberth, WO 2003/042168 A1, **2003**.

[7] C. Lamberth, F. Cederbaum, A. Jeanguenat, H.-J. Kempf, M. Zeller, R. Zeun, *Pest Manage. Sci.* **2006**, *62*, 446.

[8] A. S. Vaidya, J. D. M. Helander, F. C. Peterson, D. Elzinga, W. Dejonghe, A. Kaundal, S.-Y. Park, Z. Xing, R. Mega, J. Takeuchi et al., *Science (Washington, DC, U. S.)* **2019**, *366*.
